# Synthesis of fluoranthenes by hydroarylation of alkynes catalyzed by gold(I) or gallium trichloride

**DOI:** 10.3762/bjoc.7.178

**Published:** 2011-11-14

**Authors:** Sergio Pascual, Christophe Bour, Paula de Mendoza, Antonio M Echavarren

**Affiliations:** 1 Institute of Chemical Research of Catalonia (ICIQ), Av. Països Catalans 16, 43007 Tarragona, Spain; 2 Additional affiliation: Departament de Química Analítica i Química Orgànica, Universitat Rovira i Virgili, C/ Marcel·li Domingo s/n, 43007 Tarragona, Spain

**Keywords:** alkynes, gold(I) catalysis, hydroarylation, polyarenes

## Abstract

Electrophilic gold(I) catalyst **6** competes with GaCl_3_ as the catalyst of choice in the synthesis of fluoranthenes by intramolecular hydroarylation of alkynes. The potential of this catalyst for the preparation of polyarenes is illustrated by a synthesis of two functionalized decacyclenes in a one-pot transformation in which three C–C bonds are formed with high efficiency.

## Introduction

Electrophilic activation of alkynes in functionalized substrates by gold catalysts allows for the synthesis of complex molecules under mild conditions [1–8]. Alkynes can react in gold-catalyzed Friedel–Crafts-type reactions with arenes to give products resulting from the intermolecular hydroarylation of the alkynes (or alkenylation of the arenes) [9–21]. In addition to gold, the intramolecular version of this reaction was also carried out with Ru(II) [22], Pt(II) [12,22–23], Pt(IV) [24], Ga(III) [25–26], and Hg(II) [27–28] as catalysts.

Electron-rich indoles also react with alkynes in the presence of gold catalysts to form 6–8-membered rings [29–31]. A similar reaction can also be carried out with GaCl_3_ [32] and Pt(II) [33] as catalysts. In contrast, alkynyl furans react with gold to give phenols by using Au(III), Au(I) [1–2,34–37], or Pt(II) as the catalyst [38–39].

**Scheme 1:**
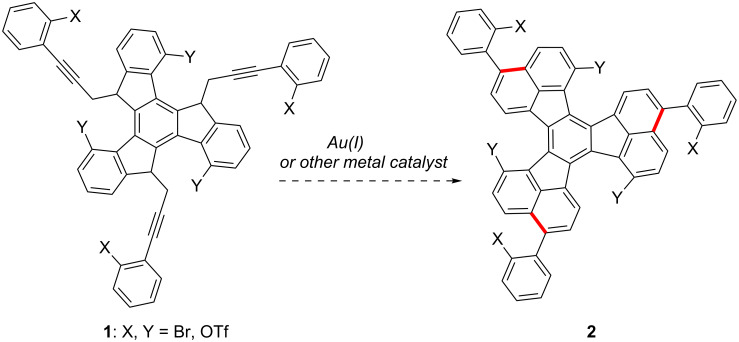
Proposed metal catalyzed annulation for the synthesis of triaryldiacenaphtho[1,2-*j*:1',2'-*l*]fluoranthenes **2**.

In our efforts towards the synthesis of large polyarenes [40–43], which are related to the fullerenes [44], we used the palladium-catalyzed arylation reaction as the main tool [45–48]. We decided to try the triple hydroarylation of substrates of type **1** to give 3,9,15-triaryldiacenaphtho[1,2-*j*:1',2'-*l*]fluoranthenes **2** with X and Y substitutes at strategic positions, which could be activated by palladium in subsequent intramolecular arylations (Scheme 1). Substituted fluoranthenes are of interest since some derivatives have been shown to be useful in light-emitting devices [49–52]. Fluoranthene derivatives have already been synthesized by palladium-catalyzed arylation reactions [53–54]. Strategically halogenated decacyclenes with a substitution pattern similar to that of **2** have been used for the synthesis of circumtrindene by flash vacuum pyrolysis [55]. Here we report the results on the synthesis of large polyarenes **2** and more simple 3-arylfluoranthenes by using gold(I)- or gallium(III)-catalyzed hydroarylation reactions.

## Results and Discussion

**Figure 1:**
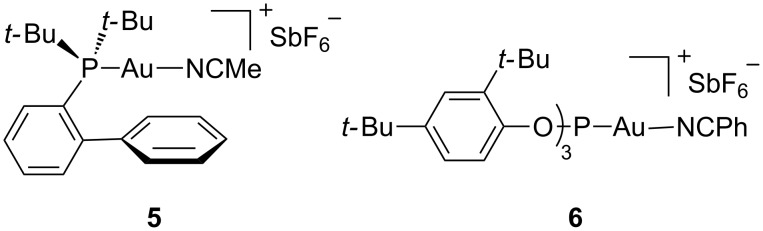
Cationic gold complexes **5** and **6**.

First, we examined the cyclization of **3** to give **4** or **4'** [22,24,26] (Table 1) with cationic gold(I) catalysts **5** [56] and **6** [57] (Figure 1), which have been demonstrated to be amongst the best catalysts in many gold(I)-catalyzed cyclizations [6,58]. No reaction was observed with complex **5** after heating for 5 min at 70 °C in CH_2_Cl_2_ under microwave irradiation (Table 1, entry 1), whereas the more electrophilic **6**, bearing a less donating phosphite ligand, led almost quantitatively to **4'** (Table 1, entry 2). Under these conditions, AuCl_3_ was not effective as a catalyst (Table 1, entry 3). As previously reported [25–26], GaCl_3_ is an excellent catalyst for the cyclization of **3** to give **4'** (Table 1, entry 4). In all cases the reaction proceeds exclusively though the 6-*exo*-*dig* pathway.

**Table 1:** Hydroarylation of **3** to give dihydronaphthalene **4'**.^a^

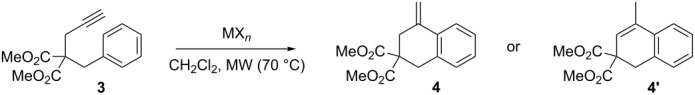		

entry	MX*_n_*	**4'** (yield, %)

1	**5**	—^b^
2	**6**	99
3	AuCl_3_	—^c^
4	GaCl_3_	99

^a^2 mol % catalyst, microwave irradiation, 5 min. ^b^100% **3** was recovered. ^c^87% **3** was recovered.

**Scheme 2:**
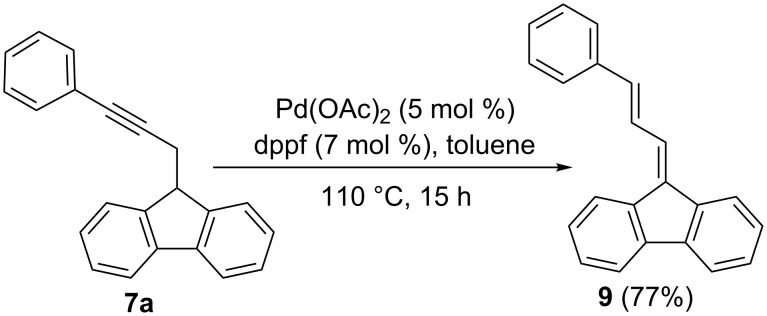
Pd(OAc)_2_-catalyzed isomerization of **7a** to form (*E*)-9-(3-phenylallylidene)-9*H*-fluorene (**9**).

The cyclization of 9-(3-phenylprop-2-ynyl)-9*H*-fluorene (**7a**) to form 3-phenylfluoranthene (**8a**) [59] was also examined by using catalysts **5**, **6**, and GaCl_3_ (Table 2). Since the initial gold(I)-catalyzed reaction provided a mixture of 3-phenyl-1,10b-dihydrofluoranthene, 3-phenyl-1,2,3,10b-tetrahydrofluoranthene, and **8a**, the crude mixtures were treated with excess DDQ in toluene under reflux to provide pure **8a**. No reaction or decomposition was observed when the reaction was carried out with gold(I) complex **5** (Table 2, entries 1 and 2). In contrast, the more electrophilic gold(I) complex **6** with phosphite as the ligand led to **8a** in 64–70% yield by stirring at room temperature in CH_2_Cl_2_ (Table 2, entries 3–5). Satisfactory results were obtained by simply using 1 mol % of **6** (Table 2, entry 5). No reaction was observed with PtCl_2_ or AuCl_3_ even after heating in toluene under reflux (Table 2, entries 3–5). Whereas InCl_3_ led to decomposition of **7a** under these conditions (Table 2, entry 6), GaCl_3_ led to **8a**, although satisfactory results were only obtained in toluene at 70 °C (Table 1, entry 10). Interestingly, FeCl_3_ was also catalytically active, although fluoranthene **8a** was only obtained in moderate yields (Table 2, entries 11 and 12). The reaction of **3a** with Pd(OAc)_2_ as catalyst proceeded differently to give known (*E*)-9-(3-phenylallylidene)-9*H*-fluorene (**9**) [60], presumably via the formation of the corresponding allene as an intermediate (Scheme 2).

**Table 2:** Hydroarylation of 9-(3-phenylprop-2-ynyl)-9*H*-fluorene (**7a**) to give 3-phenylfluoranthene (**8a**).^a^

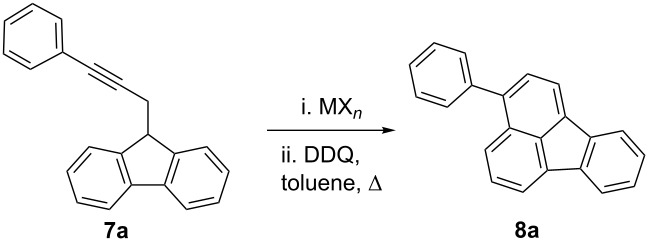					

entry	MX*_n_* (mol %)	solvent	*T* (ºC)	*t* (h)	yield (%)

1	**5** (2)	CH_2_Cl_2_	70^b^	0.7	—^c^
2	**5** (5)	toluene	110	1	—^d^
3	**6** (5)	CH_2_Cl_2_	r.t.	17	64
4	**6** (2)	CH_2_Cl_2_	r.t.	16	70
5	**6** (1)	CH_2_Cl_2_	r.t.	16	70
6	PtCl_2_ (5)	toluene	110	17	—^c^
7	AuCl_3_ (5)	toluene	110	17	—^c^
8	InCl_3_ (5)	toluene	110	17	—^d^
9	GaCl_3_ (2)	CH_2_Cl_2_	r.t.	26	16^e^
10	GaCl_3_ (2)	toluene	70^b^	0.2	57
11	FeCl_3_·6H_2_O (10)	DCE^f^	r.t.	40	36^e^
12	FeCl_3_·6H_2_O (5)	DCE^f^	70^b^	0.2	34^e^

^a^Crude reaction mixtures were aromatized by heating in toluene with DDQ (3 equiv) for 12 h. ^b^Microwave irradiation. ^c^No reaction. ^d^Product decomposition. ^e^Yield determined by ^1^H NMR. ^f^DCE = 1,2-dichloroethane.

Substrates **7b**–**j**, prepared by alkylation of fluorenyl lithium with the corresponding propargyl bromide or by Sonogashira couplings of 9-(prop-2-ynyl)-9*H*-fluorene [61], were cyclized by using gold(I) complex **6** or GaCl_3_ as the catalyst (Table 3). Although both catalysts could be used for the synthesis of 3-arylfluoranthenes **8b**–**h**, better yields were obtained with GaCl_3_ in toluene at 100 °C. However, in the case of 9-(3-bromoprop-2-yn-1-yl)-9*H*-fluorene (**7i**), gold(I) complex **6** gave more satisfactory results (Table 3, compare entries 10 and 11). The reaction proceeded satisfactorily with aryl-substituted substrates bearing either electron-donating (*p*-Me, *o*-OMe) or electron-withdrawing (*p*-Cl, *p*-Br, *p*-CN, *p*-NO_2_) groups. However, no reaction was observed for *n*-butyl derivative **7j** with **6** or with GaCl_3_ (Table 3, entries 12 and 13).

**Table 3:** Hydroarylation of **7b**–**j** to give 3-substituted fluoranthenes **8b**–**i**.^a^

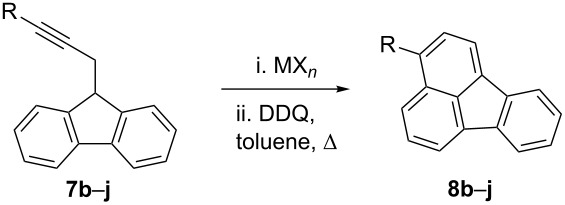							

entry	fluorene	R	MX*_n_* (mol %)	solvent	*T* (ºC)	*t* (h)	yield (%)

1	**7b**	*p*-Tol	GaCl_3_ (5)	toluene	100^b^	0.2	45
2	**7b**	*p*-Tol	**6** (5)	CH_2_Cl_2_	r.t.	17	28
3	**7c**	*p*-ClC_6_H_4_	GaCl_3_ (5)	toluene	100^b^	0.2	71
4	**7d**	*p*-NCC_6_H_4_	GaCl_3_ (2)	toluene	100^b^	0.2	88
5	**7e**	*p*-O_2_NC_6_H_4_	GaCl_3_ (2)	toluene	70^b^	0.2	92
6	**7f**	*o*-MeOC_6_H_4_	**6** (5)	CH_2_Cl_2_	r.t.	17	17
7	**7f**	*o*-MeOC_6_H_4_	GaCl_3_ (5)	toluene	100^b^	0.2	57
8	**7g**	*o*-BrC_6_H_4_	GaCl_3_ (5)	toluene	100^b^	0.2	44
9	**7h**	C_6_F_5_	GaCl_3_ (5)	toluene	100^b^	2	74
10	**7i**	Br	**6** (5)	CH_2_Cl_2_	r.t.	20	44
11	**7i**	Br	GaCl_3_ (5)	toluene	100^b^	0.2	21
12	**7j**	*n*-Bu	**6** (5)	CH_2_Cl_2_	r.t.	7	—^c^
13	**7j**	*n*-Bu	GaCl_3_ (2)	toluene	70^b^	0.2	—^c^

^a^Crude reaction mixtures were aromatized by heating in toluene with DDQ (3 equiv) for 12 h. ^b^Microwave irradiation. ^c^No reaction.

**Scheme 3:**
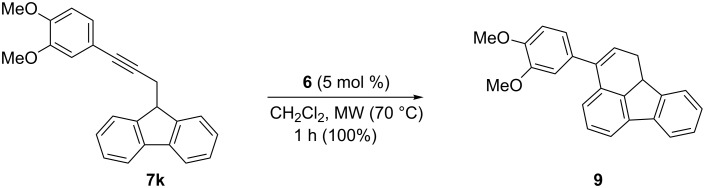
Gold(I)-catalyzed hydroarylation of **7k** to give 1,10b-dihydrofluoranthene **9**.

Cyclization of substrate **7k**, having an electron-rich aryl group at the alkyne, with catalyst **6** gave 1,10*b*-dihydrofluoranthene **9** cleanly in quantitative yield (Scheme 3).

**Scheme 4:**
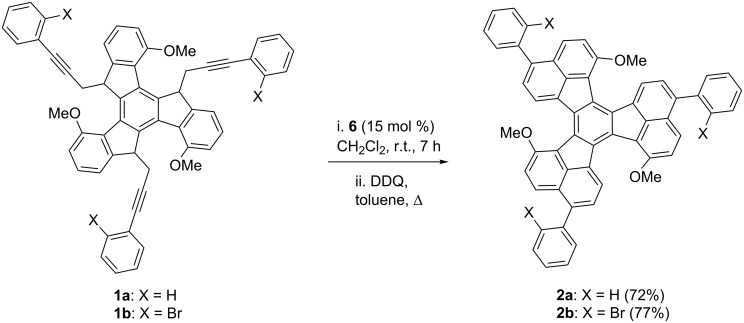
Gold(I)-catalyzed triple hydroarylation of **1a**,**b** to give **2a**,**b**.

Derivatives **1a** and **1b** were readily prepared by the triple alkylation of the lithium anion of 4,9,14-trimethoxytruxene (Scheme 4) [41,62]. The cyclization reaction was carried out efficiently with gold(I) catalyst **6** (15 mol %) at room temperature in CH_2_Cl_2_ to give triaryl substituted diacenaphtho[1,2-*j*:1',2'-l]fluoranthenes (decacyclenes) **2a** and **2b** in very good overall yields after aromatization of the crude products with DDQ. Remarkably, this triple hydroarylation occurs efficiently with an average yield per C–C bond formation that is greater than 90%.

## Conclusion

Highly electrophilic gold(I) catalyst **6** with a bulky phosphite ligand competes with GaCl_3_ as the catalyst of choice for the hydroarylation of alkynes. The synthetic potential of this catalyst is illustrated by the synthesis of functionalized triarylated decacyclenes in which three C–C bonds are formed with high efficiency in a one-pot transformation. The reaction is totally compatible with aryl bromides, which do not undergo subsequent arylation reaction due to the inertness of gold(I) catalysts towards oxidative addition reactions under homogeneous conditions [63–64].

## Supporting Information

Supporting Information features experimental details and characterization data for new compounds.

File 1 Experimental details
