# Supplementary material for: Synthesis of fluoranthenes by hydroarylation of alkynes catalyzed by gold(I) or gallium trichloride
Source: Beilstein J Org Chem. 2011 Nov 14;7:1520–5. doi: 10.3762/bjoc.7.178 (PMC3252853; doi:10.3762/bjoc.7.178)
Supplement: File 1 — Experimental details [file Beilstein_J_Org_Chem-07-1520-s001.pdf]

Supporting Information

for

Synthesis of fluoranthenes by hydroarylation of alkynes catalyzed by gold(I) or gallium trichloride

Sergio Pascual[1], Christophe Bour[1], Paula de Mendoza[1], and Antonio M. Echavarren*[1,2]

Address: [1]Institute of Chemical Research of Catalonia (ICIQ), Av. Països Catalans 16, 43007 Tarragona, Spain and [2]Additional affiliation: Departament de Química Analítica i Química Orgànica, Universitat Rovira i Virgili, C/ Marcel·li Domingo s/n, 43007 Tarragona (Spain)

Email: Antonio M. Echavarren* - aechavarren@iciq.es

*Corresponding author

Experimental details

Contents

General methods……………………………………………………………… S-2

Experimental results…………………………………………………………S-2

NMR Spectra………………………………………………………………S-16

General methods

All reactions were carried out under Ar in solvents dried in a Solvent Purification System (SPS). Thin layer chromatography was carried out by using TLC-aluminum sheets with 0.2 mm of silica gel (Merck GF_{234}). Chromatography purifications were carried out using flash-grade silica gel (SDS Chromatogel 60 ACC, 40–60 μm) or automated flash chromatographer CombyFlash Companion. NMR spectra were recorded at 23°C on a Bruker Avance 400 Ultrashield and Bruker Avance 500 Ultrashield apparatus. Mass spectra were recorded on a Waters LCT Premier (ESI) and Waters GCT (EI, CI) spectrometers. Elemental analyses were performed on a LECO CHNS 932 micro-analyzer at the Universidad Complutense de Madrid. Melting points were determined by using a Büchi melting-point apparatus.

Complexes **5** [1] and **6** [2] were prepared according to the reported procedures. Complex **5** is also available from Aldrich. Malonate **3** was prepared according to the described procedure [3].

Experimental results

Dimethyl 4-methylnaphthalene-2,2(1*H*)-dicarboxylate (4') [3].

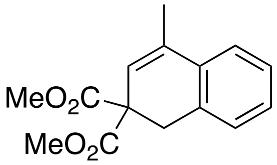

^{1}H NMR (400 MHz, $CDCl_3$) δ 7.22 (m, 4H), 5.97 (s, 1H), 3.71 (s, 6H), 3.37 (s, 2H), 2.13 (d, J = 1.3 Hz, 3H); ^{13}C NMR (101 MHz, $CDCl_3$) δ 170.90, 134.95, 133.29, 132.59, 127.90, 127.69, 126.91, 123.41, 121.05, 54.73, 52.87, 34.57, 19.38; HRMS - ESI Calcd for $C_{15}H_{16}O_4Na$ (M + Na): 283.0946 Found 283.0945.

Synthesis of fluorenes 7a–i

Procedure A (Propargylation of fluorene): *n*-BuLi 2.5M in hexanes (1.1 equiv) was added dropwise over a solution of fluorene (1.0 equiv) in anhydrous THF at −80 °C and the mixture was stirred at −80 °C for 3 hours. Then, a solution of the corresponding propargylbromide (1.0 equiv) in anhydrous THF was added dropwise and the mixture

was allowed to warm to r.t. overnight. Saturated NaCl solution was added and the organic layer was extracted with ethyl acetate (3×). The combined organic fractions were dried over anhydrous sodium sulfate, filtered and the solvent was evaporated under reduced pressure. The crude product was purified by flash chromatography (SiO_2) to give the corresponding alkynyl-fluorene.

Procedure B (Sonogashira Coupling): CuI (0.1 equiv) and [$PdCl_2(PPh_3)_2$] (0.5 equiv) were suspended in Et_3N and the resulting mixture was stirred for 5 min. The corresponding aryl bromide (1.3 equiv) and 9-(prop-2-ynyl)-9*H*-fluorene (1.0 equiv) in benzene were added sequentially. The reaction was stirred at 80 °C under microwave irradiation until TLC showed total conversion. The crude mixture was diluted with Et_2O, filtered through Celite and purified by flash chromatography (SiO_2) to give the corresponding alkynyl-fluorene.

9-(3-Phenylprop-2-ynyl)-9*H*-fluorene (7a).

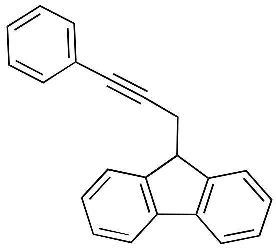

Prepared as a pale yellow solid by procedure A (yield = 87%).

R_f (2:1 hexane–CH_2Cl_2) = 0.60; mp = 70–71 °C. ^1H NMR ($CDCl_3$, 400 MHz) ™ 7.76 (d, J = 7.8 Hz, 4H), 7.30–7.43 (m, 9H), 4.15 (t, J = 7.3 Hz, 1H), 2.88 (d, J = 7.3 Hz, 2H). ^{13}C NMR ($CDCl_3$, 100 MHz, DEPT) ™ 146.3 (C), 140.8 (C), 131.6 (CH), 128.3 (CH), 127.8 (CH), 127.5 (CH), 127.0 (CH), 124.7 (CH), 123.7 (C), 119.8 (CH), 88.6 (C), 81.9 (C), 46.3 (CH), 24.5 (CH_2). Anal. calcd (%) for $C_{22}H_{16}$: C 94.25, H 5.75. Found: C 93.85, H 5.93.

9-(3-*p*-Tolylprop-2-ynyl)-9*H*-fluorene (7b).

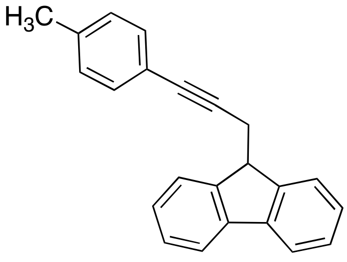

Prepared as a pale yellow solid by procedure B (yield = 52%).

R_f (3:1 Hexane–CH_2Cl_2) = 0.22; mp = 119–120 °C. ^1H NMR ($CDCl_3$, 400 MHz) ™ 7.76 (d, *J* = 8.0 Hz, 4H), 7.38 (t, *J* = 7.3 Hz, 2H), 7.32 (d, *J* = 8.0 Hz, 4H), 7.12 (d, *J* = 7.9 Hz, 2H), 4.14 (t, *J* = 7.4 Hz, 1H), 2.86 (d, *J* = 7.4 Hz, 2H), 2.35 (s, 3H). ^{13}C NMR ($CDCl_3$, 100 MHz, DEPT) ™ 146.3 (C), 140.8 (C), 137.8 (C), 131.4 (CH), 129.0 (CH), 127.5 (CH), 127.0 (CH), 124.7 (CH), 120.6 (C), 119.8 (CH), 87.9 (C), 81.9 (C), 46.3 (CH), 24.5 (CH_2), 21.4 (CH_3). HRMS: Calcd for $C_{23}H_{19}$ (M^++H) 295.1487, Found 295.1485.

9-(3-(4-Chlorophenyl)prop-2-ynyl)-9*H*-fluorene (7c).

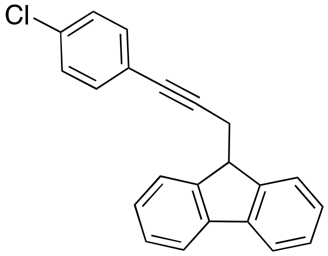

Prepared as a pale yellow solid by procedure B (yield = 61%).

R_f (3:1 hexane:CH_2Cl_2) = 0.43; mp = 142–143 °C. ^1H NMR ($CDCl_3$, 400 MHz) ™ 7.76 (d, *J* = 7.5 Hz, 2H), 7.72 (d, *J* = 7.5 Hz, 2H), 7.40 (t, *J* = 7.4 Hz, 2H), 7.25–7.34 (m, 6H), 4.14 (t, *J* = 7.2 Hz, 1H), 2.89 (d, *J* = 7.2 Hz, 2H). ^{13}C NMR ($CDCl_3$, 100 MHz, DEPT) ™ 146.1 (C), 140.9 (C), 133.8 (C), 132.8 (CH), 128.6 (CH), 127.6 (CH), 127.1 (CH), 124.6 (CH), 122.1 (C), 119.9 (CH), 89.6 (C), 80.8 (C), 46.1 (CH), 24.5 (CH_2). HRMS: Calcd for $C_{22}H_{16}{}^{35}Cl$ (M^++H) 315.0941, Found 315.0944.

4-(3-(9*H*-Fluoren-9-yl)prop-1-ynyl)benzonitrile (7d)

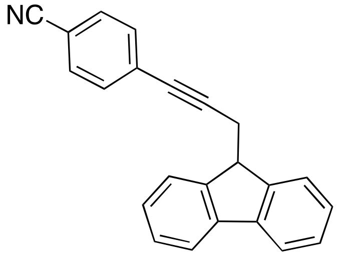

Prepared as a white solid by procedure B (yield = 60%).

R_f (2:1 hexane–CH_2Cl_2) = 0.15; mp = 152–153 °C. ^1H NMR ($CDCl_3$, 400 MHz) ™ 7.77 (d, *J* = 7.3 Hz, 2H), 7.69 (d, *J* = 7.5 Hz, 2H), 7.56 (d, *J* = 8.3 Hz, 2H), 7.58 (dt, *J* = 8.3, 1.6 Hz, 2H), 7.39–7.42 (m, 4H), 7.33 (dt, *J* = 7.4, 1.2 Hz, 2H), 4.16 (t, *J* = 7.2 Hz, 1H),

2.96 (d, *J* = 7.2 Hz, 2H). ^{13}C NMR ($CDCl_3$, 100 MHz, DEPT) ™ 145.9 (C), 140.9 (C), 132.1 (CH), 132.0 (CH), 128.6 (C), 127.7 (CH), 127.1 (CH), 124.5 (CH), 119.9 (CH), 118.5 (C), 111.1 (C), 93.5 (C), 80.6 (C), 45.9 (CH), 24.5 (CH_2). HRMS: Calcd for $C_{24}H_{20}NO$ (M^++H+MeOH) 338.1545, Found 338.1555.

9-(3-(4-Nitrophenyl)prop-2-ynyl)-9*H*-fluorene (7e)

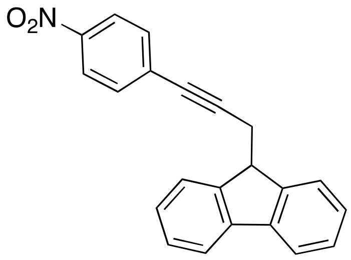

Prepared as a pale yellow solid following procedure B (yield = 79%).

R_f (2:1 hexane–CH_2Cl_2): 0.30; mp = 135–136 °C. ^{1}H NMR ($CDCl_3$, 400 MHz) δ 8.16 (d, *J* = 8.9 Hz, 2H), 7.78 (d, *J* = 8.9 Hz, 2H), 7.70 (d, *J* = 7.2 Hz, 2H), 7.47 (d, *J* = 8.9, 1.9 Hz, 2H), 7.42 (t, *J* = 7.3 Hz, 2H), 7.34 (dd, *J* = 7.3, 6.3 Hz, 2H), 4.18 (t, *J* = 7.0 Hz, 1H), 2.98 (d, *J* = 7.0 Hz, 2H). ^{13}C NMR ($CDCl_3$, 100 MHz) δ 145.8, 141.0, 132.3, 130.6, 127.7, 127.1, 124.5, 123.5, 120.0, 94.5, 80.5, 45.9, 24.6. HRMS: Calcd for $C_{22}H_{16}NO_2$ (M^++H) 326.1181, Found 326.1169.

9-(3-(2-Methoxyphenyl)prop-2-ynyl)-9*H*-fluorene (7f)

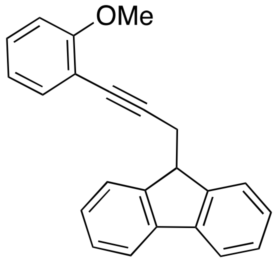

Prepared as a white solid following procedure B (yield = 45%).

R_f (4:1 hexane–CH_2Cl_2): 0.21; mp = 109–110 °C. ^{1}H NMR ($CDCl_3$, 400 MHz) δ 7.87 (d, *J* = 7.2 Hz, 2H), 7.76 (d, *J* = 7.5 Hz, 2H), 7.37–7.42 (m, 3H), 7.39 (dt, *J* = 7.9, 1.8 Hz, 1H), 7.32 (dt, *J* = 7.5, 1.1 Hz, 2H), 6.92 (dt, *J* = 7.5, 1.1 Hz, 1H), 6.90 (d, *J* = 8.0 Hz, 1H), 4.19 (t, *J* = 7.6 Hz, 1H), 3.92 (s, 3H), 2.92 (d, *J* = 7.6 Hz, 2H). ^{13}C NMR ($CDCl_3$, 100 MHz, DEPT) δ 160.1 (C), 146.4 (C), 140.8 (C), 133.5 (CH), 129.2 (CH), 127.4 (CH), 127.0 (CH), 124.9 (CH), 120.4 (CH), 119.8 (CH), 112.9 (C), 110.6 (CH),

92.9 (C), 78.1 (C), 55.7 (CH_3), 46.4 (CH), 24.8 (CH_2). HRMS: Calcd for $C_{23}H_{19}O$ (M^++H) 311.1436, Found 311.1427.

9-(3-(2-Bromophenyl)prop-2-ynyl)-9*H*-fluorene (7g).

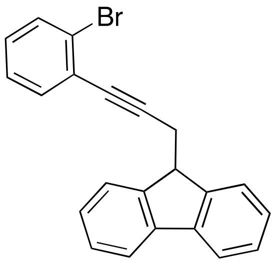

Prepared as a colorless oil by procedure A (yield = 74%) or procedure B (yield = 41%).

R_f (3:1 hexane–CH_2Cl_2) = 0.60. ^{1}H NMR ($CDCl_3$, 400 MHz) ™ 7.80 (d, *J* = 7.5 Hz, 2H), 7.76 (d, *J* = 7.5 Hz, 2H), 7.56 (d, *J* = 8.0 Hz, 1H), 7.37–7.43 (m, 3H), 7.31–7.34 (m, 2H), 7.22–7.25 (m, 1H), 7.11–7.15 (m, 1H), 4.19 (t, *J* = 7.1 Hz, 1H), 2.97 (d, *J* = 7.1 Hz, 2H). ^{13}C NMR ($CDCl_3$, 100 MHz, DEPT) ™ 146.1 (C), 140.9 (C), 133.5 (CH), 132.3 (CH), 128.9 (CH), 127.5 (CH), 127.1 (CH), 126.9 (CH), 125.7 (C), 125.4 (C), 124.8 (CH), 119.9 (CH), 93.5 (C), 80.5 (C), 46.1 (CH), 24.6 (CH_2). HRMS: Calcd for $C_{22}H_{16}{}^{79}Br$ (M^++H) 359.0435, Found 359.0441.

9-(3-(Pentafluorophenyl)prop-2-ynyl)-9*H*-fluorene (7h)

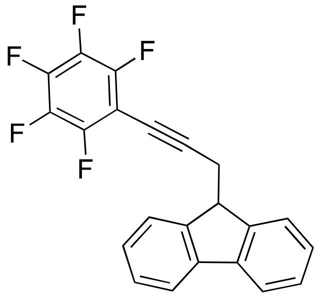

Prepared as a white solid following procedure B (yield = 57%).

R_f (4:1 hexane–CH_2Cl_2): 0.50; mp = 128–129 °C. ^{1}H NMR ($CDCl_3$, 400 MHz) δ 7.76 (d, *J* = 7.5 Hz, 2H), 7.72 (d, *J* = 7.5 Hz, 2H), 7.41 (t, *J* = 7.3 Hz, 2H), 7.33 (dt, *J* = 7.4, 1.2 Hz, 2H), 4.17 (t, *J* = 7.1 Hz, 1H), 2.99 (d, *J* = 7.1 Hz, 2H). ^{13}C NMR ($CDCl_3$, 100 MHz, DEPT) δ 145.6 (C), 140.9 (C), 127.7 (CH), 127.1 (CH), 124.5 (CH), 120.0 (CH), 101.9 (C), 66.0 (C), 45.6 (CH), 24.8 (CH_2). HRMS: Calcd for $C_{22}H_{12}F_5$ (M^++H) 371.0859, Found 371.0867.

9-(3-Bromoprop-2-ynyl)-9*H*-fluorene (7i)

Prepared by reaction of 9-(prop-2-ynyl)-9*H*-fluorene with NBS and $AgNO_3$ in acetone at room temperature in the dark in 87% yield.

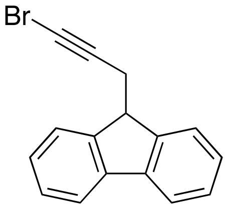

R_f (Hexane): 0.21; mp = pale yellow oil. ^{1}H NMR ($CDCl_3$, 400 MHz) ™ 7.74 (d, J = 7.5 Hz, 2H), 7.66 (d, J = 7.5 Hz, 2H), 7.39 (dd, J = 7.5 Hz, J = 7.5 Hz, 2H), 7.32 (ddd, J = 7.5 Hz, J = 7.5 Hz, J = 1.1 Hz, 2H), 4.05 (t, J = 7.4 Hz, 1H), 2.67 (d, J = 7.4 Hz, 2H). ^{13}C NMR ($CDCl_3$, 100 MHz, DEPT) ™ 145.9 (C), 140.8 (C), 127.6 (CH), 127.1 (CH), 124.6 (CH), 119.9 (CH), 78.9 (C), 45.8 (CH), 39.6 (C-Br), 24.7 (CH_2)

9-(Hept-2-ynyl)-9*H*-fluorene (7j)

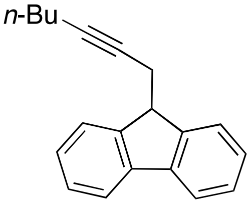

Prepared as a pale yellow oil following procedure A (yield = 89%).

R_f (5.1 hexane:CH_2Cl_2): 0.46. ^{1}H NMR ($CDCl_3$, 400 MHz) δ 7.74 (d, J = 7.5 Hz, 2H), 7.70 (d, J = 7.4 Hz, 2H), 7.37 (dd, J = 7.4 Hz, J = 7.4 Hz, 2H), 7.31 (dd, J = 7.4 Hz, J = 7.4 Hz, 2H), 4.02 (t, J = 7.1 Hz, 1H), 2.63–2.66 (m, 2H), 2.17–2.21 (m, 2H), 1.32–1.51 (m, 4H), 0.90 (t, J = 7.3 Hz, 3H). ^{13}C NMR ($CDCl_3$, 100 MHz, DEPT) δ 146.5 (C), 140.8 (C), 127.3 (CH), 126.9 (CH), 124.6 (CH), 119.7 (CH), 81.7 (C), 78.4 (C), 46.5 (CH), 30.0 (CH_2), 23.8 (CH_2), 21.9 (CH_2), 18.5 (CH_2), 13.7 (CH_3). HRMS: Calcd for $C_{20}H_{19}$ (M^+-H) 259.1487, Found 259.1483.

9-(3-(3,4-Dimethoxyphenyl)prop-2-ynyl)-9*H*-fluorene (7k)

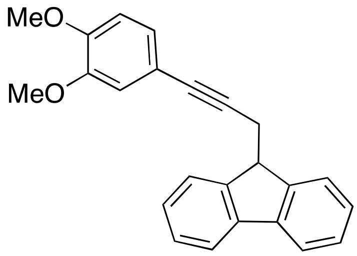

Prepared as a pale orange solid following procedure B (yield = 85%).

^{1}H NMR ($CDCl_3$, 400 MHz) δ 7.75–7.77 (m, 4H), 7.40 (dd, *J* = 7.7, 7.2 Hz, 2H), 7.33 (dt, *J* = 7.4, 1.0 Hz, 2H), 7.02 (dd, *J* = 8.3, 1.9 Hz, 1H), 6.90 (d, *J* = 1.9 Hz, 1H), 6.81 (d, *J* = 8.3 Hz, 1H), 4.16 (t, *J* = 7.3 Hz, 1H), 3.89 (s, 3H), 3.88 (s, 3H), 2.88 (d, *J* = 7.3 Hz, 2H). ^{13}C NMR ($CDCl_3$, 100 MHz, DEPT) δ 149.1 (C), 148.6 (C), 146.3 (2C), 140.9 (C), 127.5 (2CH), 127.0 (2CH), 124.7 (2CH), 124.6 (CH), 119.8 (2CH), 115.9 (C), 114.4 (CH), 111.0 (CH), 87.0 (C), 81.8 (C), 55.9 ($2CH_3$), 46.3 (CH), 24.5 (CH_2). HRMS: Calcd for $C_{24}H_{20}O_2Na$ (M^++Na) 363.1361, Found 363.1364.

General procedure for the hydroarylation reaction

A mixture of alkynylfluorene and the cationic gold catalyst or $GaCl_3$ in anhydrous CH_2Cl_2 (ca. 0.2 M) was stirred at the stated temperature (Tables 2 and 3). The solvent was evaporated under reduced pressure and the crude product was dissolved in toluene (ca. 0.1 M). This solution was added over a solution of DDQ (3 equiv) in toluene (final concentration ca. 0.03 M) and the mixture was heated under reflux overnight. The solvent was evaporated and the crude product was purified by flash chromatography (SiO_2, eluent: hexane).

Microwave irradiations were carried out in sealed tubes in a Biotage Initiator apparatus.

3-Phenylfluoranthene (8a) [4]

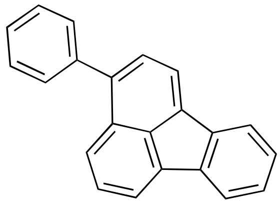

Prepared as a pale yellow solid with **6** as catalyst (yield = 70%).

R_f (hexane:CH_2Cl_2/3:1): 0.63; mp = 146–147 °C. ^{1}H NMR ($CDCl_3$, 400 MHz) δ 7.92–7.99 (m, 5H), 7.60–7.63 (m, 4H), 7.51–7.54 (m, 2H), 7.37–7.47 (m, 3H). ^{13}C NMR

($CDCl_3$, 100 MHz, DEPT) δ 140.3 (C), 139.8 (C), 139.5 (C), 139.1 (C), 137.1 (C), 136.3 (C), 132.7 (C), 130.3 (CH), 128.6 (CH), 128.3 (CH), 128.1 (CH), 127.6 (CH), 127.5 (CH), 127.4 (CH), 125.6 (CH), 121.5 (CH), 121.4 (CH), 120.0 (CH). Elemental analysis calcd (%) for $C_{22}H_{14}$: C 94.93, H 5.07; found: C 94.50, H 5.29

3-*p*-Tolylfluoranthene (8b)

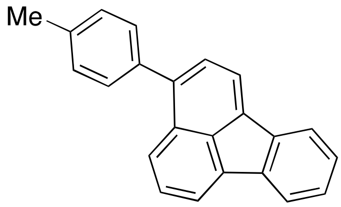

Prepared as a yellow solid with $GaCl_3$ (yield = 45%) or **6** (yield = 28%) as catalyst.

R_f (4:1 hexane–CH_2Cl_2): 0.61; mp = 147–148 °C. ^{1}H NMR ($CDCl_3$, 400 MHz) δ 7.90–7.97 (m, 5H), 7.60 (t, *J* = 7.8 Hz, 2H), 7.50 (d, *J* = 7.6 Hz, 2H), 7.37–7.39 (m, 2H), 7.33 (d, *J* = 7.6 Hz, 2H), 2.46 (s, 3H). ^{13}C NMR ($CDCl_3$, 100 MHz, DEPT) δ 140.3 (C), 139.5 (C), 139.1 (C), 137.1 (C), 137.0 (C), 136.8 (C), 136.1 (C), 130.1 (CH), 129.1 (CH), 128.5 (CH), 127.9 (CH), 127.5 (CH), 127.4 (CH), 125.7 (CH), 121.4 (CH), 121.3 (CH), 120.0 (CH), 119.9 (CH), 21.2 (CH_3). HRMS: Calcd for $C_{23}H_{17}$ (M^++H) 293.1330, Found 293.1324

3-(4-Chlorophenyl)fluoranthene (8c)

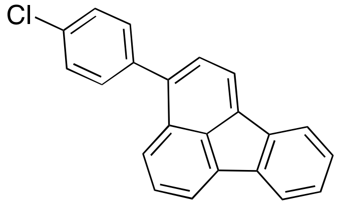

Prepared as a yellow solid with $GaCl_3$ as catalyst (yield = 71%).

R_f (4.1 hexane:CH_2Cl_2): 0.54; mp = 188–189 °C. ^{1}H NMR ($CDCl_3$, 400 MHz) δ 7.92–7.98 (m, 4H), 7.86 (d, *J* = 8.2 Hz, 1H), 7.62 (dd, *J* = 8.4, 6.8 Hz, 1H), 7.57 (d, *J* = 7.1 Hz, 1H), 7.48–7.52 (m, 4H), 7.39–7.41 (m, 2H). ^{13}C NMR ($CDCl_3$, 100 MHz, DEPT) δ 139.5 (C), 139.0 (C), 138.9 (C), 138.2 (C), 137.2 (C), 136.7 (C), 133.6 (C), 132.7 (C), 131.5 (CH), 128.6 (CH), 128.5 (CH), 128.3 (CH), 128.2 (C), 127.7 (CH), 127.6 (CH), 125.2 (CH), 121.5 (CH), 121.4 (CH), 120.1 (CH), 119.9 (CH). HRMS: Calcd for $C_{22}H_{13}{}^{35}Cl$ (M^+) 312.0700, Found 312.0715.

4-(Fluoranthen-3-yl)benzonitrile (8d)

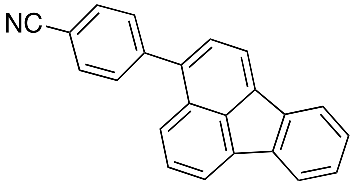

Prepared as a solid with $GaCl_3$ as catalyst (yield = 88%).

^{1}H NMR ($CDCl_3$, 400 MHz) *d* 8.00 (d, *J* = 7.2 Hz, 1H), 7.98 (d, *J* = 6.9 Hz, 1H), 7.92–7.95 (m, 2H), 7.80–7.83 (m, 3H), 7.71–7.74 (m, 2H), 7.65 (dd, *J* = 8.4, 6.9 Hz, 1H), 7.60 (d, *J* = 7.1 Hz, 1H), 7.41–7.43 (m, 2H). ^{13}C NMR ($CDCl_3$, 100 MHz) *d* 144.8, 139.8, 139.0, 138.1, 137.8, 137.6, 132.9, 132.4, 131.1, 129.1, 128.9, 128.1, 128.0, 124.9, 121.9, 121.8, 120.6, 120.0, 119.1, 111.4 (CH).

3-(4-Nitrophenyl)fluoranthene (8e)

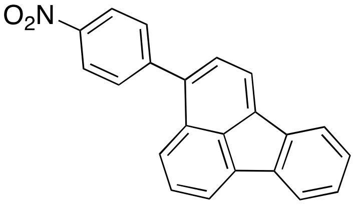

Prepared as a solid with $GaCl_3$ as catalyst (yield = 92%).

^{1}H NMR ($CDCl_3$, 400 MHz) δ 8.40 (dd, *J* = 8.6, 1.8 Hz, 2H), 8.00 (d, *J* = 7.2 Hz, 1H), 7.98 (d, *J* = 6.9 Hz, 1H), 7.91–7.96 (m, 2H), 7.83 (d, *J* = 8.5 Hz, 1H), 7.78 (dd, *J* = 8.6, 1.8 Hz, 2H), 7.66 (dd, *J* = 8.6, 7.9 Hz, 1H), 7.63 (d, *J* = 7.1 Hz, 1H), 7.40–7.45 (m, 2H).

3-(2-Methoxyphenyl)fluoranthene (8f)

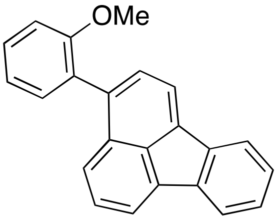

Prepared as a pale yellow solid with $GaCl_3$ (yield = 57%) or **6** (yield = 17%) as catalyst.

R_f (2:1 hexane–CH_2Cl_2): 0.30; mp = 162–163 °C. ^{1}H NMR ($CDCl_3$, 400 MHz) δ 7.95 (d, *J* = 7.1 Hz, 1H), 7.88–7.91 (m, 3H), 7.51–7.62 (m, 3H), 7.42 (dt, *J* = 7.7, 1.7 Hz, 1H), 7.35–7.37 (m, 3H), 7.09 (t, *J* = 7.4 Hz, 1H), 7.05 (d, *J* = 8.3 Hz, 1H), 3.71 (s, 3H).

^{13}C NMR ($CDCl_3$, 100 MHz, DEPT) δ 139.7 (C), 139.4 (C), 137.1 (C), 137.0 (C), 136.4 (C), 132.5 (C), 132.4 (CH), 129.4 (CH), 129.3 (C), 129.2 (CH), 128.6 (C), 127.6 (CH), 127.5 (CH), 127.4 (CH), 126.1 (CH), 121.5 (CH), 121.4 (CH), 120.6 (CH), 119.9 (CH), 119.8 (CH), 111.2 (CH). HRMS: Calcd for $C_{23}H_{17}O$ (M^++H) 309.1279, Found 309.1269.

3-(2-Bromophenyl)fluoranthene (8g) [5]

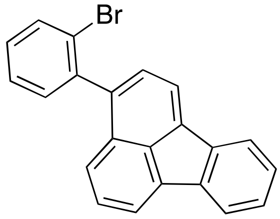

Prepared as a pale yellow oil with $GaCl_3$ as catalyst (yield = 44%).

R_f (4:1 hexane–CH_2Cl_2): 0.32. ^1H NMR ($CDCl_3$, 400 MHz) δ 7.97 (d, *J* = 7.1 Hz, 1H), 7.89–7.93 (m, 3H), 7.75 (d, *J* = 8.0 Hz, 1H), 7.48–7.58 (m, 3H), 7.37–7.43 (m, 4H), 7.29–7.33 (m, 1H). ^{13}C NMR ($CDCl_3$, 100 MHz, DEPT) δ 140.3 (C), 139.7 (C), 139.2 (C), 139.0 (C), 137.1 (C), 136.9 (C), 132.8 (CH), 132.4 (C), 132.3 (CH), 129.2 (CH), 129.0 (CH), 128.7 (C), 128.1 (CH), 127.6 (CH), 127.0 (CH), 125.5 (CH), 124.1 (C), 121.5 (CH), 120.1 (CH), 119.6 (CH). HRMS: Calcd for $C_{22}H_{14}{}^{79}Br$ (M^++H) 357.0279, Found 357.0271

3-(Pentafluorophenyl)fluoranthene (8h)

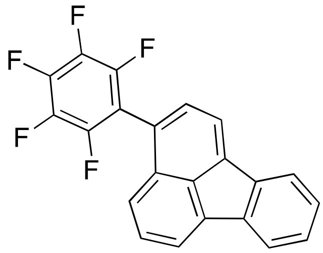

Prepared as a pale yellow solid with $GaCl_3$ as catalyst (yield = 74%).

R_f (4:1 hexane–CH_2Cl_2): 0.57; ^1H NMR ($CDCl_3$, 400 MHz) δ 8.00 (d, *J* = 7.2 Hz, 1H), 7.95 (d, *J* = 6.8 Hz, 1H), 7.90–7.93 (m, 2H), 7.58–7.65 (m, 2H), 7.48 (dt, *J* = 8.4, 1.6 Hz, 1H), 7.38–7.43 (m, 2H). ^{13}C NMR ($CDCl_3$, 100 MHz, DEPT) δ 139.8 (C), 139.1 (C), 139.0 (C), 137.7 (C), 132.8 (C), 131.1 (CH), 129.2 (CH), 128.8 (C), 128.5 (CH), 128.1 (CH), 124.4 (CH), 123.4 (C), 122.1 (CH), 121.9 (CH), 120.8 (CH), 119.7 (CH). HRMS: Calcd for $C_{22}H_9F_5$ (M^+) 368.0619, Found 368.0645.

3-Bromofluoranthene (8i)

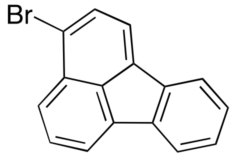

Prepared as a pale yellow solid with $GaCl_3$ (yield = 21%) or **6** (yield = 44%) as catalyst.

R_f (3:1 hexane–CH_2Cl_2): 0.67; mp = 111–112 °C. ^{1}H NMR ($CDCl_3$, 400 MHz) δ 7.94 (d, *J* = 8.4 Hz, 1H), 7.86 (d, *J* = 6.9 Hz, 1H), 7.78–7.83 (m, 2H), 7.76 (d, *J* = 7.4 Hz, 1H), 7.61–7.66 (m, 2H), 7.31–7.38 (m, 2H). ^{13}C NMR ($CDCl_3$, 100 MHz, DEPT) δ 139.0 (C), 138.7 (C), 137.2 (C), 136.7 (C), 133.5 (C), 131.1 (CH), 129.8 (C), 129.1 (CH), 127.9 (CH), 125.9 (CH), 121.8 (C), 121.6 (CH), 121.5 (CH), 120.7 (CH), 120.6 (CH). HRMS: Calcd for $C_{16}H_9{}^{79}Br$ (M^+) 279.9882, Found 279.9894.

3-(3,4-Dimethoxyphenyl)-1,10b-dihydrofluoranthene (9)

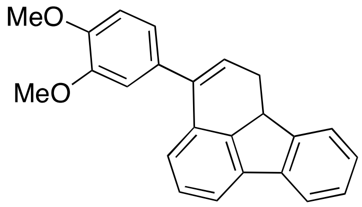

^{1}H NMR ($CDCl_3$, 400 MHz) δ 7.81 (d, *J* = 7.5, 1H), 7.65 (d, *J* = 7.2, 1H), 7.58 (d, *J* = 7.7, 1H), 7.41 (t app, *J* = 7.2 Hz, 1H), 7.07–7.09 (m, 3H), 6.94 (d, *J* = 8.2 Hz, 1H), 6.22 (dd, *J* = 6.5, 2.4 Hz, 1H), 4.05 (q, *J* = 7.6 Hz, xH), 3.94 (s, 3H), 3.90 (s, 3H), 3.07 (td, *J* = 15.9, 7.0 Hz, 1H), 2.12 (td, *J* = 15.9, 2.5 Hz, 1H). ^{13}C NMR ($CDCl_3$, 100 MHz) δ 148.8, 148.6, 147.0, 144.6, 142.4, 139.8, 138.7, 132.8, 132.0, 127.8, 127.2, 126.6, 124.4, 122.2, 120.7, 120.5, 119.1, 119.0, 111.4, 111.1, 56.0, 42.4, 26.8. HRMS: Calcd for $C_{24}H_{21}O_2$ (M^++H) 341.1542, Found 341.1556.

General procedure for the synthesis of trialkylated truxenes: *n*BuLi 2.5M in hexanes (3.5 equiv) was added dropwise over a solution of the truxene (1 equiv) in anhydrous THF at −80 °C and the mixture was allowed to warm up to −10 °C for 4 hours. Then, a solution of the alkynylbromide (4.0 equiv) in anhydrous THF was added dropwise and the mixture was allowed to warm to room temperature overnight. Saturated NaCl solution (x mL) was added and the organic layer was extracted with ethyl acetate. The combined organic fractions were dried over anhydrous Na_2SO_4 and

filtered and the solvent was evaporated under reduced pressure. The crude product was purified by flash chromatography (SiO_2, eluent = hexane:CH_2Cl_2).

4,9,14-Trimethoxy-5,10,15-tris(3-phenylprop-2-ynyl)-10,15-dihydro-5*H*-diindeno[1,2-*a*:1',2'-*c*]fluorene (1a).

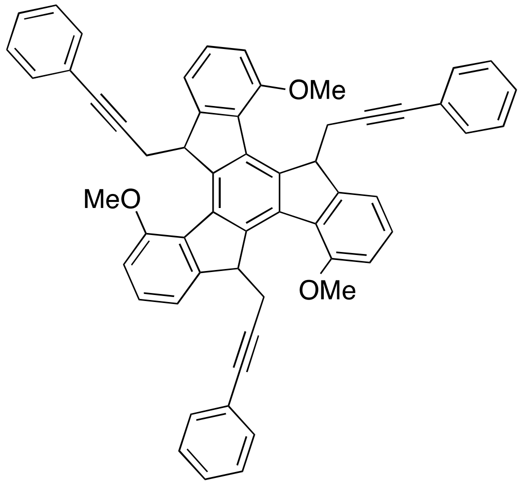

White solid (yield = 57%).

R_f (1:1 hexane:CH_2Cl_2): 0.35; mp =130–131 °C. ^{1}H NMR ($CDCl_3$, 400 MHz) δ 7.82–7.85 (m, 3H), 7.55–7.59 (m, 3H), 7.35–7.43 (m, 6H), 7.21–7.23 (m, 2H), 7.10–7.15 (m, 3H), 7.00 (d, J = 8.2 Hz, 3H), 5.29–5.35 (m, 2H), 5.22–5.25 (m, 1H), 4.20 (s, 6H), 4.17 (s, 3H), 3.39–3.51 (m, 3H), 2.23–2.30 (m, 1H), 2.08–2.17 (m, 2H). ^{13}C NMR ($CDCl_3$, 100 MHz, DEPT) δ 154.2 (C), 154.2 (C), 154.1 (C), 151.2 (C), 151.0 (C), 150.4 (C), 141.2 (C), 139.8 (C), 139.8 (C), 136.5 (C), 136.1 (C), 136.0 (C), 133.5 (CH), 133.4 (CH), 132.4 (CH), 132.3 (CH), 128.7 (CH), 128.7 (CH), 128.6 (CH), 128.5 (CH), 128.3 (CH), 128.0 (C), 127.6 (C), 127.5 (C), 126.9 (CH), 126.9 (CH), 126.8 (CH), 126.2 (C), 126.1 (C), 125.2 (C), 125.1 (C), 117.8 (CH), 117.7 (CH), 117.5 (CH), 109.6 (CH), 95.2 (C), 95.2 (C), 95.1 (C), 80.4 (C), 80.3 (C), 80.2 (C), 55.5 (CH_3), 55.4 (CH_3), 49.3 (CH), 48.8 (CH), 48.7 (CH), 27.6, (CH_2), 27.4 (CH_2), 26.3 (CH_2). Elemental analysis calcd (%) for $C_{57}H_{42}O_3 \cdot H_2O$: C 86.34, H 5.59; found: C 86.60, H 5.72.

5,10,15-Tris(3-(2-bromophenyl)prop-2-ynyl)-4,9,14-trimethoxy-10,15-dihydro-5*H*-diindeno[1,2-*a*:1',2'-*c*]fluorene (1b).

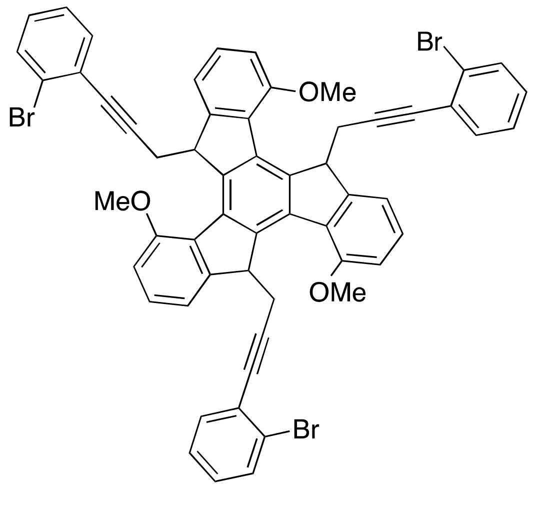

White solid (yield = 54%).

R_f (1:1 hexane:CH_2Cl_2): 0.35; mp = 144–145 °C. ^{1}H NMR ($CDCl_3$, 400 MHz) δ 7.82–7.85 (m, 3H), 7.55–7.59 (m, 3H), 7.35–7.43 (m, 6H), 7.21–7.23 (m, 2H), 7.10–7.15 (m, 3H), 7.00 (d, *J* = 8.2 Hz, 3H), 5.29–5.35 (m, 2H), 5.22–5.25 (m, 1H), 4.20 (s, 6H), 4.17 (s, 3H), 3.39–3.51 (m, 3H), 2.23–2.30 (m, 1H), 2.08–2.17 (m, 2H). ^{13}C NMR ($CDCl_3$, 100 MHz, DEPT) δ 154.3 (C), 153.2 (C), 153.1 (C), 151.2 (C), 151.0 (C), 150.4 (C), 141.2 (C), 139.8 (C), 139.7 (C), 136.5 (C), 136.1 (C), 136.0 (C), 133.5 (CH), 133.4 (CH), 132.3 (CH), 128.7 (CH), 128.6 (CH), 128.5 (CH), 128.4 (CH), 128.0 (C), 127.6 (C), 127.5 (C), 126.9 (CH), 126.2 (C), 126.1 (C), 125.2 (C), 125.1 (C), 117.8 (CH), 117.7 (CH), 117.5 (CH), 109.5 (CH), 95.2 (C), 95.1 (C), 95.0 (C), 80.4 (C), 80.3 (C), 80.2 (C), 55.5 (CH_3), 55.4 (CH_3), 49.3 (CH), 48.8 (CH), 48.7 (CH), 27.6 (CH_2), 27.4 (CH_2), 26.3 (CH_2). Elemental analysis calcd (%) for $C_{57}H_{39}Br_3O_3$: C 67.67, H 3.89; found: C 67.30, H 4.00.

1,7,13-Trimethoxy-4,10,16-triphenyldiacenaphtho[1,2-*j*:1',2'-*l*]fluoranthene (2a).

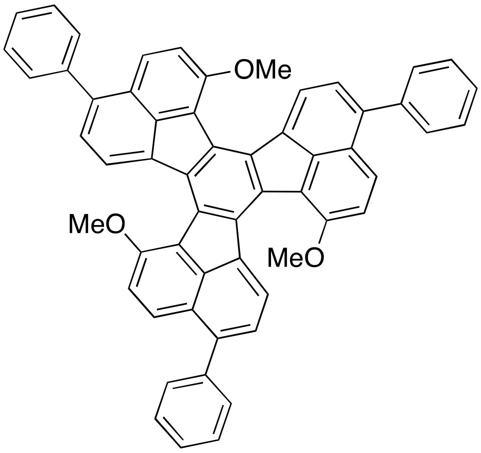

Yellow solid (yield = 72%).

mp = > 300 °C. ^{1}H NMR ($CDCl_2CDCl_2$, 500 MHz, 403 K) δ 8.24 (d, *J* = 7.4 Hz, 3H), 8.01 (d, *J* = 9.0 Hz, 3H), 7.66 (d, *J* = 7.0 Hz, 6H), 7.60 (d, *J* = 7.4 Hz, 3H), 7.51 (t, *J* = 7.4 Hz, 6H), 7.43 (d, *J* = 9.0 Hz, 6H), 4.04 (s, 9H). MS (MALDI): 768.5 (M^+).

4,10,16-Tris(2-bromophenyl)-1,7,13-trimethoxydiacenaphtho[1,2-*j*:1',2'-*l*]fluoranthene (2b).

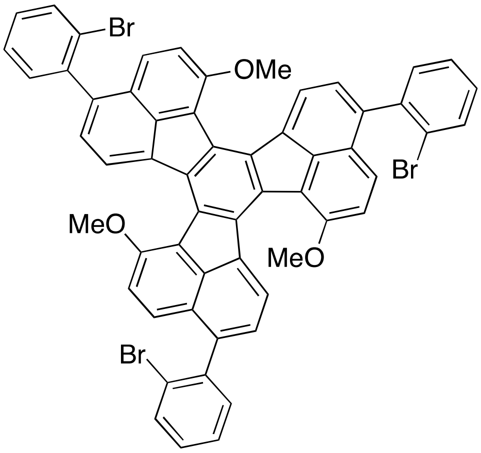

Yellow solid (yield = 77%).

mp = > 300 °C. ^{1}H NMR ($CDCl_2CDCl_2$, 500 MHz, 403 K) δ 8.27 (d, *J* = 7.4 Hz, 3H), 7.78 (d, *J* = 8.0 Hz, 3H), 7.62 (d, *J* = 8.9 Hz, 3H), 7.53 (t, *J* = 7.2 Hz, 6H), 7.44 (t, *J* = 7.3 Hz, 3H), 7.39 (d, *J* = 8.9 Hz, 3H), 7.31 (t, *J* = 7.4 Hz, 3H), 4.04 (s, 9H). MS (MALDI): 1004.1, 1006.1 (M^+).

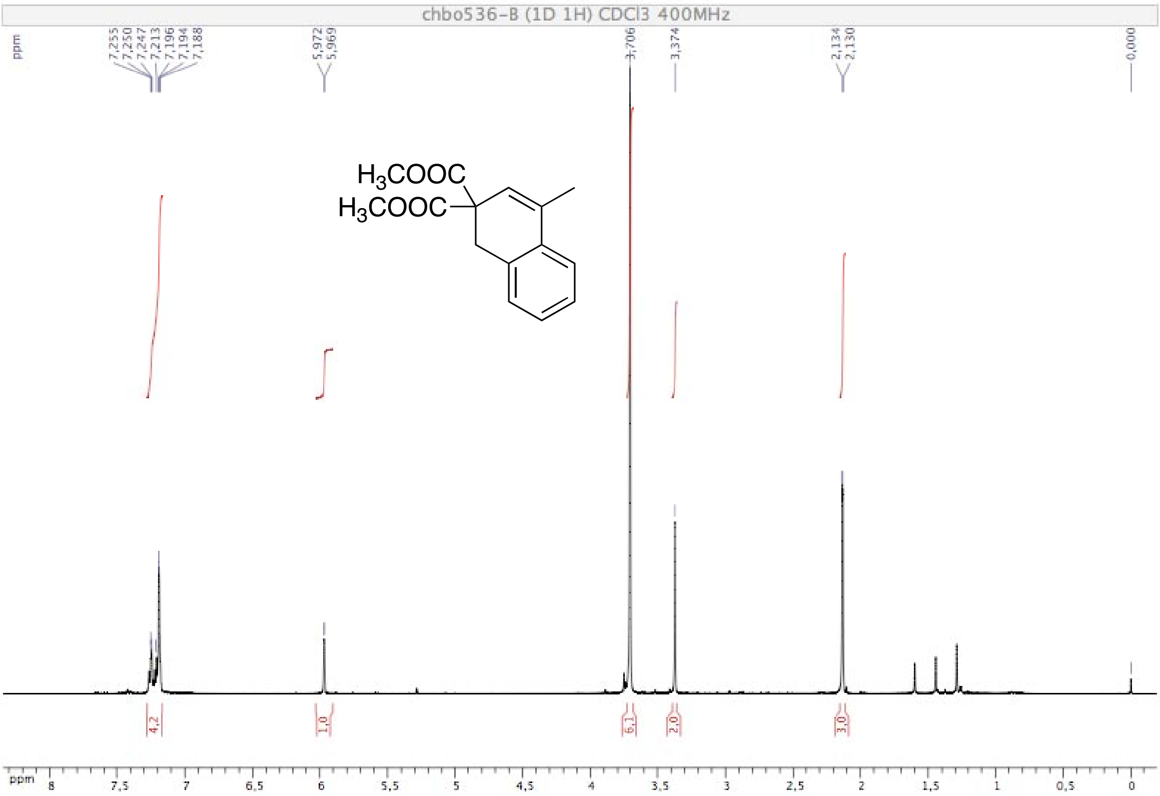
chbo536-B (1D 1H) CDCl3 400MHz
7,255 7,250 7,247 7,213 7,196 7,194 7,188
5,972 5,969
3,706
3,374
2,134 2,130
0,000
H_3COOC
H_3COOC
4,2
1,0
6,1
2,0
3,0
ppm

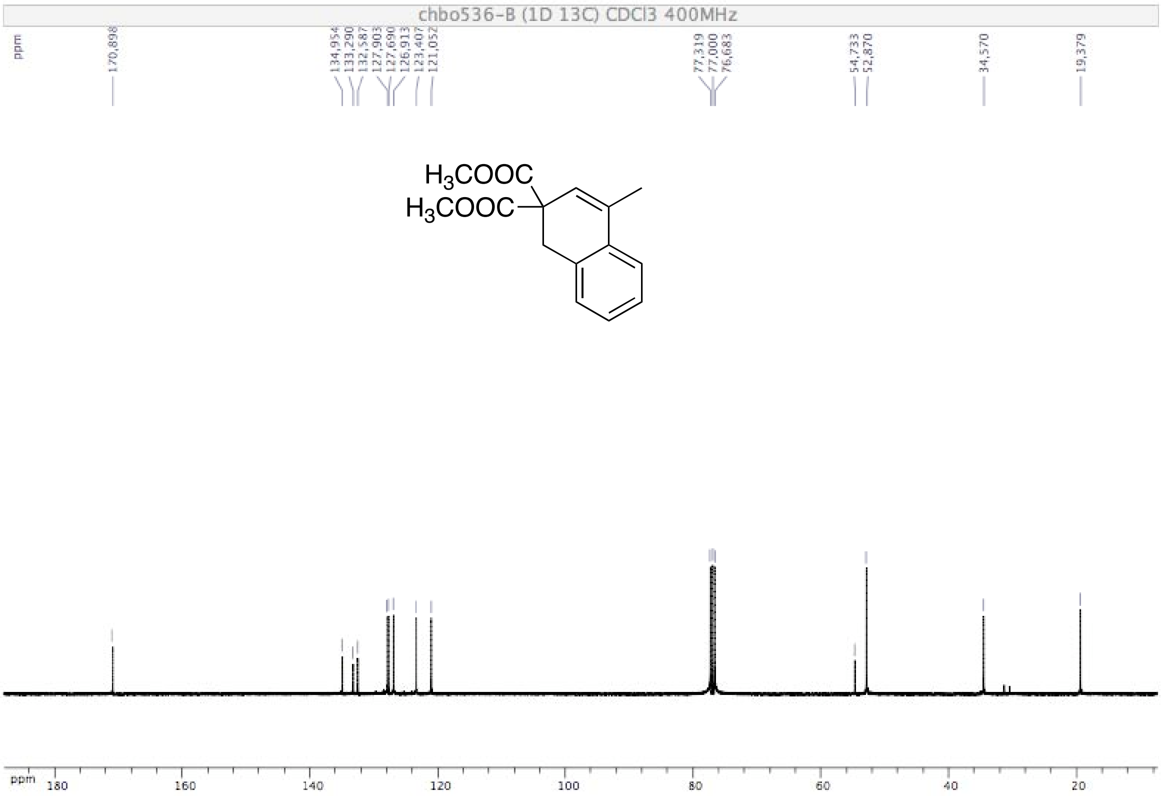
chbo536-B (1D 13C) CDCl3 400MHz
170,898
134,954 133,290 132,587 127,903 127,690 126,913 123,407 121,052
77,319 77,000 76,683
54,733 52,870
34,570
19,379
H_3COOC
H_3COOC
ppm

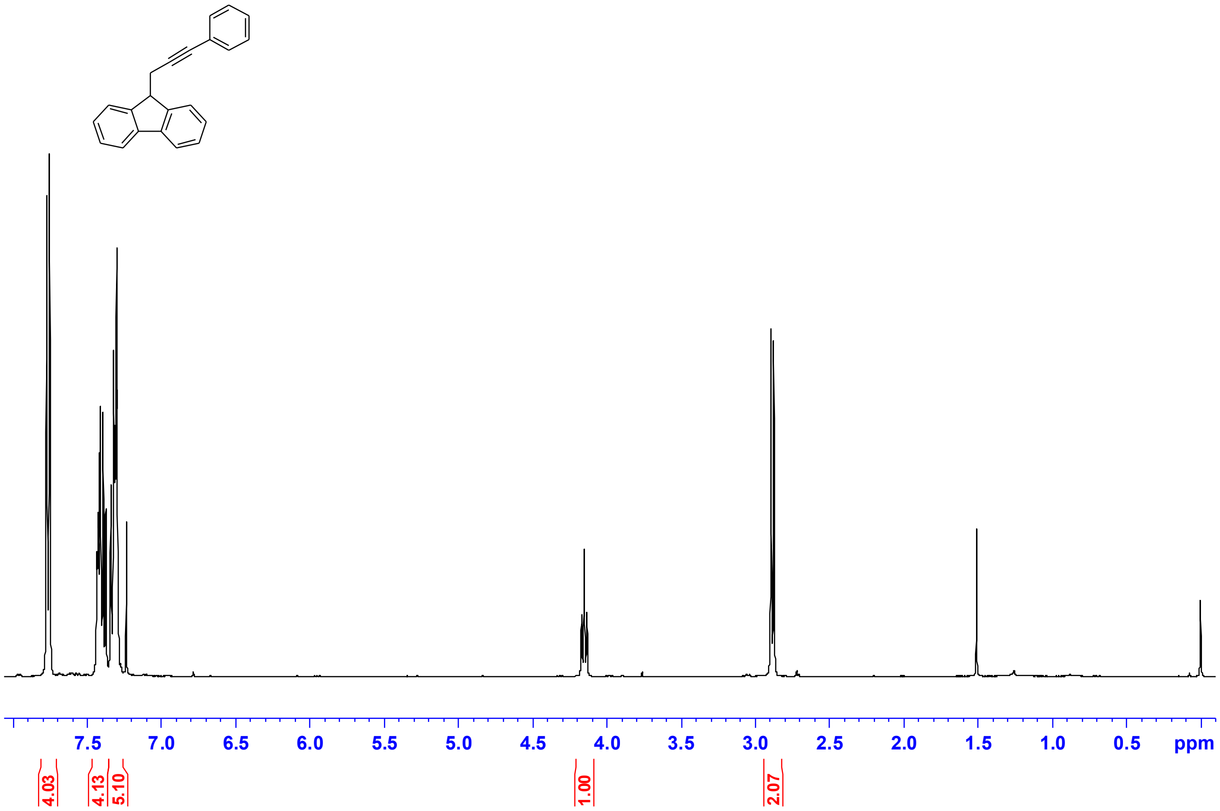
7.5
7.0
6.5
6.0
5.5
5.0
4.5
4.0
3.5
3.0
2.5
2.0
1.5
1.0
0.5
ppm
4.03
4.13
5.10
1.00
2.07

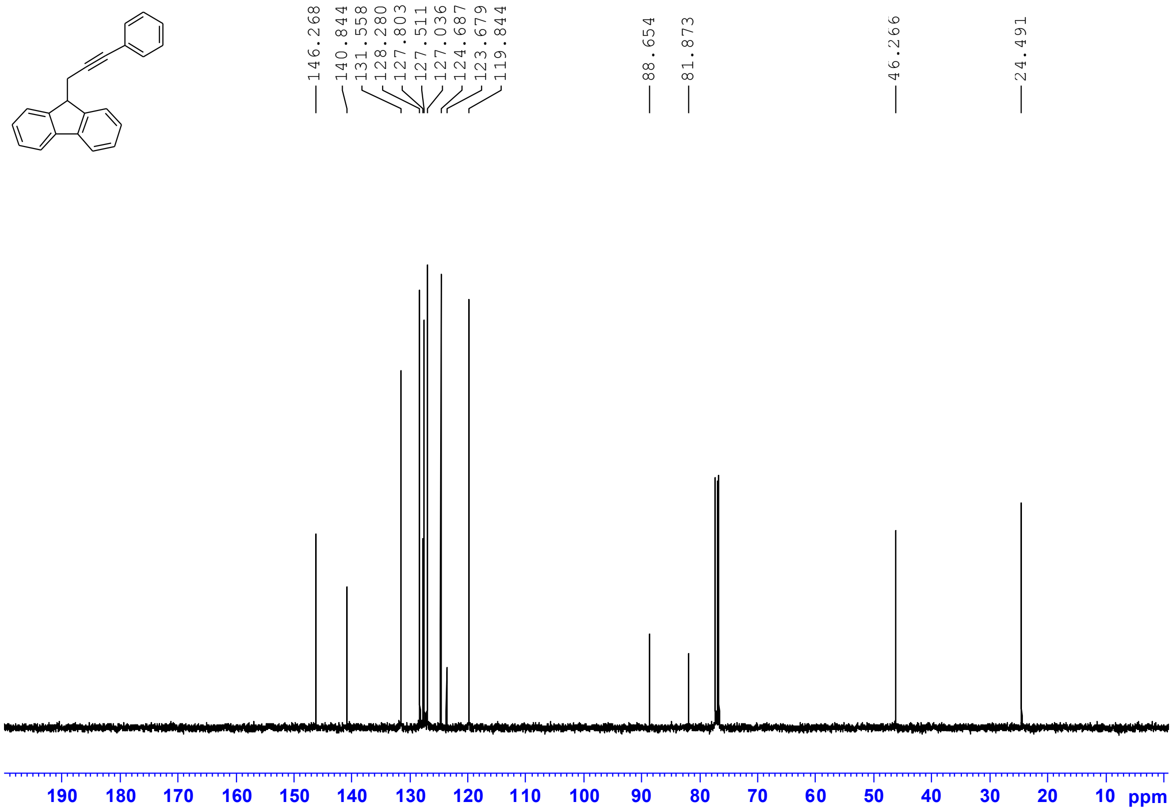
146.268
140.844
131.558
128.280
127.803
127.511
127.036
124.687
123.679
119.844
88.654
81.873
46.266
24.491
190
180
170
160
150
140
130
120
110
100
90
80
70
60
50
40
30
20
10
ppm

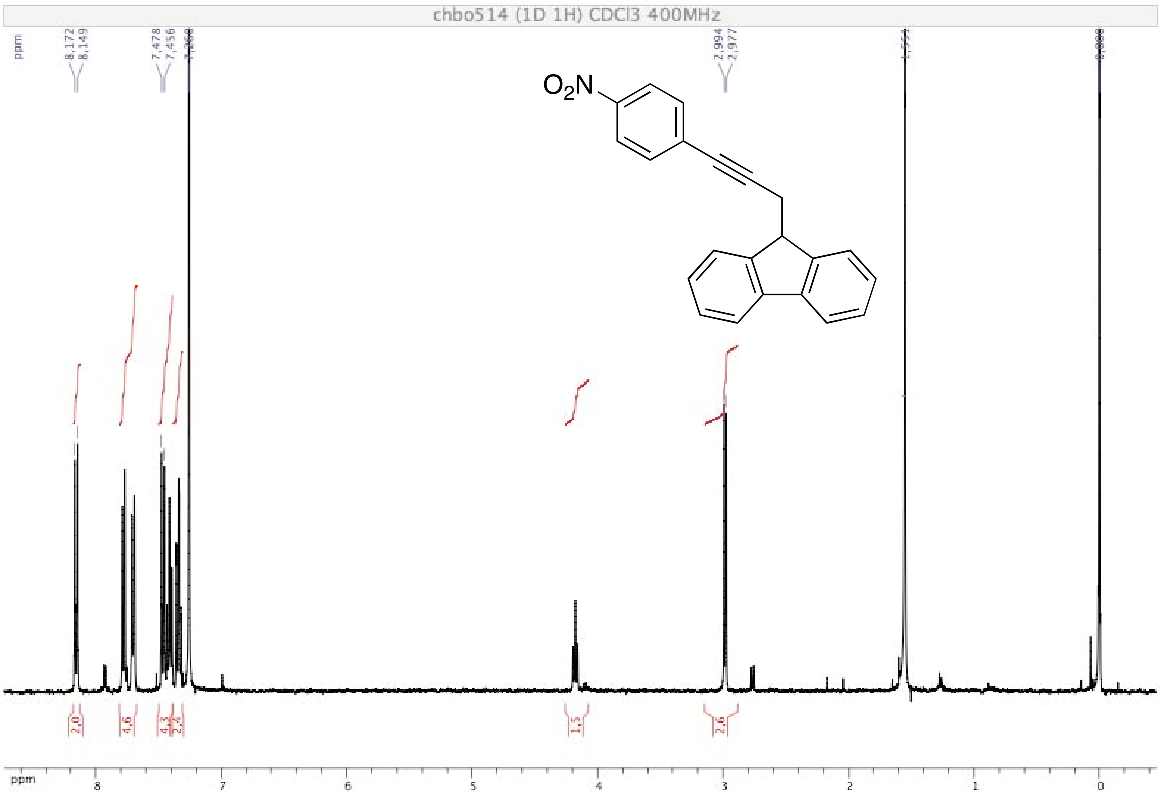
chbo514 (1D 1H) CDCl3 400MHz
O_2N
ppm
2,0
4,6
4,3
2,4
1,5
2,6

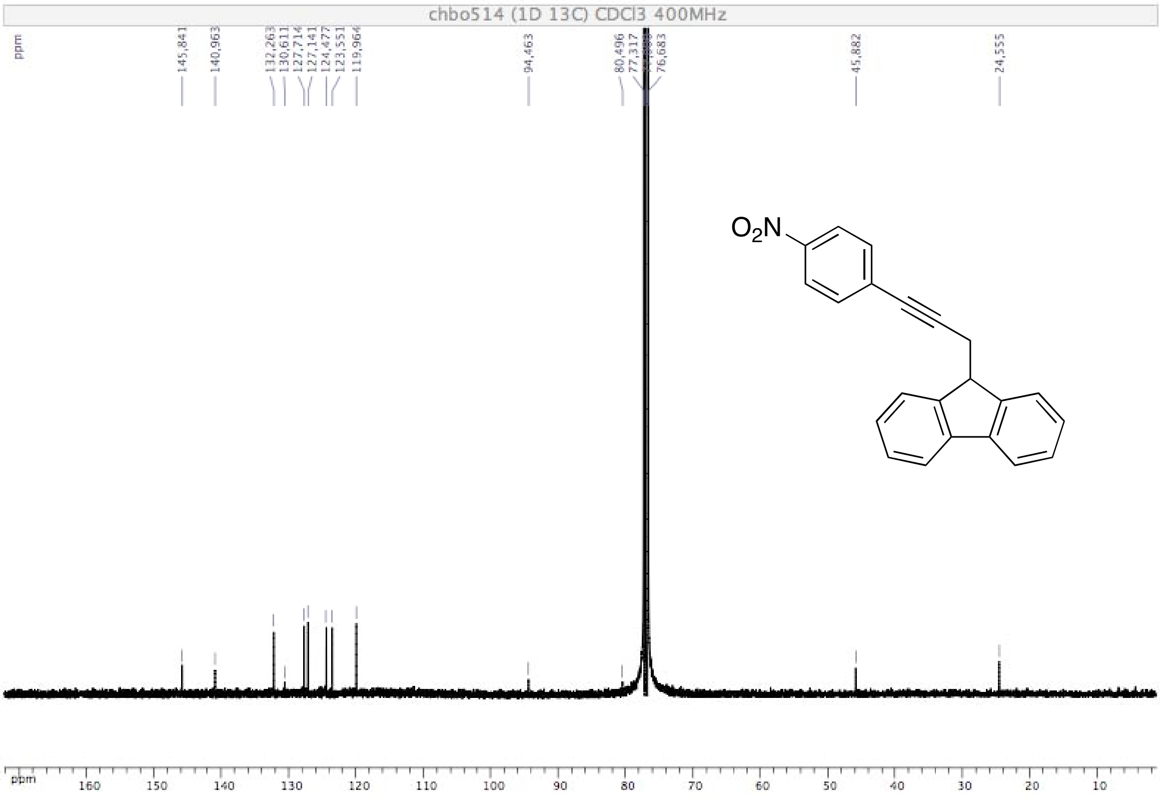
chbo514 (1D 13C) CDCl3 400MHz
145,841
140,963
132,263
130,611
127,714
127,141
124,477
123,551
119,964
94,463
80,496
77,317
76,683
45,882
24,555
O_2N
ppm

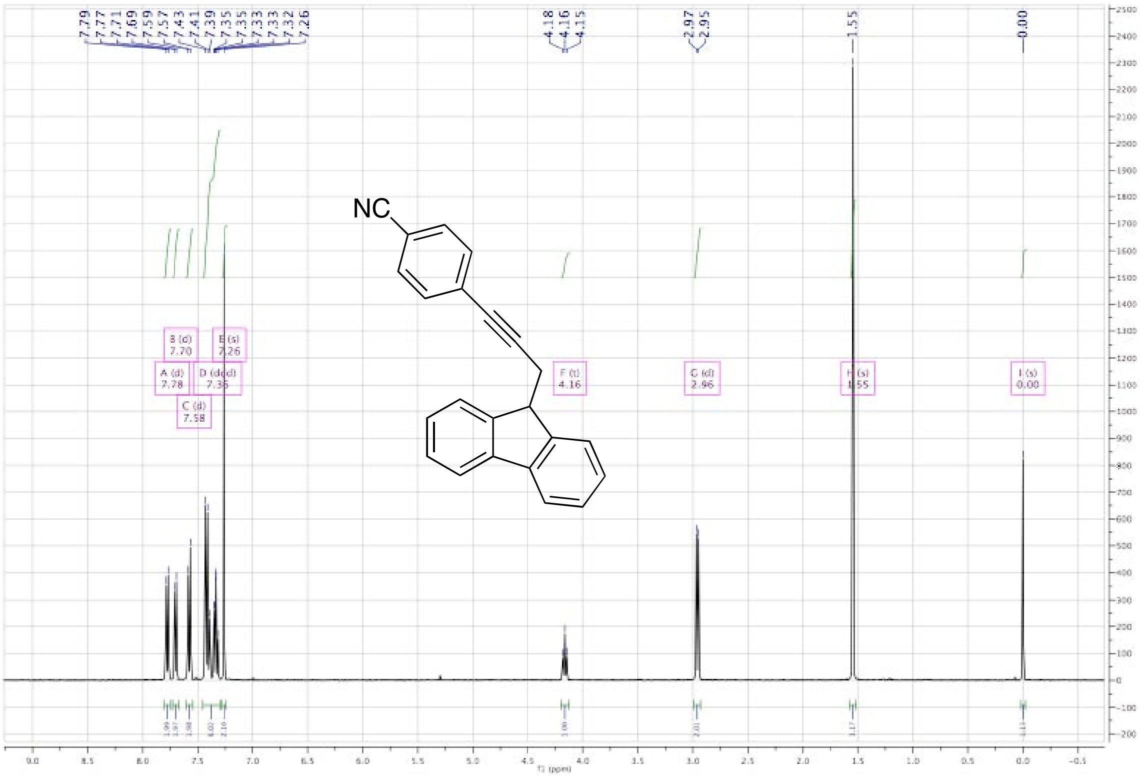
NC
f1 (ppm)

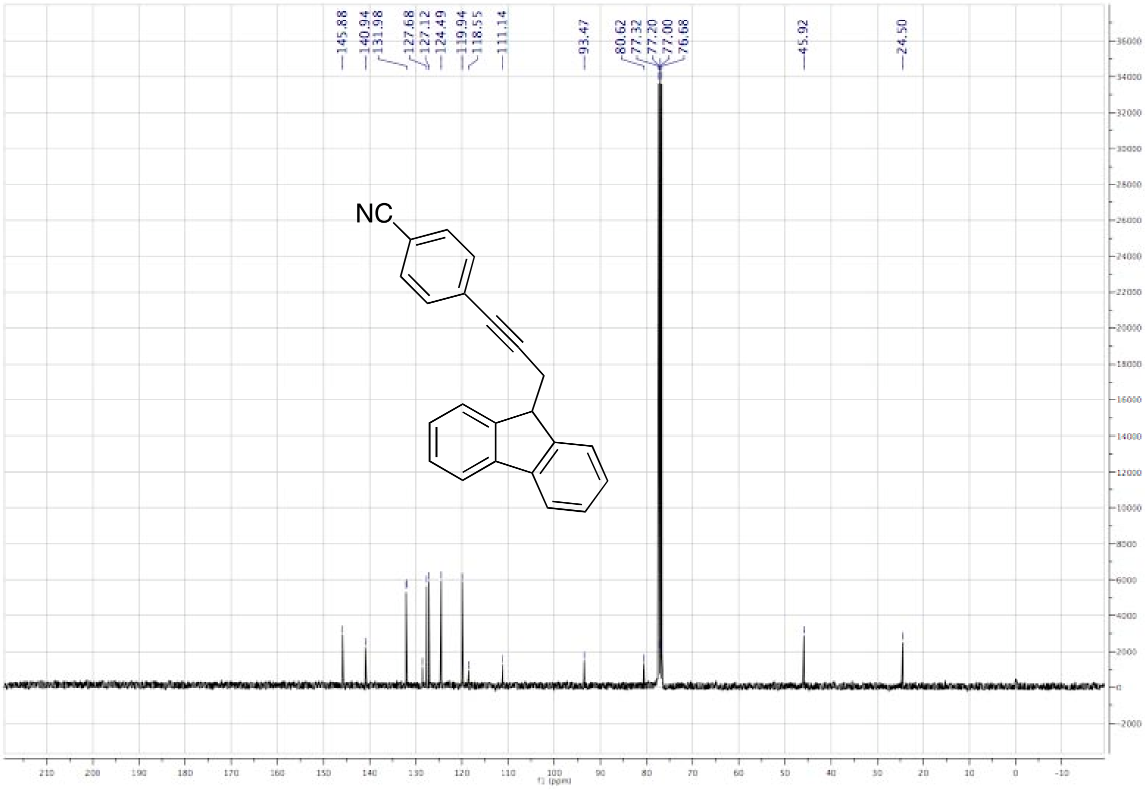
NC
f1 (ppm)

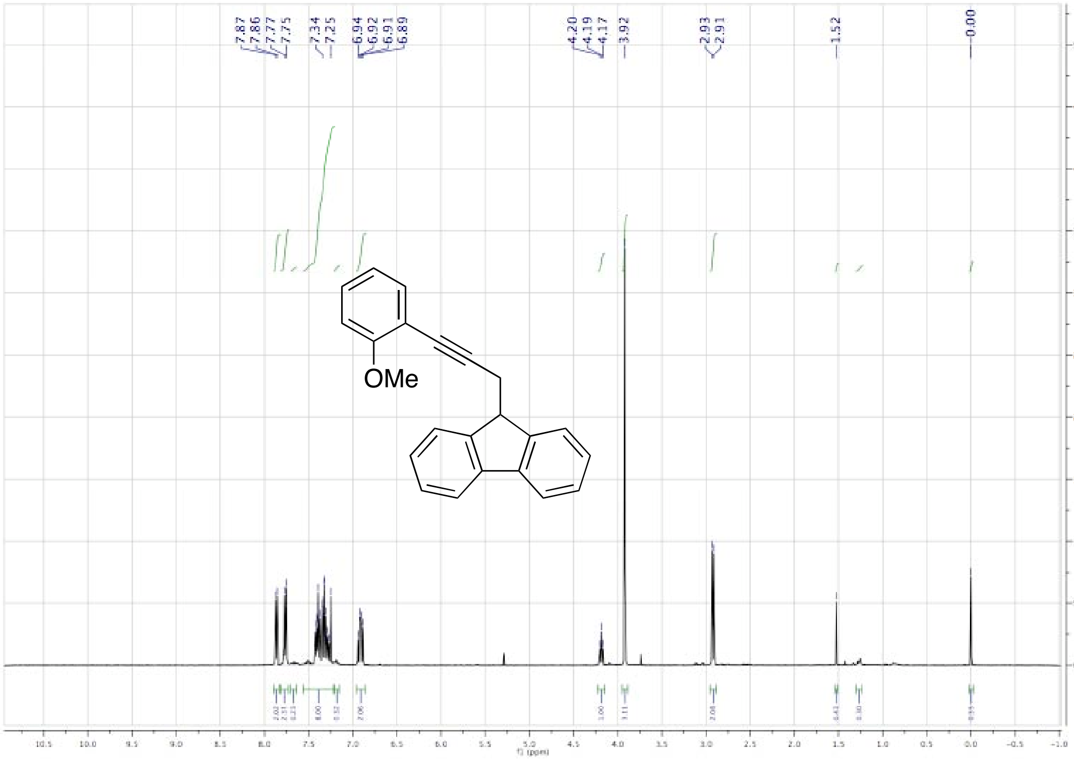
7.87
7.86
7.77
7.75
7.34
7.25
6.94
6.92
6.91
6.89
4.20
4.19
4.17
3.92
2.93
2.91
1.52
0.00
OMe
f1 (ppm)

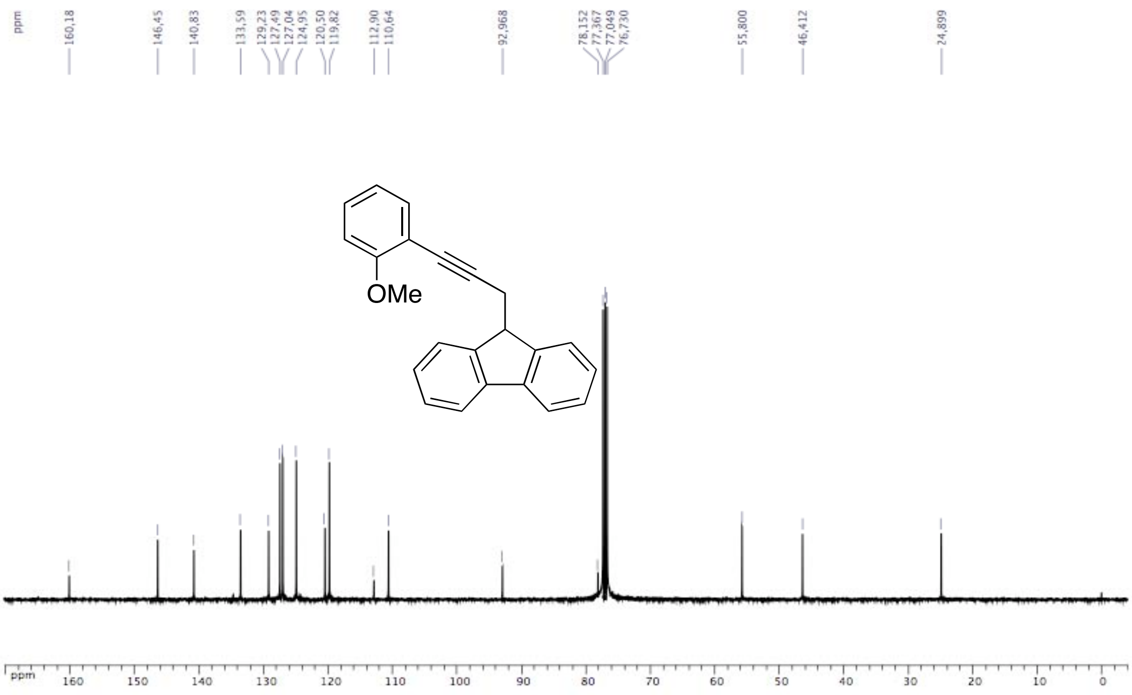

OMe
ppm
160,18
146,45
140,83
133,59
129,23
127,49
127,04
124,95
120,50
119,82
112,90
110,64
92,968
78,152
77,367
77,049
76,730
55,800
46,412
24,899

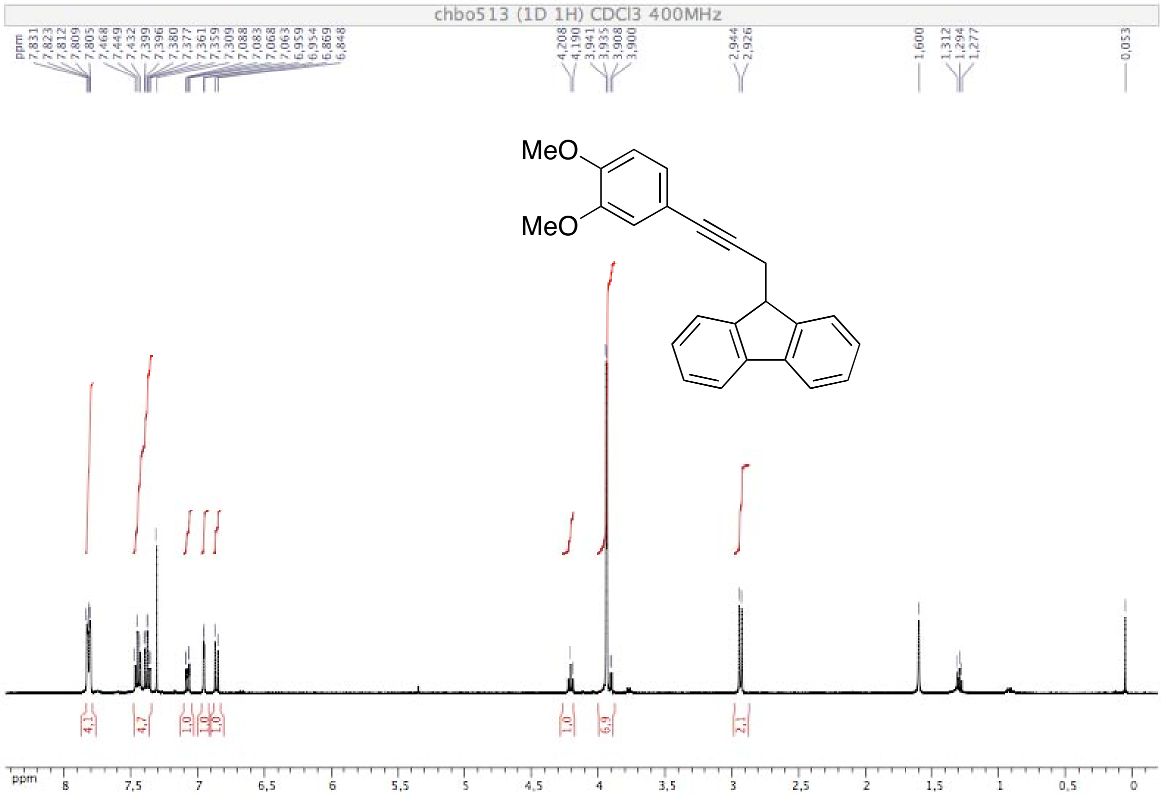

chbo513 (1D 1H) CDCl3 400MHz
MeO
MeO
4,1
4,7
1,0
1,0
1,0
1,0
6,9
2,1

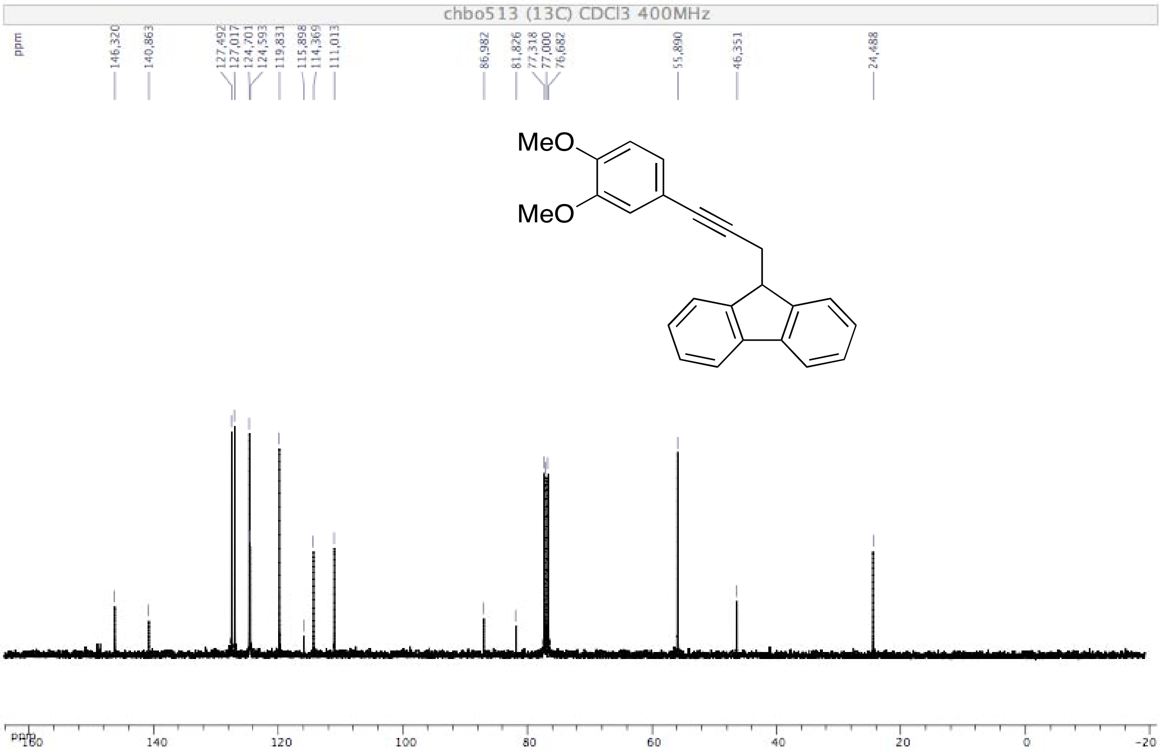
chbo513 (13C) CDCl3 400MHz
ppm
146.320
140.863
127.492
127.017
124.701
124.593
119.831
115.898
114.369
111.013
86.982
81.826
77.318
77.000
76.682
55.890
46.351
24.488
MeO
MeO
160
140
120
100
80
60
40
20
0
-20

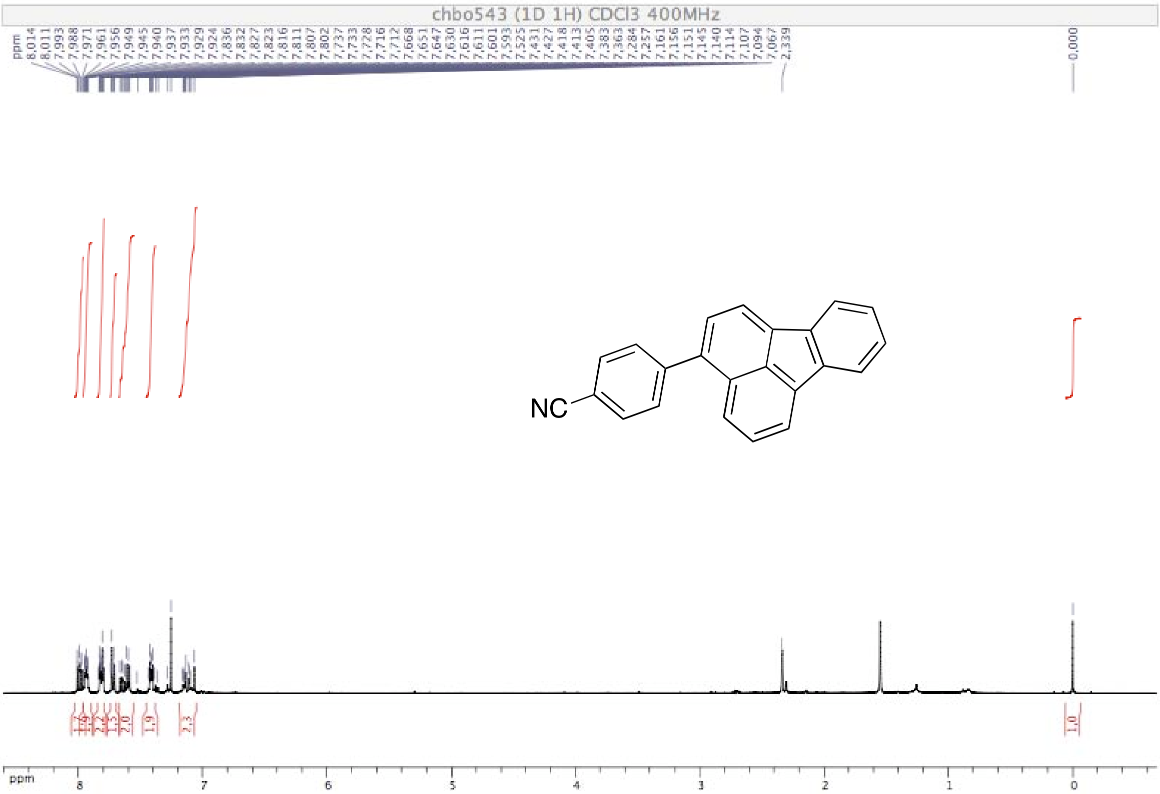
chbo543 (1D 1H) CDCl3 400MHz
ppm
8.014 8.011 7.993 7.988 7.971 7.961 7.956 7.949 7.945 7.940 7.937 7.933 7.929 7.924 7.836 7.832 7.827 7.823 7.816 7.811 7.807 7.802 7.737 7.733 7.728 7.716 7.712 7.668 7.651 7.647 7.630 7.616 7.611 7.601 7.593 7.525 7.431 7.427 7.418 7.413 7.405 7.383 7.363 7.284 7.257 7.161 7.156 7.151 7.145 7.140 7.114 7.107 7.094 7.067 2.339
0.000
NC
1.9
2.3
1.0
ppm 8 7 6 5 4 3 2 1 0

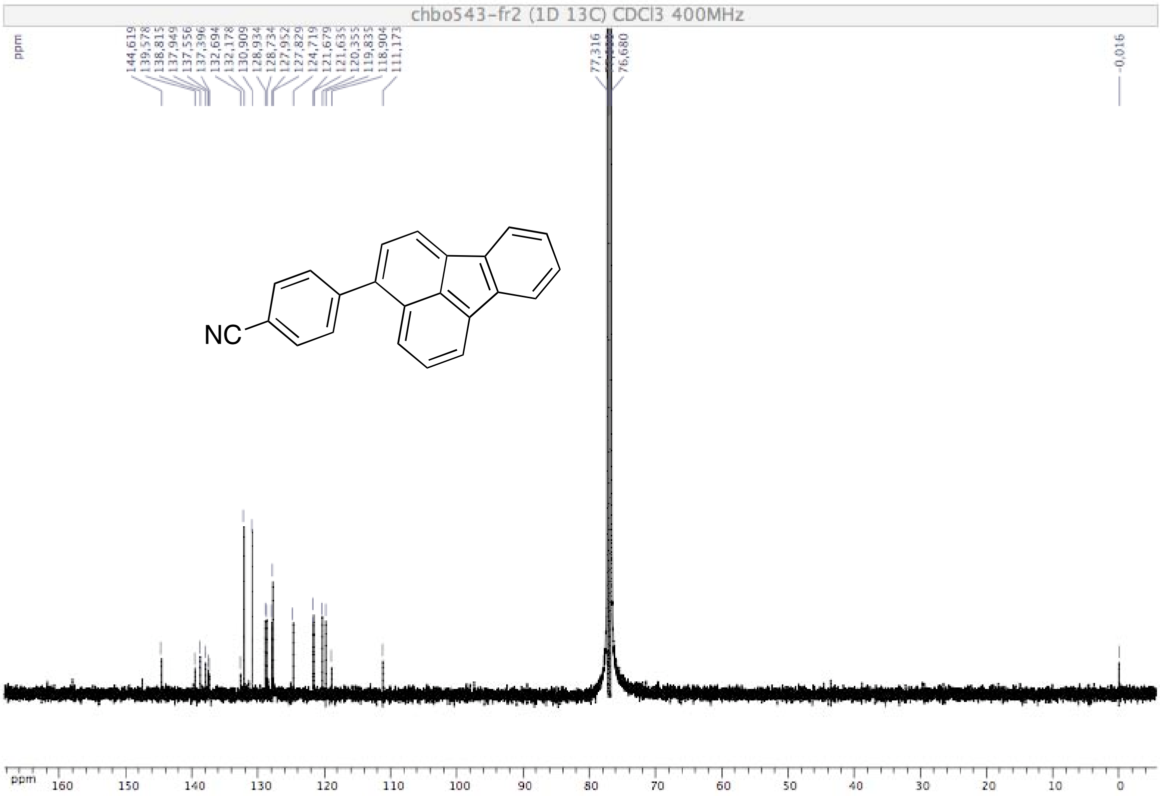
chbo543-fr2 (1D 13C) CDCl3 400MHz
ppm
144.619 139.578 138.815 137.949 137.556 137.396 132.694 132.178 130.909 128.934 128.734 127.952 127.829 124.719 121.679 121.639 120.359 119.835 118.904 111.173
77.316 76.680
-0.016
NC
ppm 160 150 140 130 120 110 100 90 80 70 60 50 40 30 20 10 0

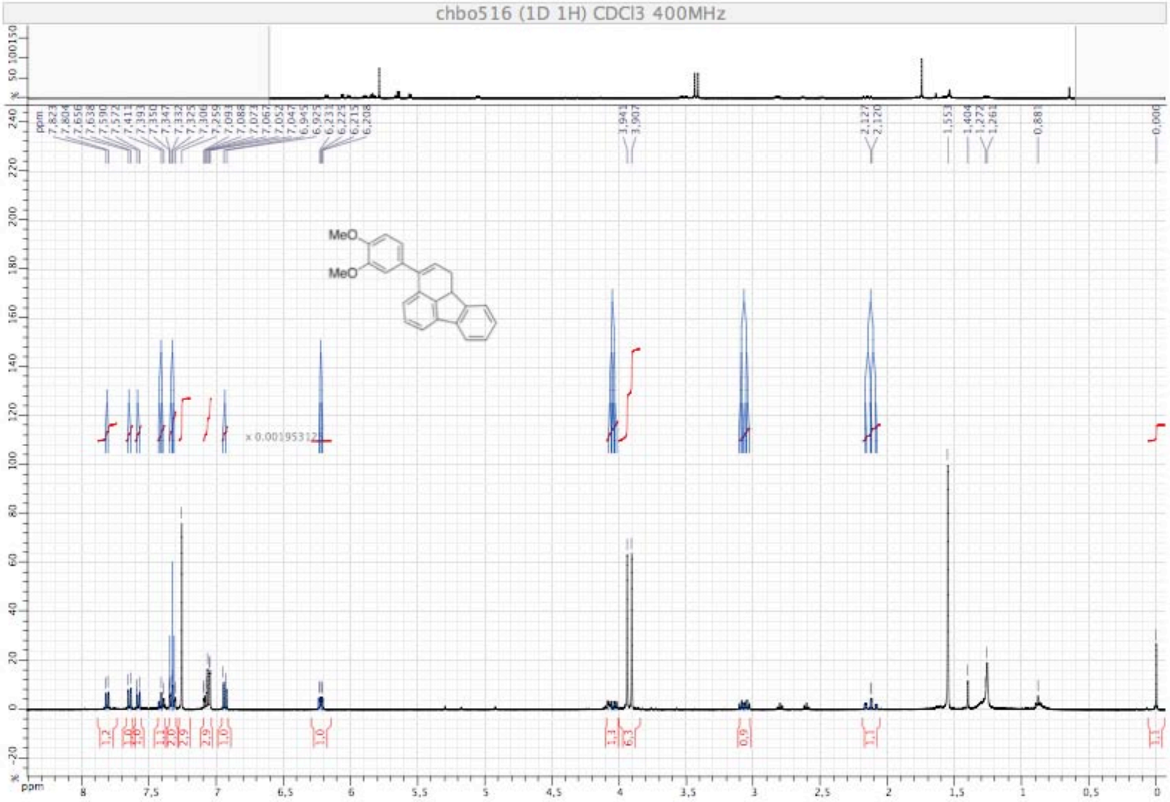
chbo516 (1D 1H) CDCl3 400MHz
MeO
MeO

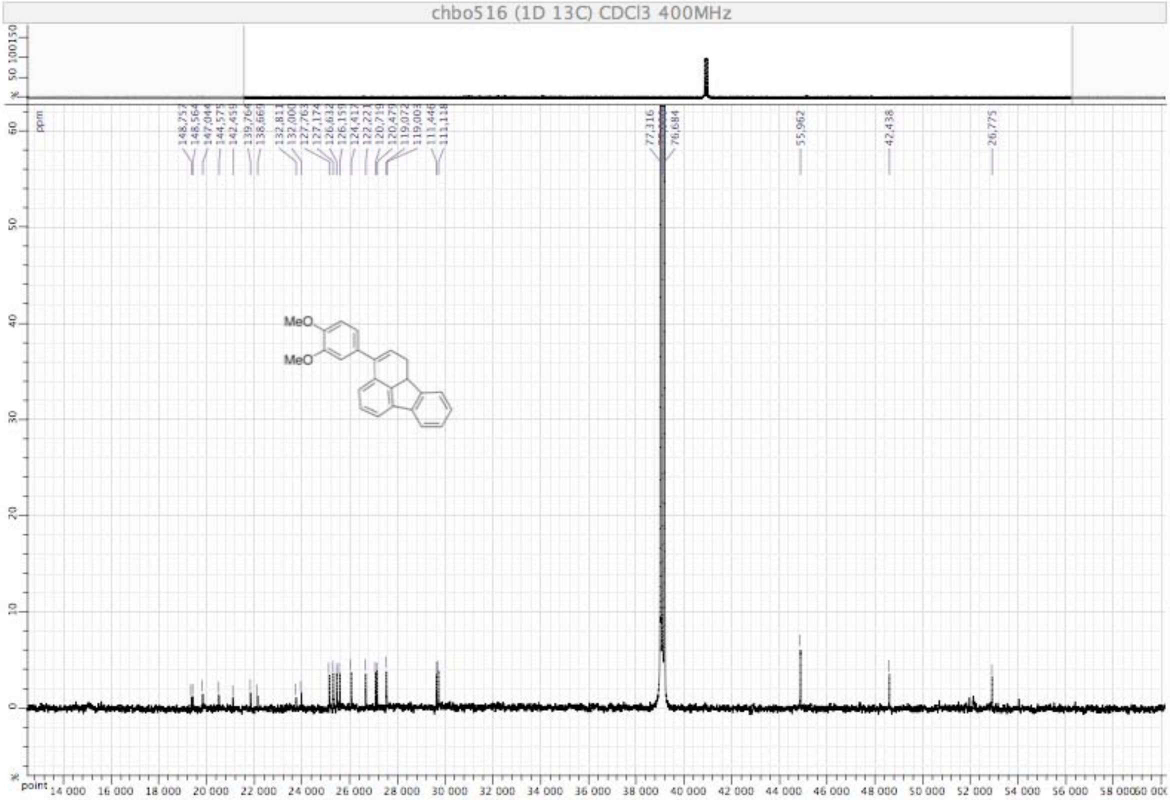
chbo516 (1D 13C) CDCl3 400MHz
MeO
MeO

1a

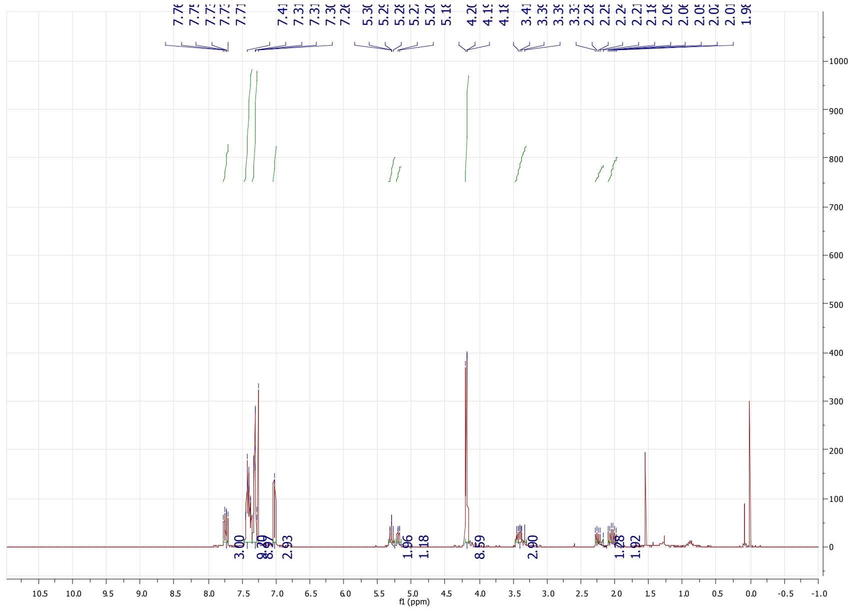

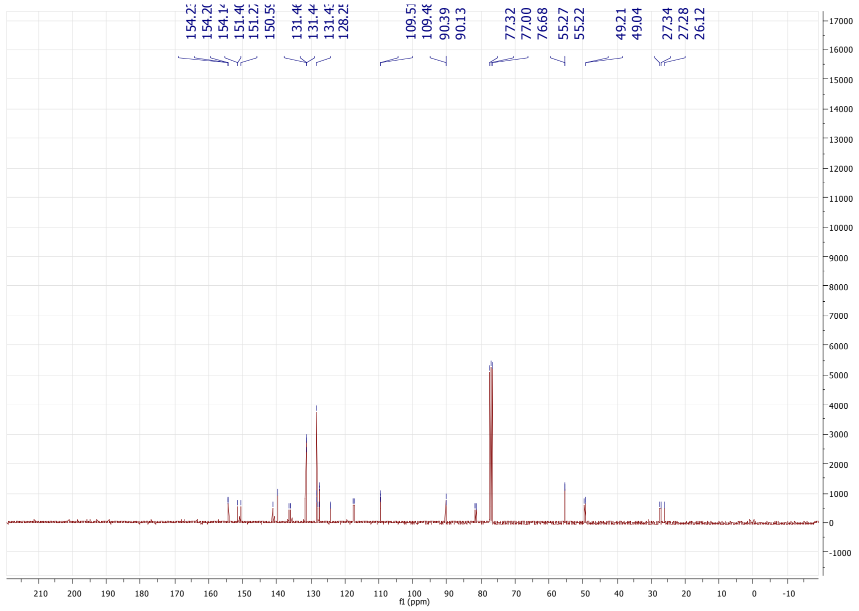

1b

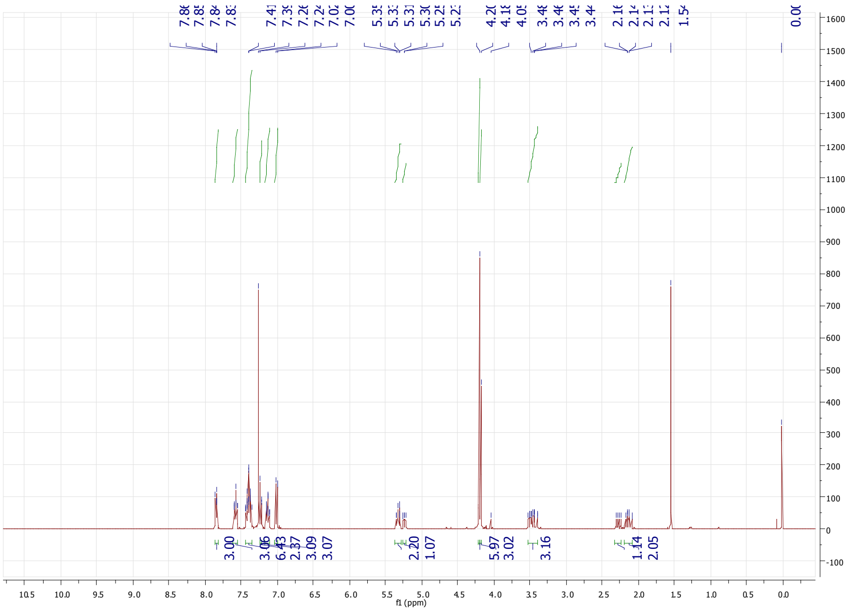

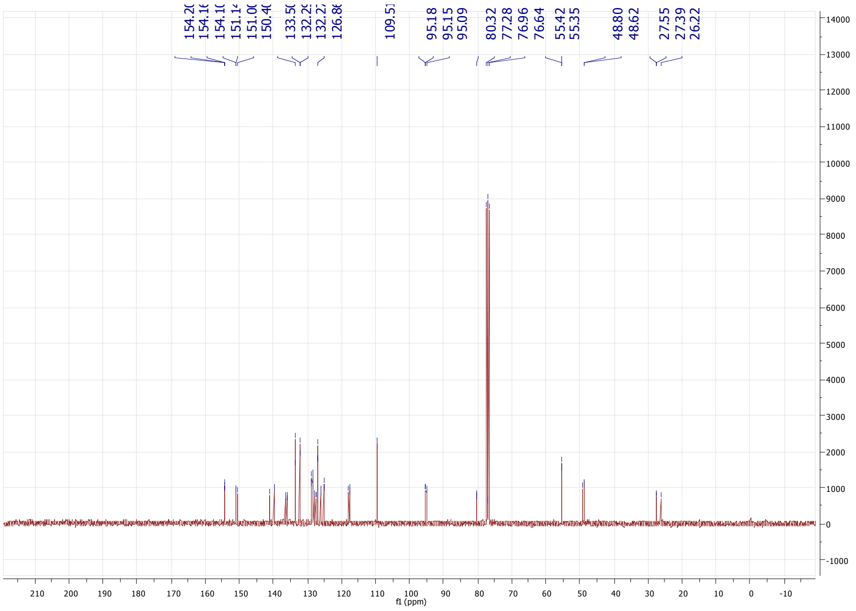

2a

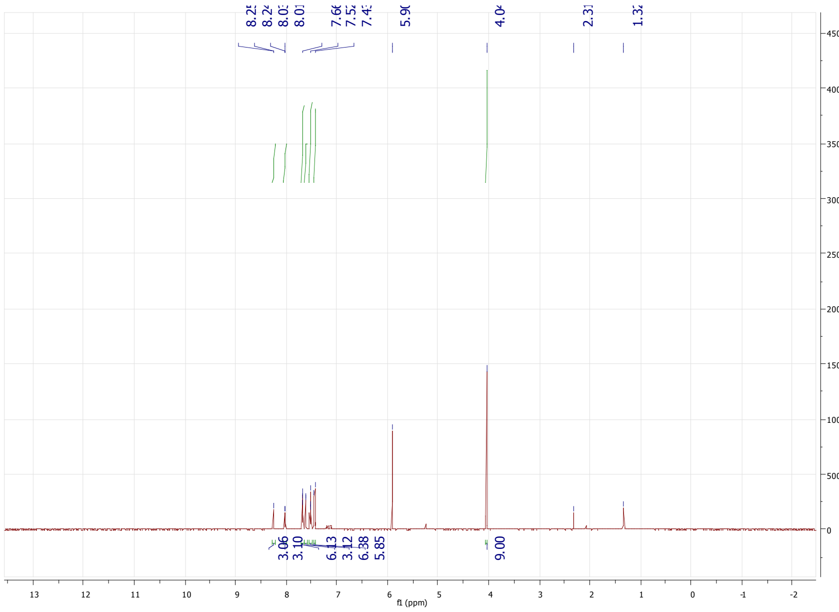

2b

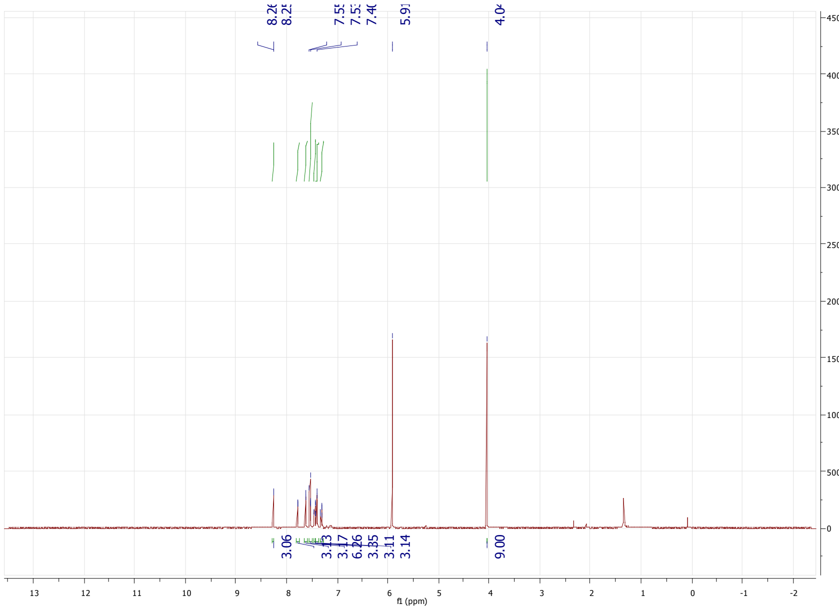

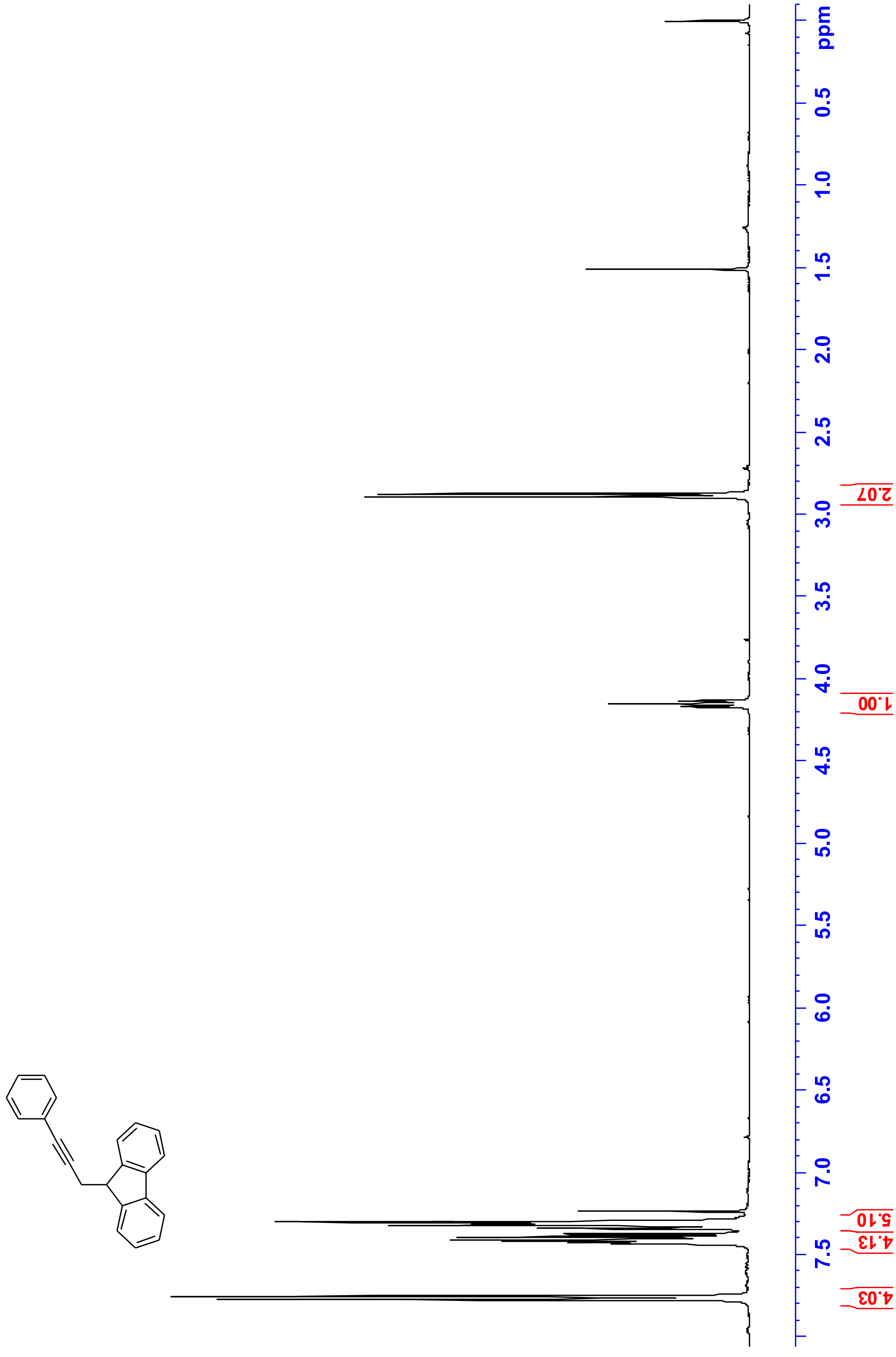
ppm
0.5
1.0
1.5
2.0
2.5
3.0
3.5
4.0
4.5
5.0
5.5
6.0
6.5
7.0
7.5
2.07
1.00
5.10
4.13
4.03

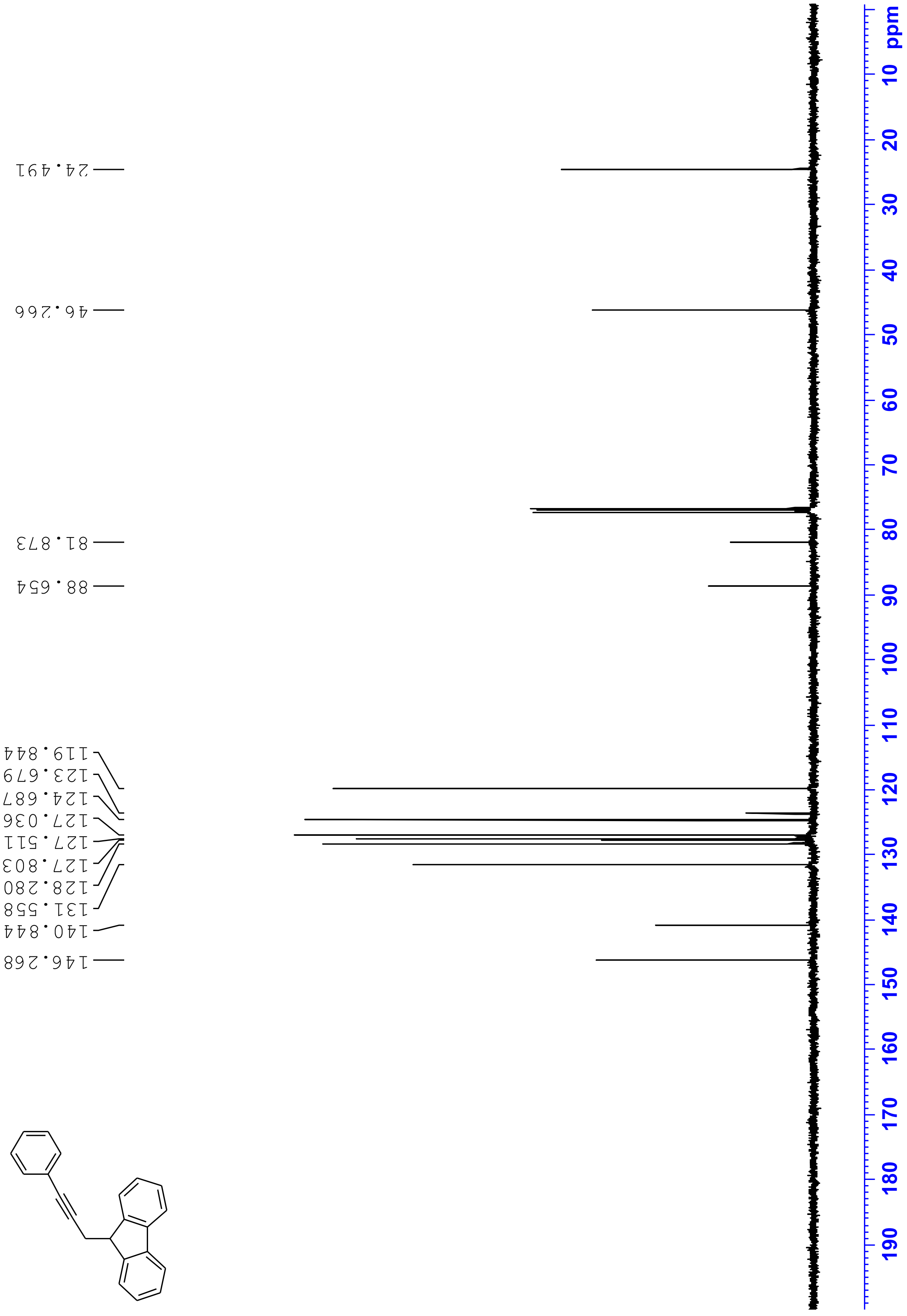
24.491
46.266
81.873
88.654
119.844
123.679
124.687
127.036
127.511
127.803
128.280
131.558
140.844
146.268
ppm
10
20
30
40
50
60
70
80
90
100
110
120
130
140
150
160
170
180
190

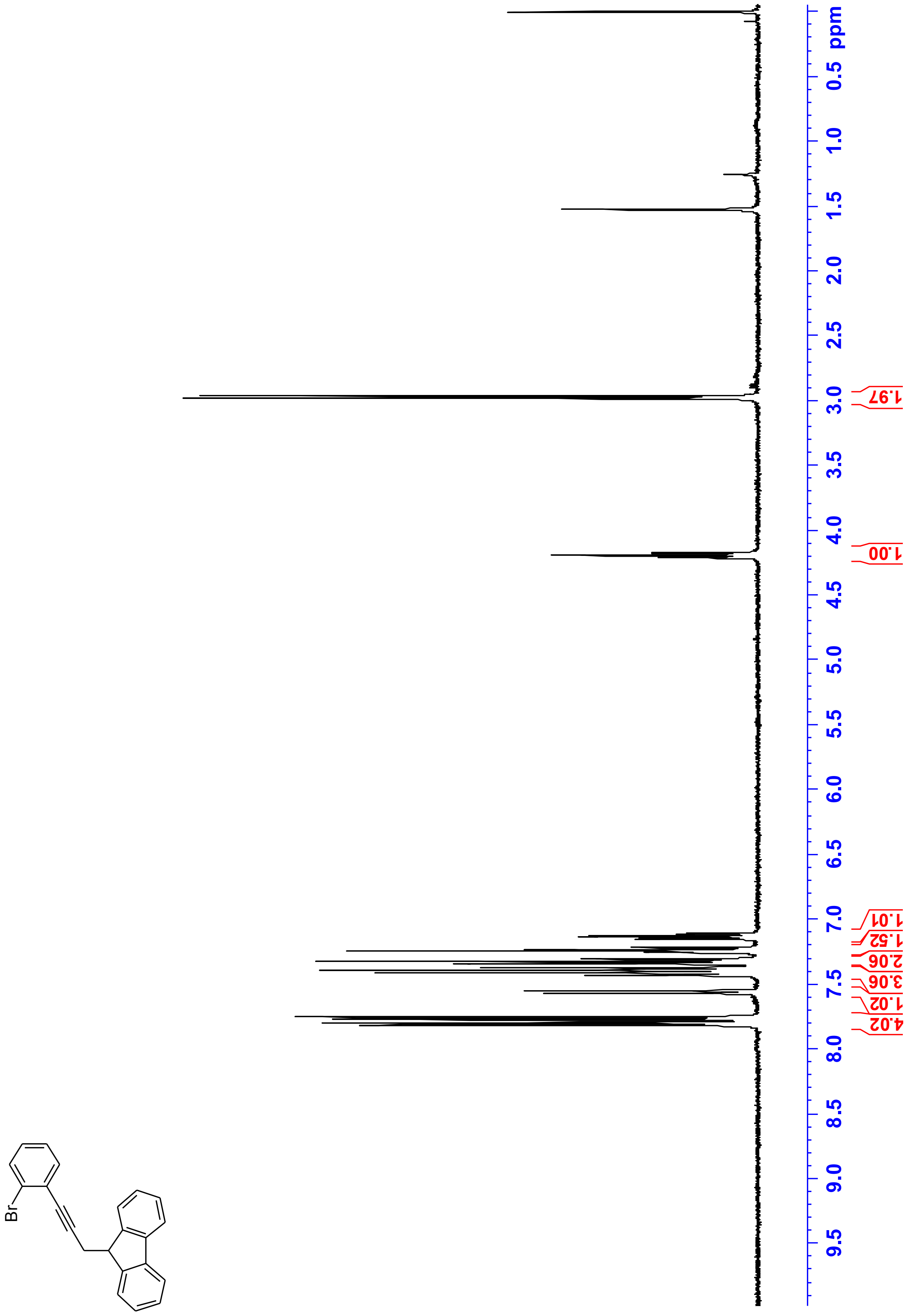

Br
ppm
0.5
1.0
1.5
2.0
2.5
3.0
3.5
4.0
4.5
5.0
5.5
6.0
6.5
7.0
7.5
8.0
8.5
9.0
9.5
1.97
1.00
1.01
1.52
2.06
3.06
1.02
4.02

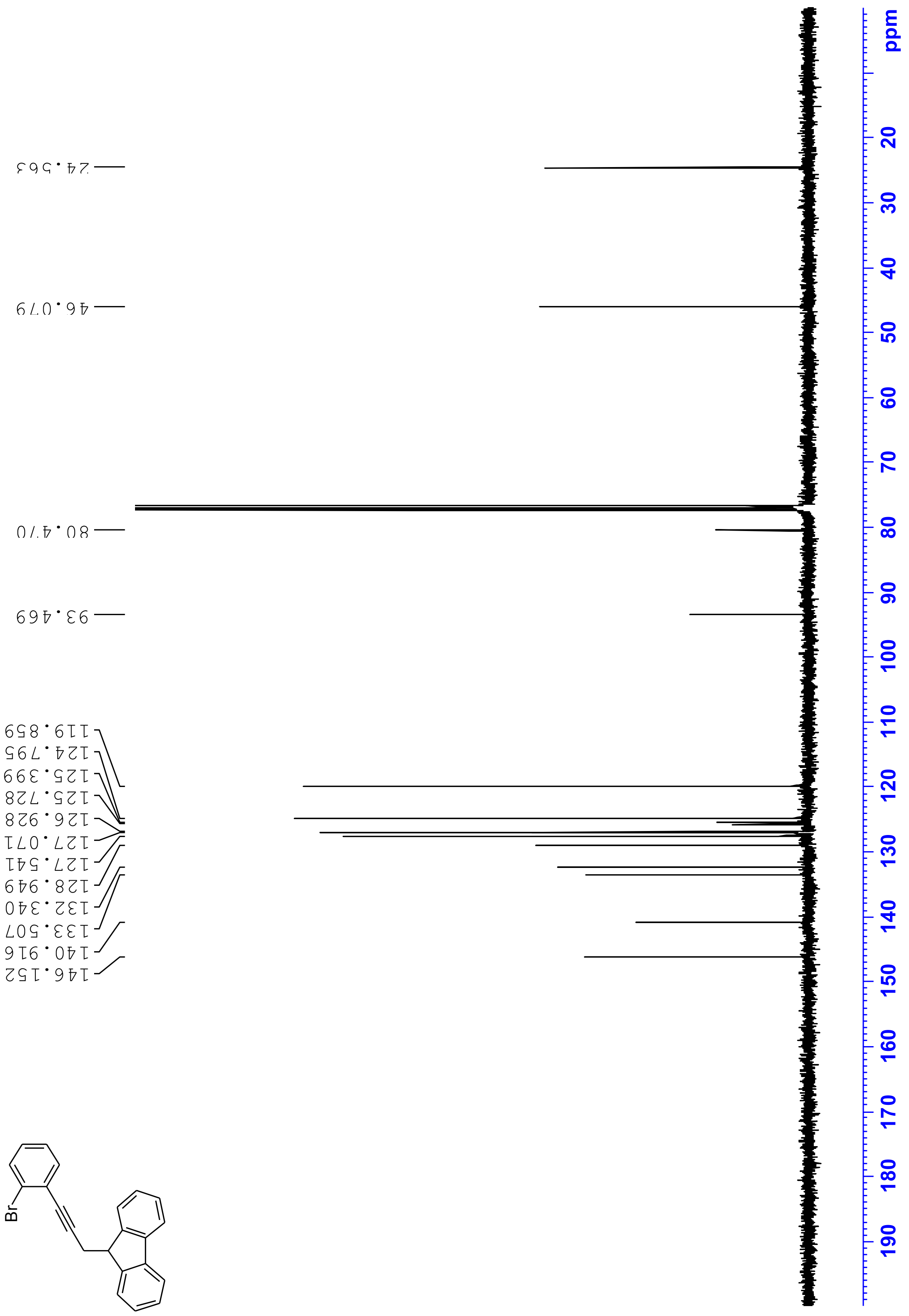
24.563
46.079
80.470
93.469
119.859
124.795
125.399
125.728
126.928
127.071
127.541
128.949
132.340
133.507
140.916
146.152
Br
ppm
20
30
40
50
60
70
80
90
100
110
120
130
140
150
160
170
180
190

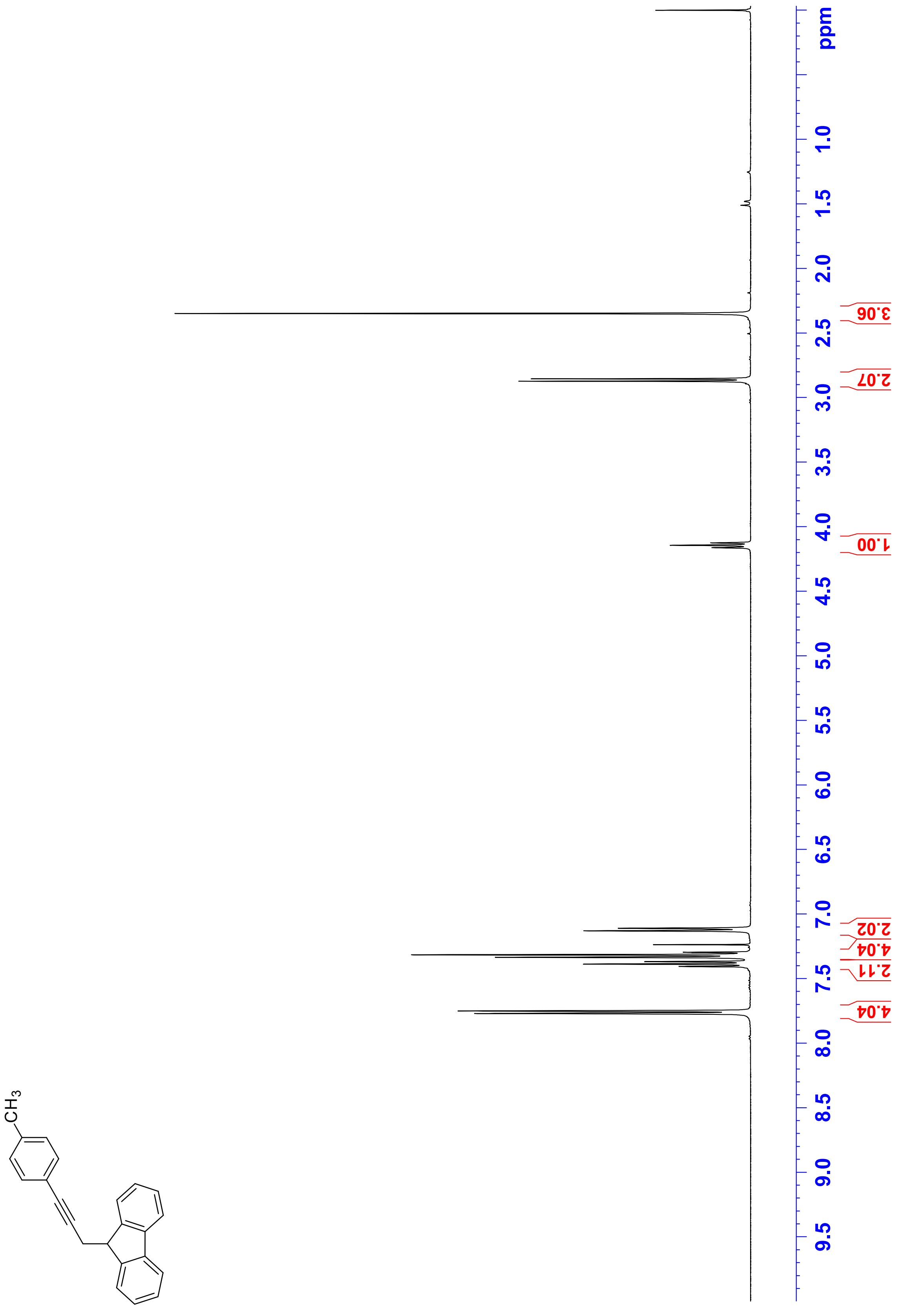
CH3
ppm
1.0
1.5
2.0
2.5
3.0
3.5
4.0
4.5
5.0
5.5
6.0
6.5
7.0
7.5
8.0
8.5
9.0
9.5
3.06
2.07
1.00
2.02
4.04
2.11
4.04

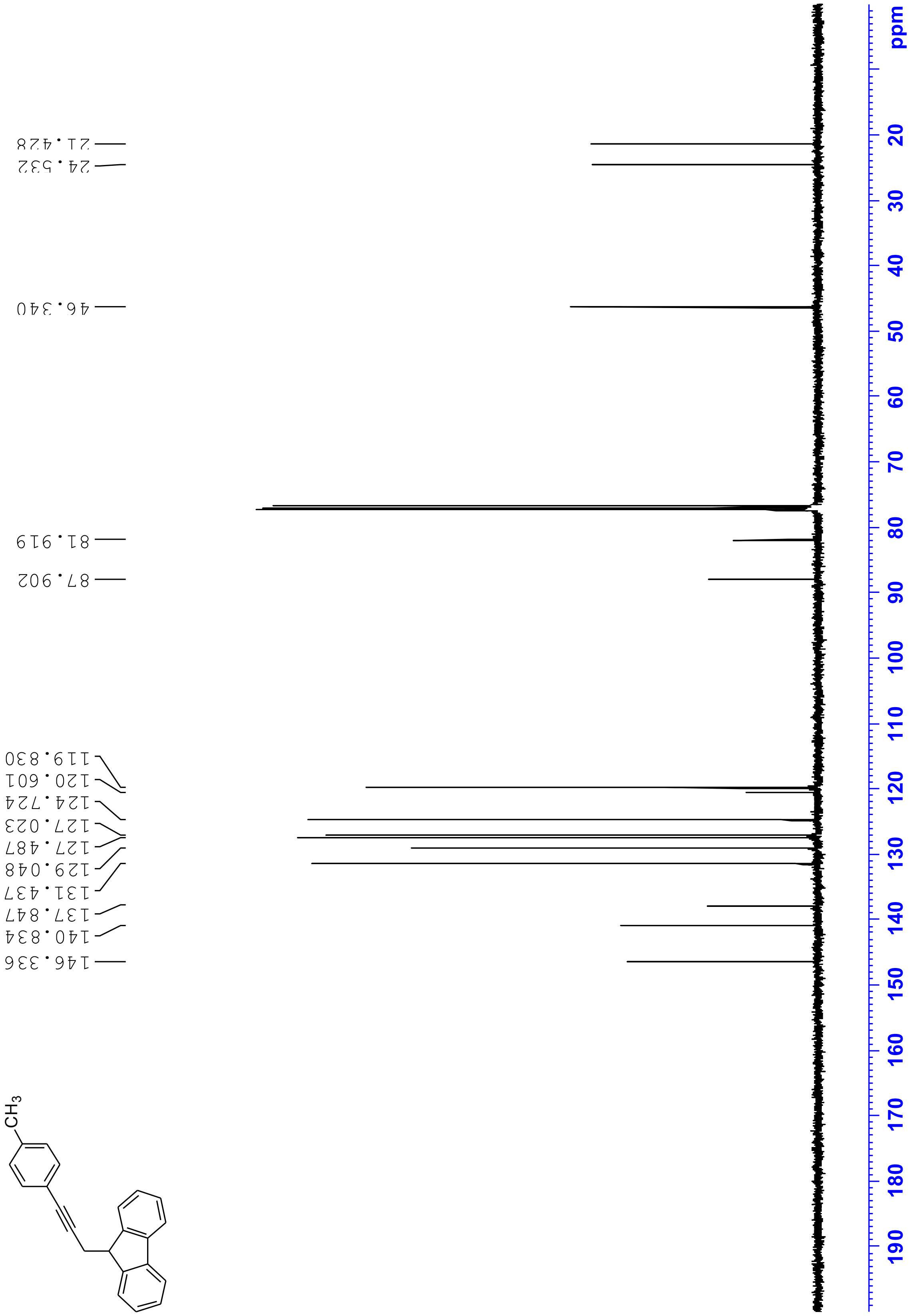
21.428
24.532
46.340
81.919
87.902
119.830
120.601
124.724
127.023
127.487
129.048
131.437
137.847
140.834
146.336
CH3
ppm
20
30
40
50
60
70
80
90
100
110
120
130
140
150
160
170
180
190

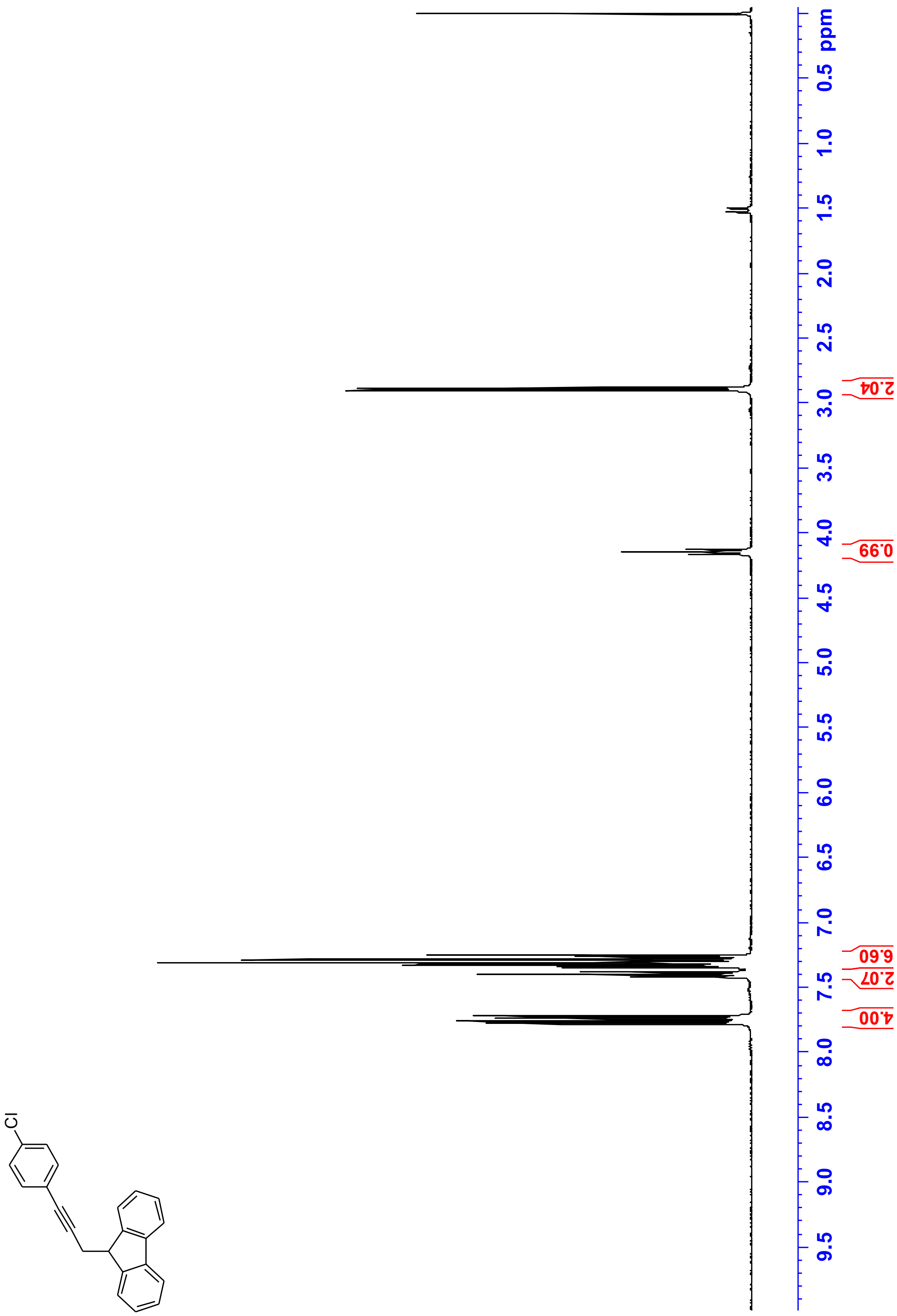

Cl
ppm
0.5
1.0
1.5
2.0
2.5
3.0
3.5
4.0
4.5
5.0
5.5
6.0
6.5
7.0
7.5
8.0
8.5
9.0
9.5
2.04
0.99
6.60
2.07
4.00

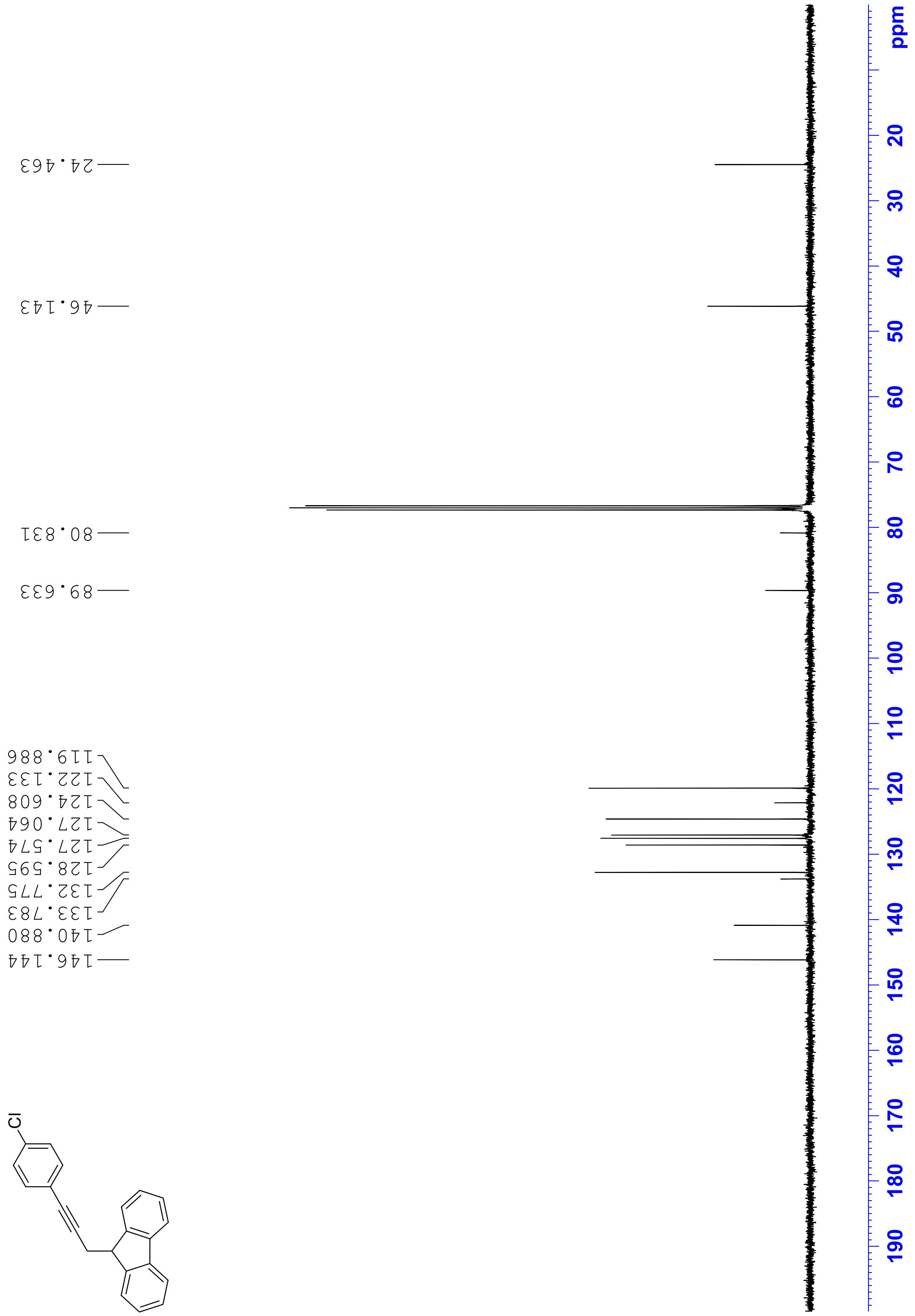

24.463
46.143
80.831
89.633
119.886
122.133
124.608
127.064
127.574
128.595
132.775
133.783
140.880
146.144
Cl
ppm
20
30
40
50
60
70
80
90
100
110
120
130
140
150
160
170
180
190

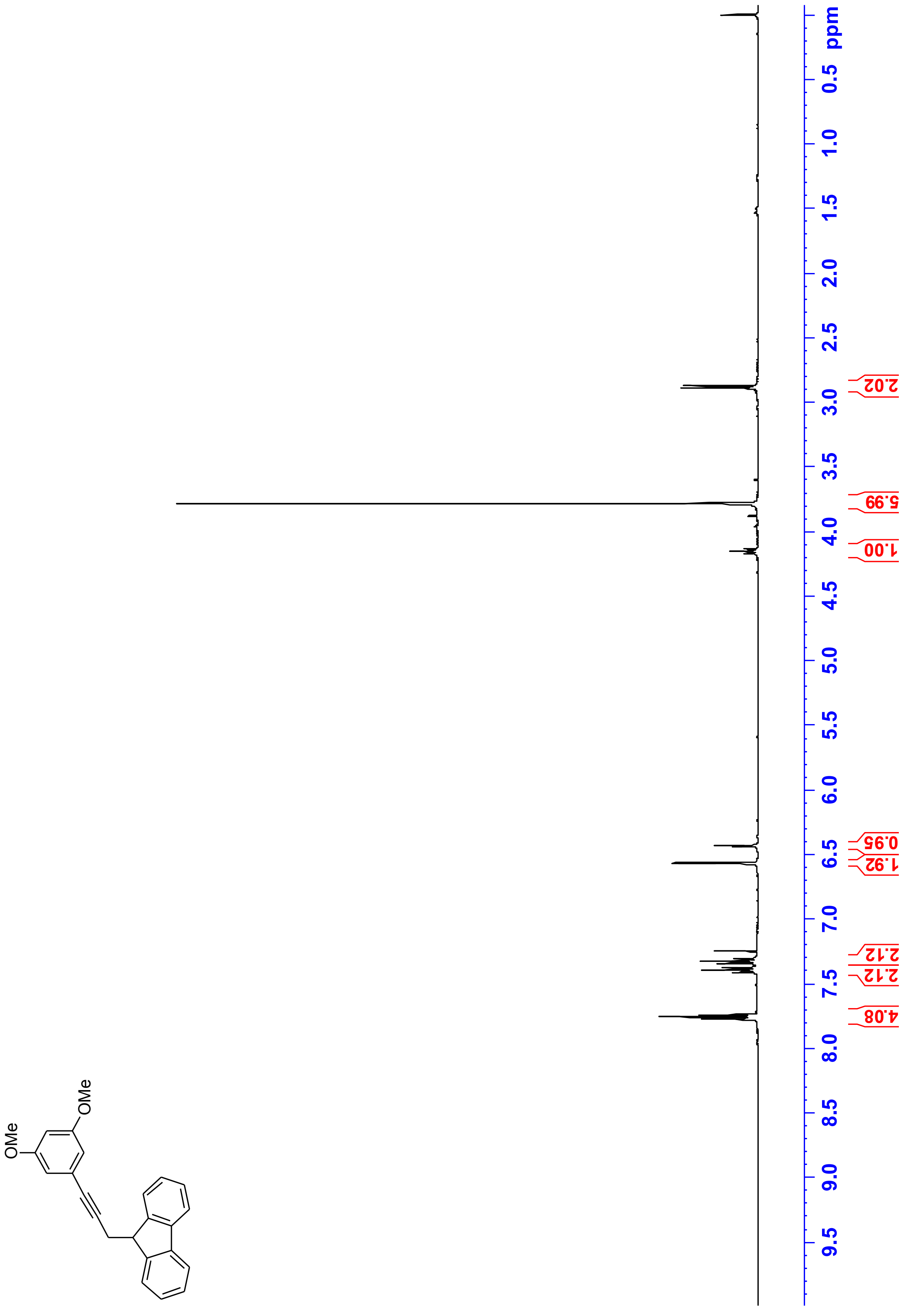
OMe
OMe
ppm
0.5
1.0
1.5
2.0
2.5
3.0
3.5
4.0
4.5
5.0
5.5
6.0
6.5
7.0
7.5
8.0
8.5
9.0
9.5
2.02
5.99
1.00
0.95
1.92
2.12
2.12
4.08

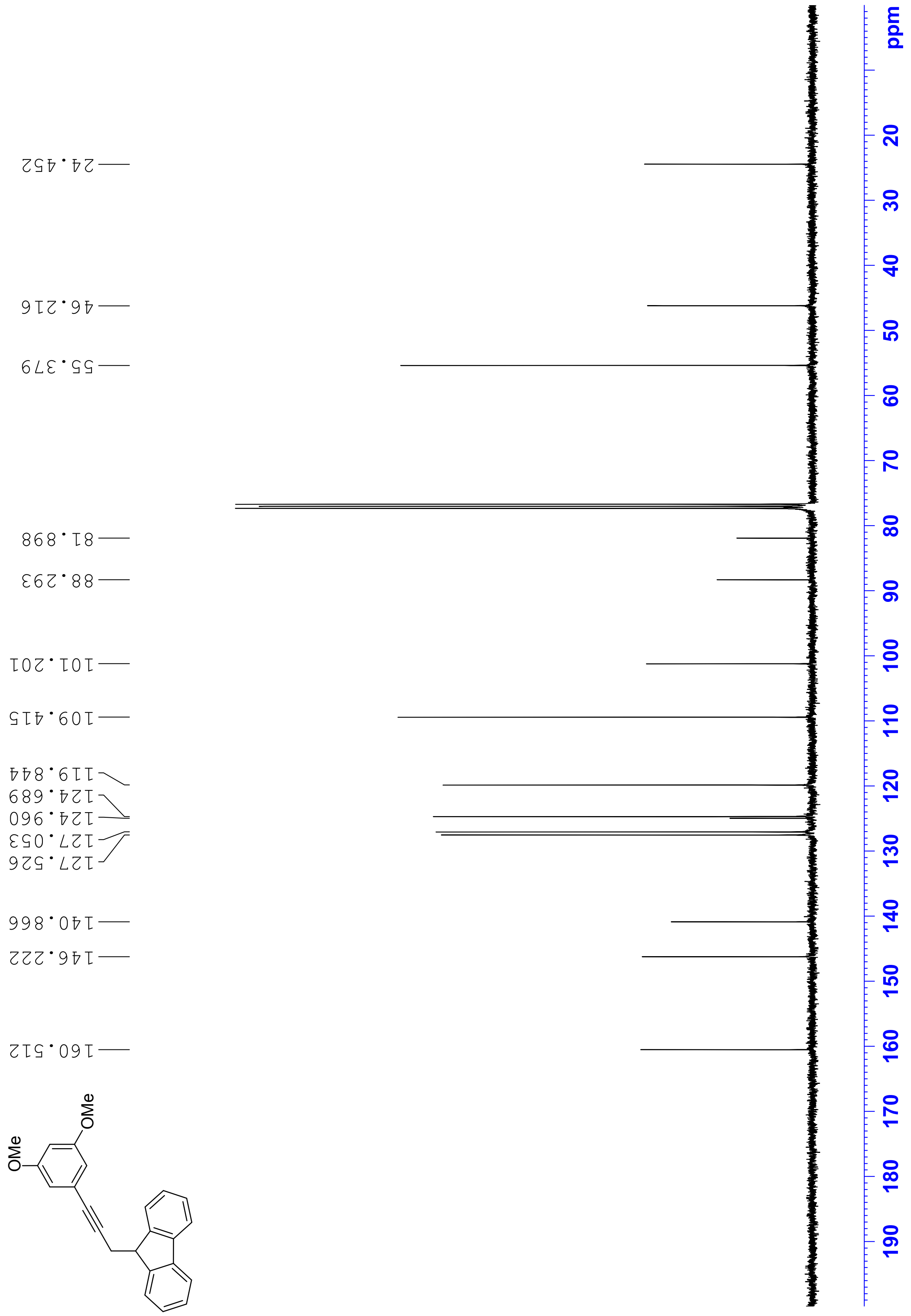
24.452
46.216
55.379
81.898
88.293
101.201
109.415
119.844
124.689
124.960
127.053
127.526
140.866
146.222
160.512
OMe
OMe
20
30
40
50
60
70
80
90
100
110
120
130
140
150
160
170
180
190
ppm

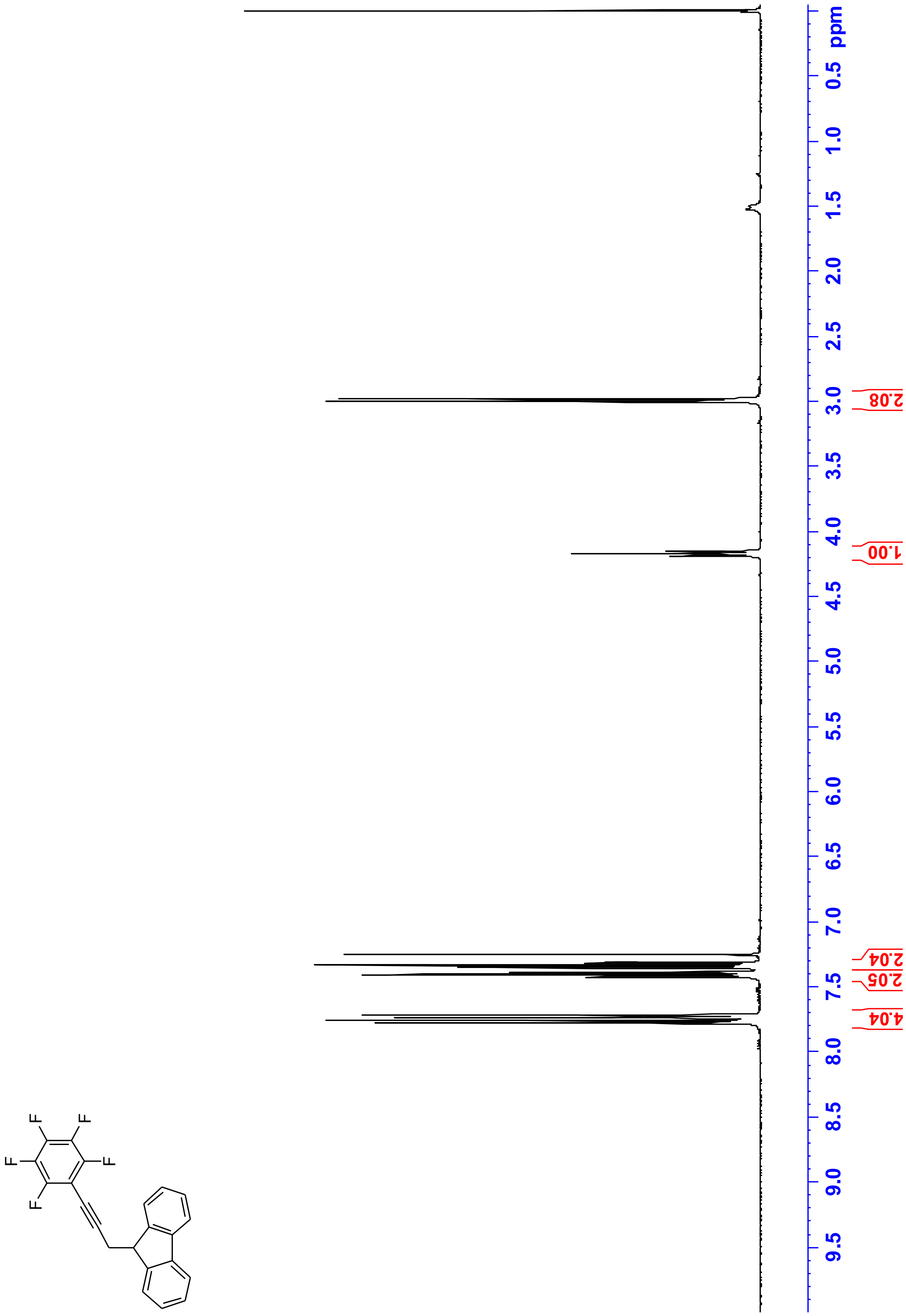
ppm
2.08
1.00
2.04
2.05
4.04

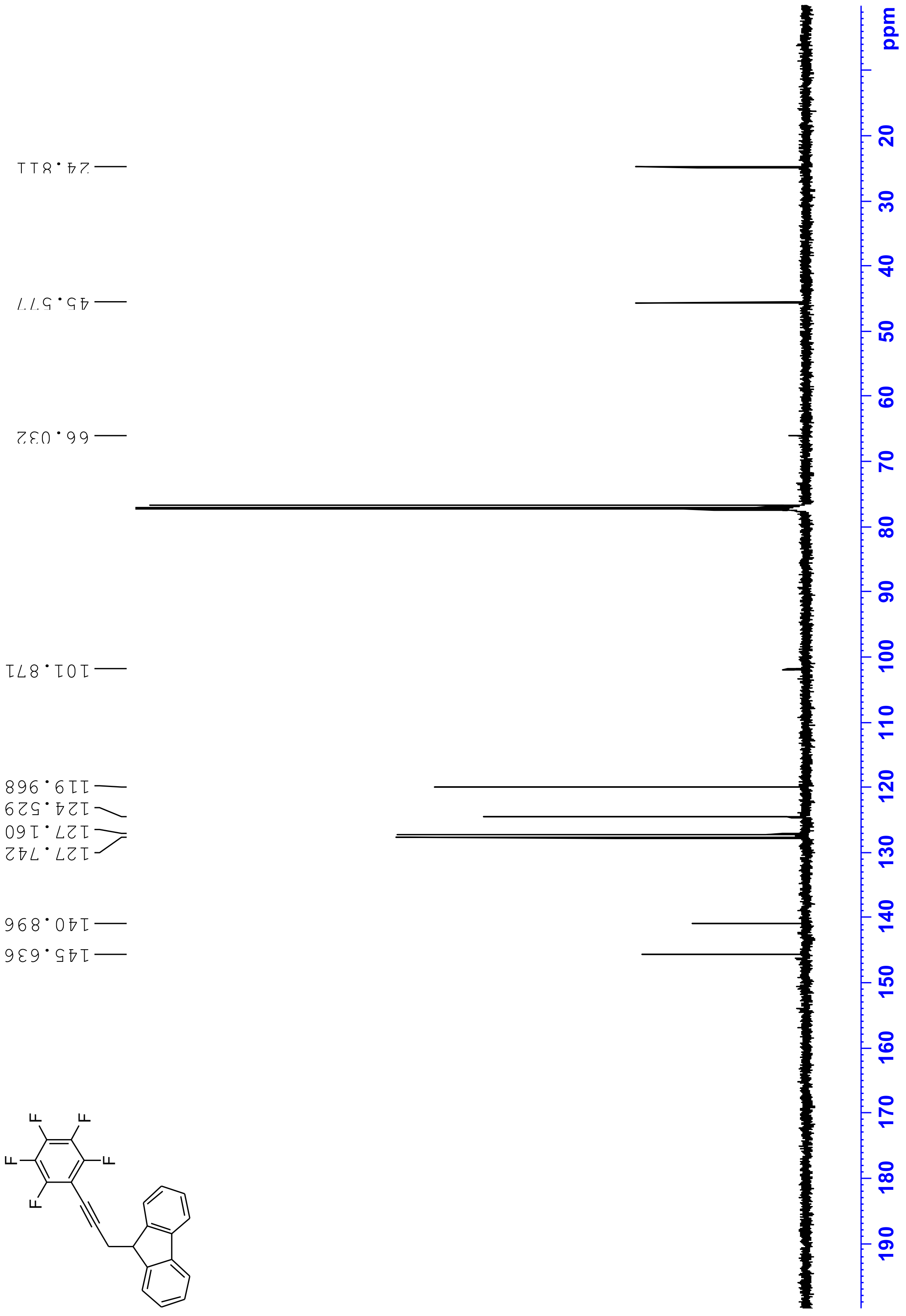

24.811
45.577
66.032
101.871
119.968
124.529
127.160
127.742
140.896
145.636
F
F
F
F
F
ppm
20
30
40
50
60
70
80
90
100
110
120
130
140
150
160
170
180
190

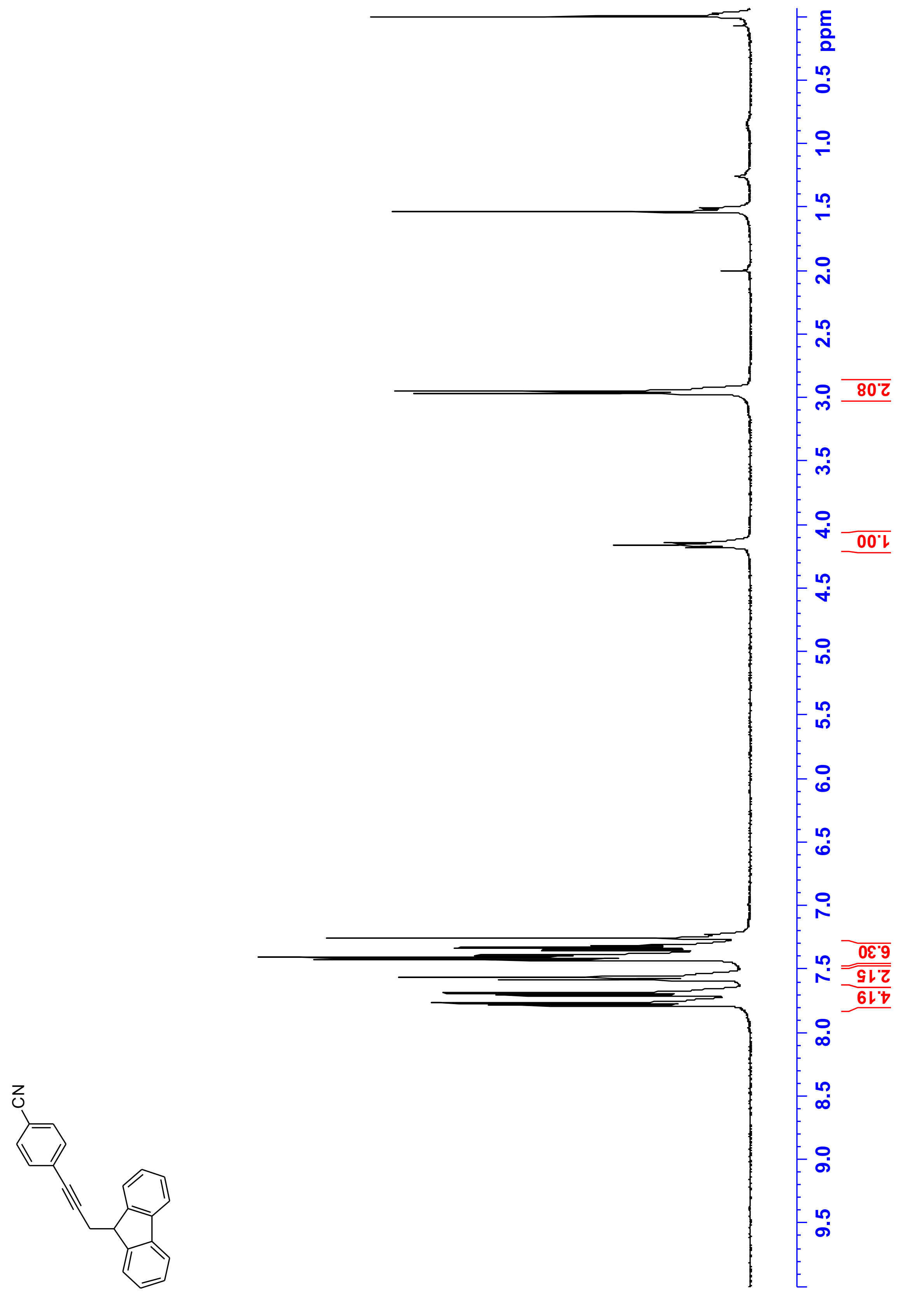
CN
ppm
0.5
1.0
1.5
2.0
2.5
3.0
3.5
4.0
4.5
5.0
5.5
6.0
6.5
7.0
7.5
8.0
8.5
9.0
9.5
2.08
1.00
6.30
2.15
4.19

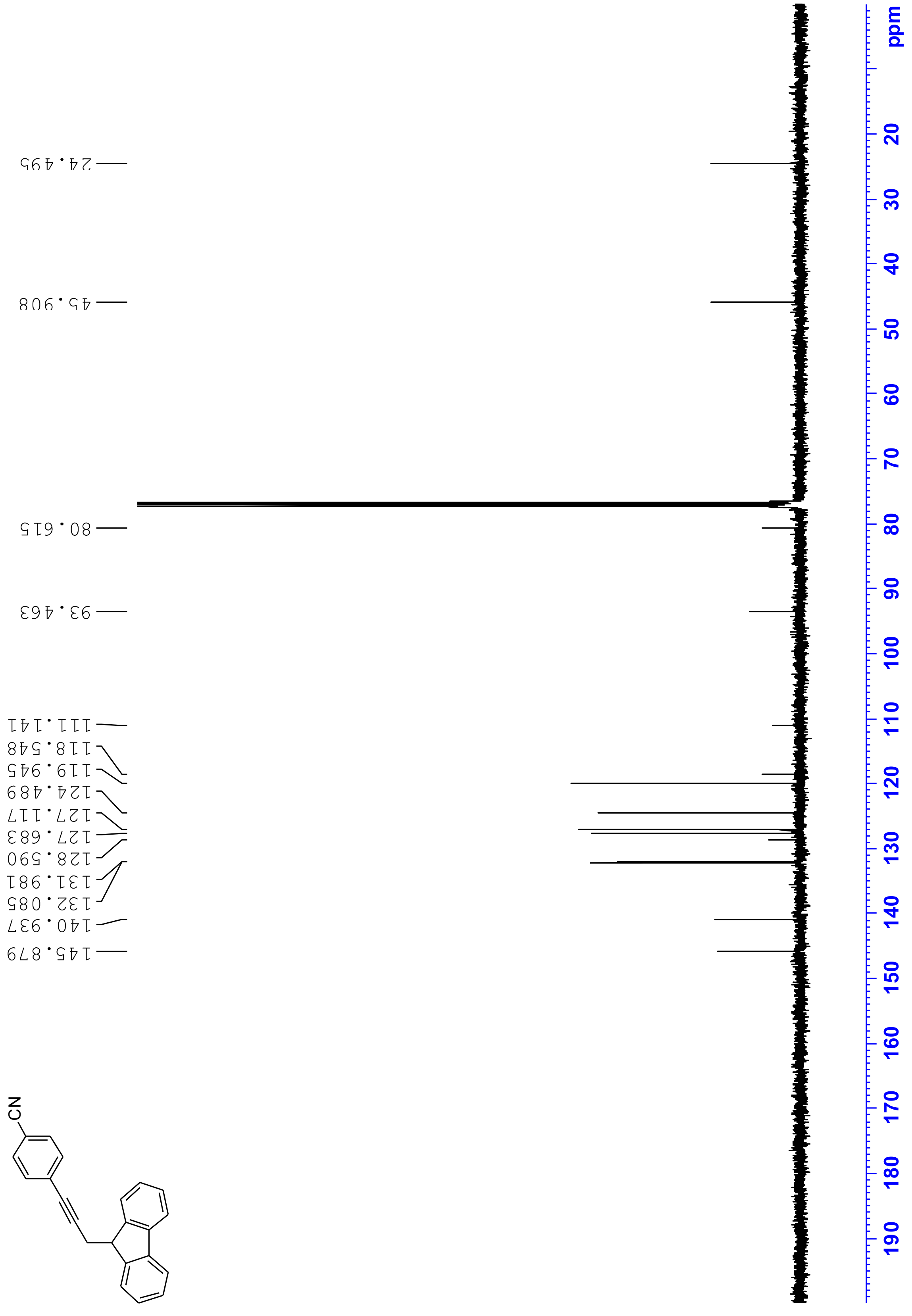
24.495
45.908
80.615
93.463
111.141
118.548
119.945
124.489
127.117
127.683
128.590
131.981
132.085
140.937
145.879
CN
ppm
20
30
40
50
60
70
80
90
100
110
120
130
140
150
160
170
180
190

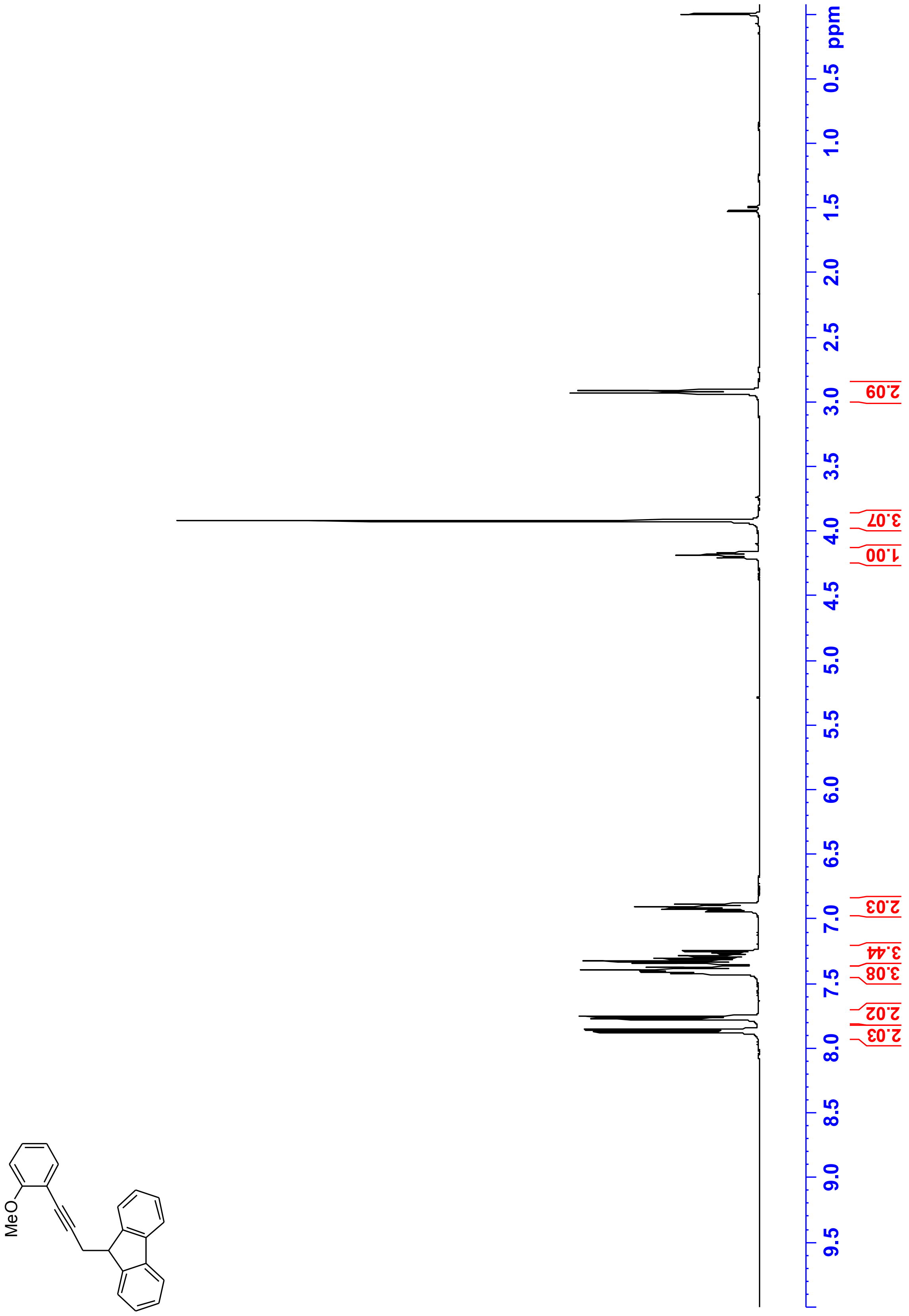
MeO
ppm
0.5
1.0
1.5
2.0
2.5
3.0
3.5
4.0
4.5
5.0
5.5
6.0
6.5
7.0
7.5
8.0
8.5
9.0
9.5
2.09
3.07
1.00
2.03
3.44
3.08
2.02
2.03

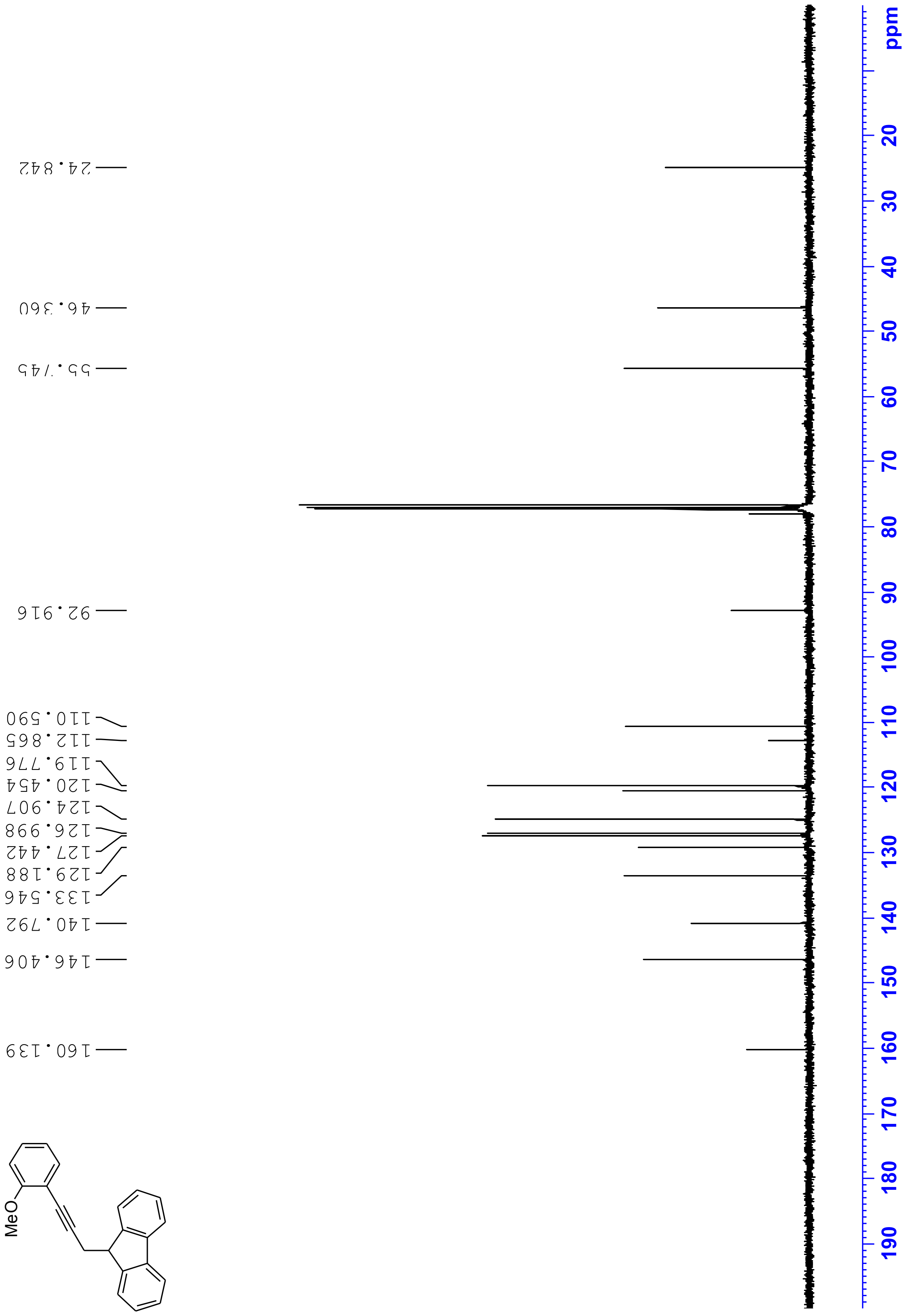

24.842
46.360
55.745
92.916
110.590
112.865
119.776
120.454
124.907
126.998
127.442
129.188
133.546
140.792
146.406
160.139
MeO
ppm
20
30
40
50
60
70
80
90
100
110
120
130
140
150
160
170
180
190

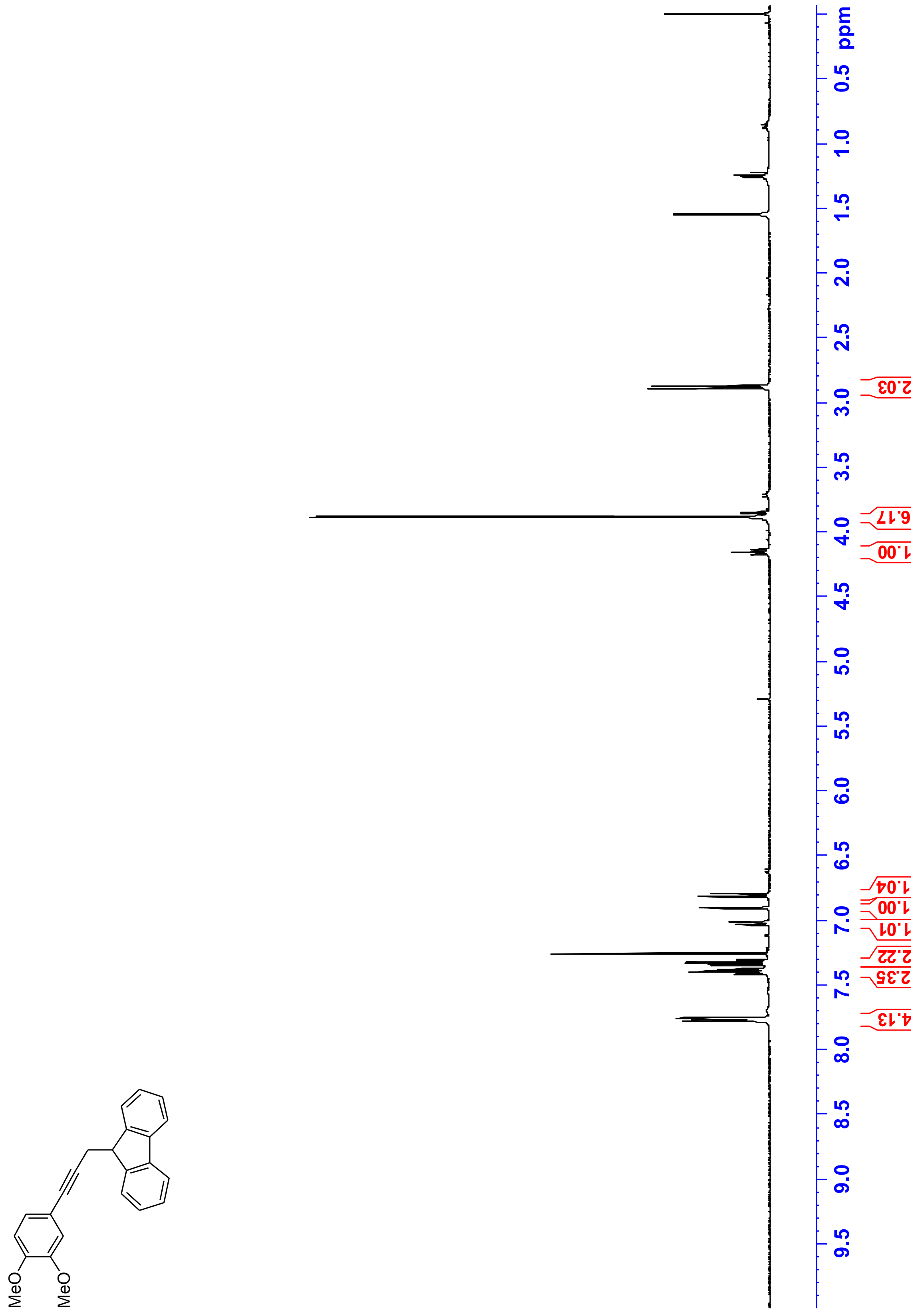
ppm
0.5
1.0
1.5
2.0
2.5
3.0
3.5
4.0
4.5
5.0
5.5
6.0
6.5
7.0
7.5
8.0
8.5
9.0
9.5
2.03
6.17
1.00
1.04
1.00
1.01
2.22
2.35
4.13
MeO
MeO

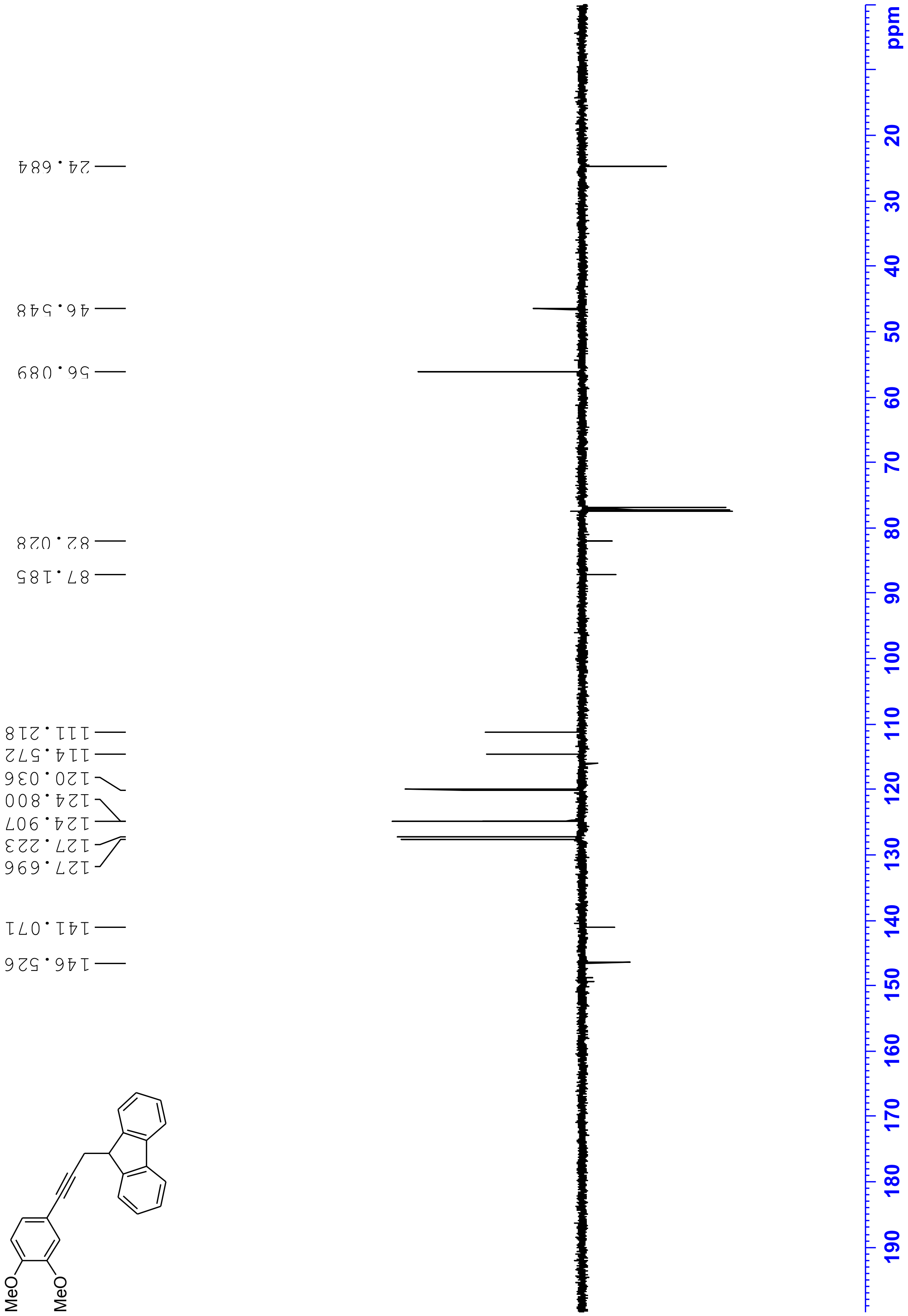
24.684
46.548
56.089
82.028
87.185
111.218
114.572
120.036
124.800
124.907
127.223
127.696
141.071
146.526
MeO
MeO
ppm
20
30
40
50
60
70
80
90
100
110
120
130
140
150
160
170
180
190

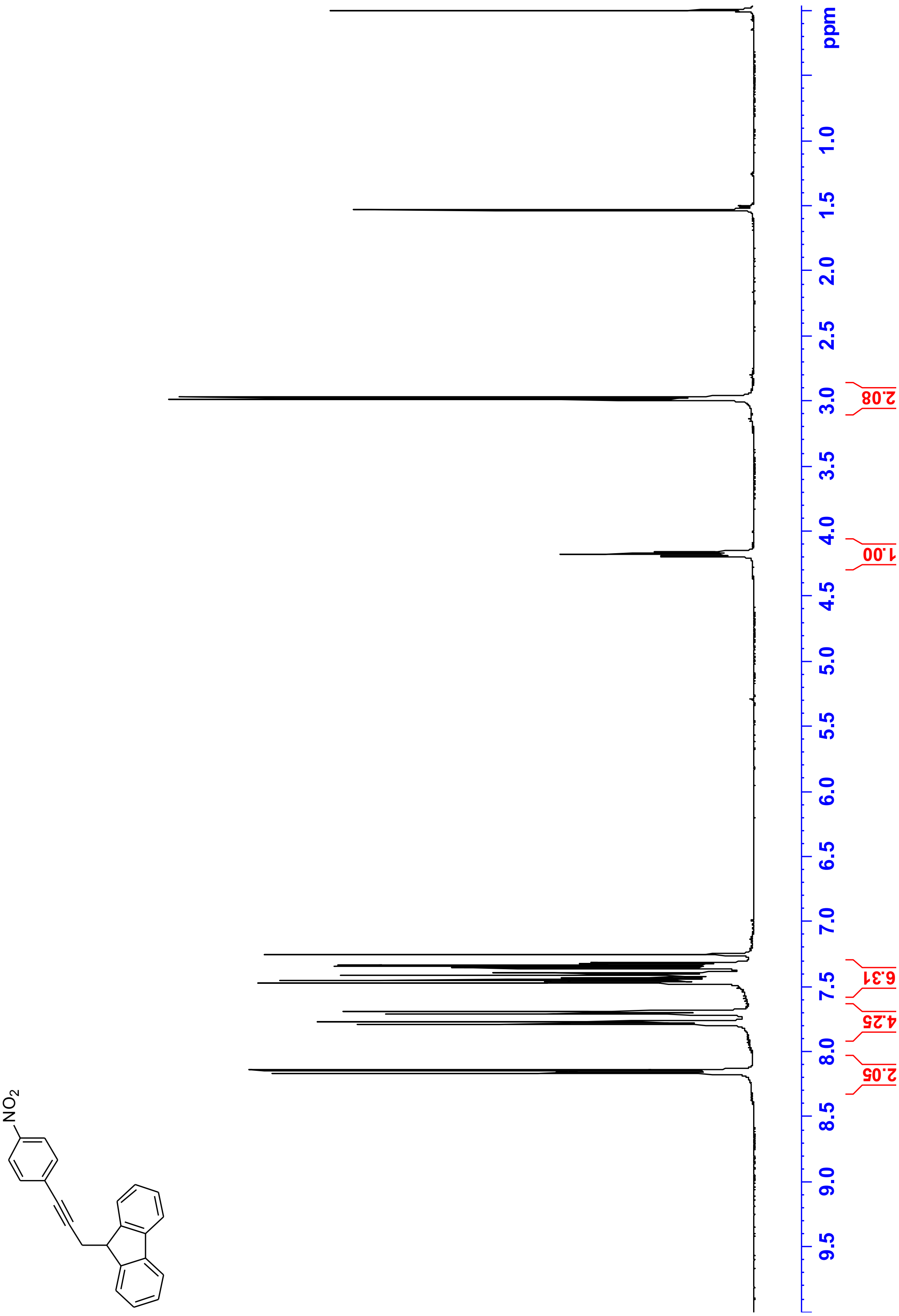

ppm
1.0
1.5
2.0
2.5
3.0
3.5
4.0
4.5
5.0
5.5
6.0
6.5
7.0
7.5
8.0
8.5
9.0
9.5
2.08
1.00
6.31
4.25
2.05
NO_2

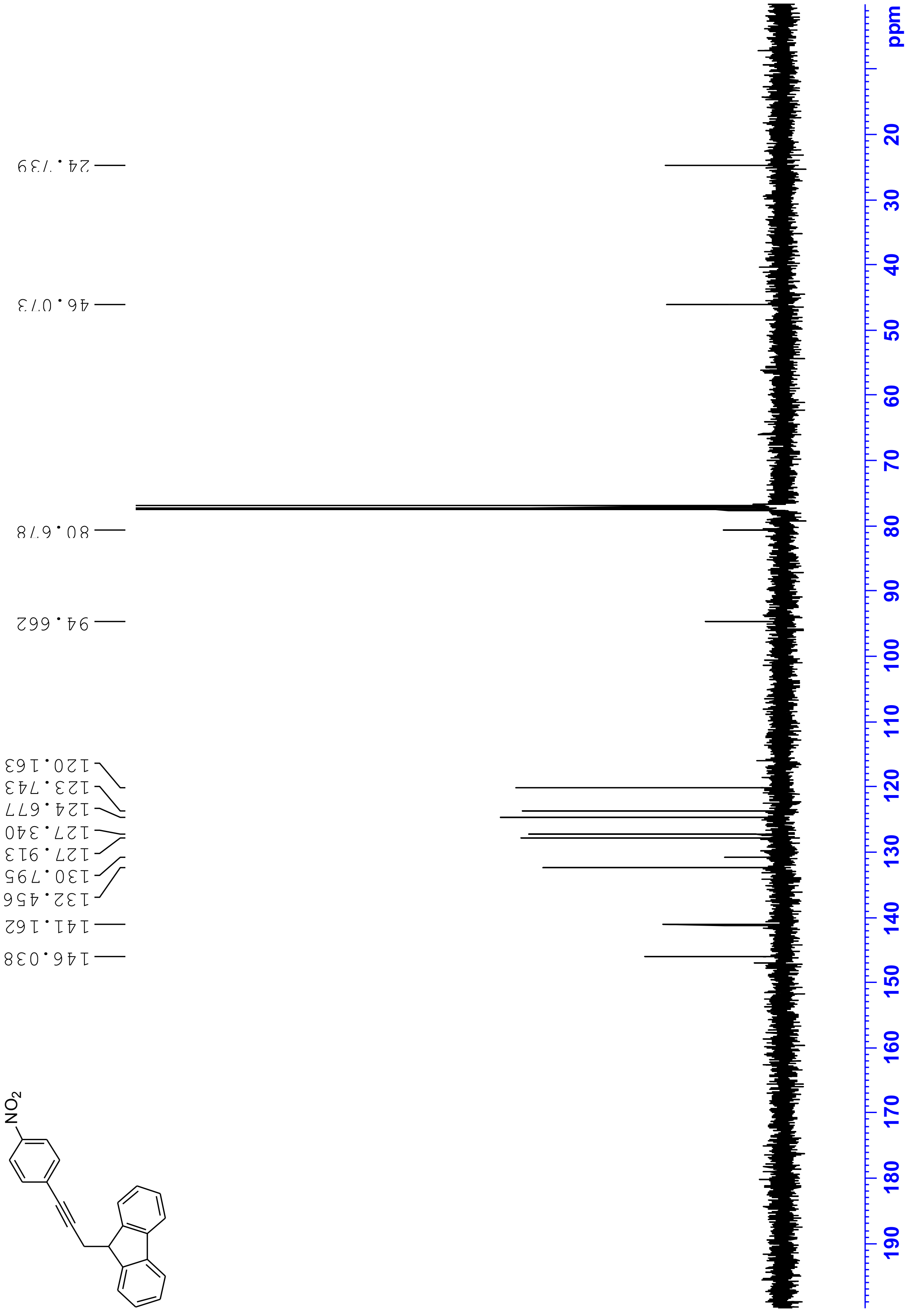

24.739
46.073
80.678
94.662
120.163
123.743
124.677
127.340
127.913
130.795
132.456
141.162
146.038
NO_2
ppm
20
30
40
50
60
70
80
90
100
110
120
130
140
150
160
170
180
190

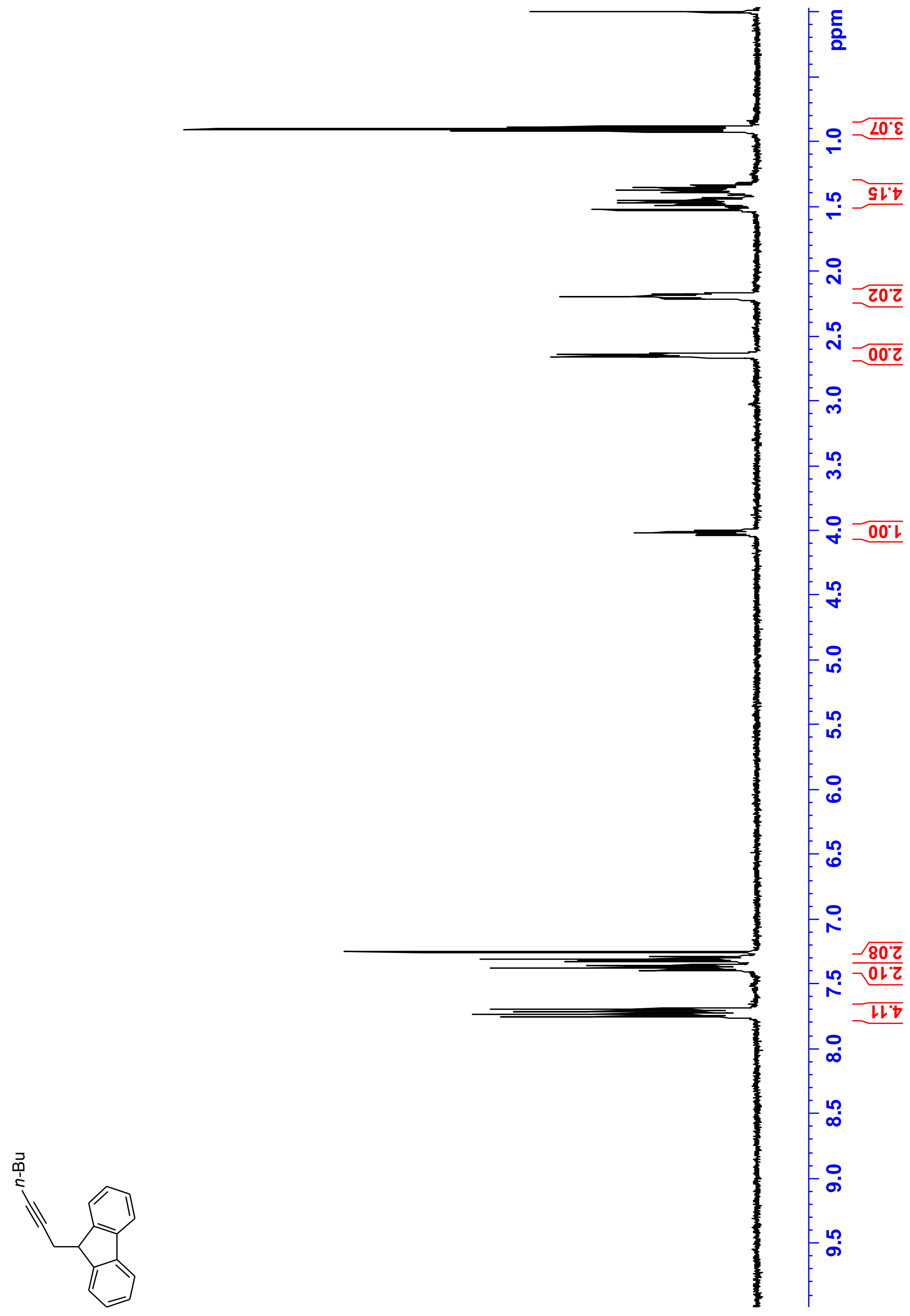

n-Bu
ppm
1.0
1.5
2.0
2.5
3.0
3.5
4.0
4.5
5.0
5.5
6.0
6.5
7.0
7.5
8.0
8.5
9.0
9.5
3.07
4.15
2.02
2.00
1.00
2.08
2.10
4.11

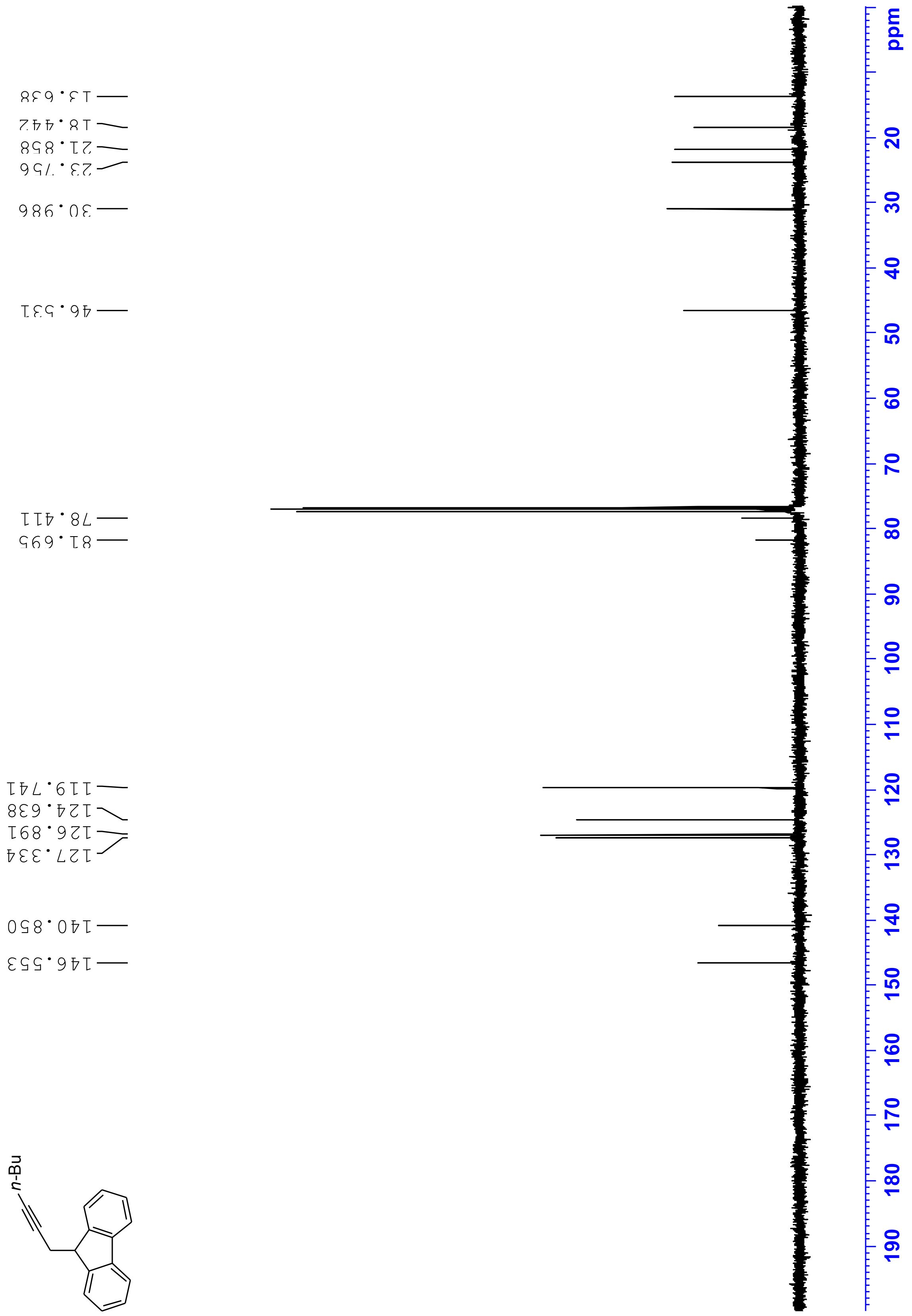

13.638
18.442
21.858
23.756
30.986
46.531
78.411
81.695
119.741
124.638
126.891
127.334
140.850
146.553
n-Bu
ppm
20
30
40
50
60
70
80
90
100
110
120
130
140
150
160
170
180
190

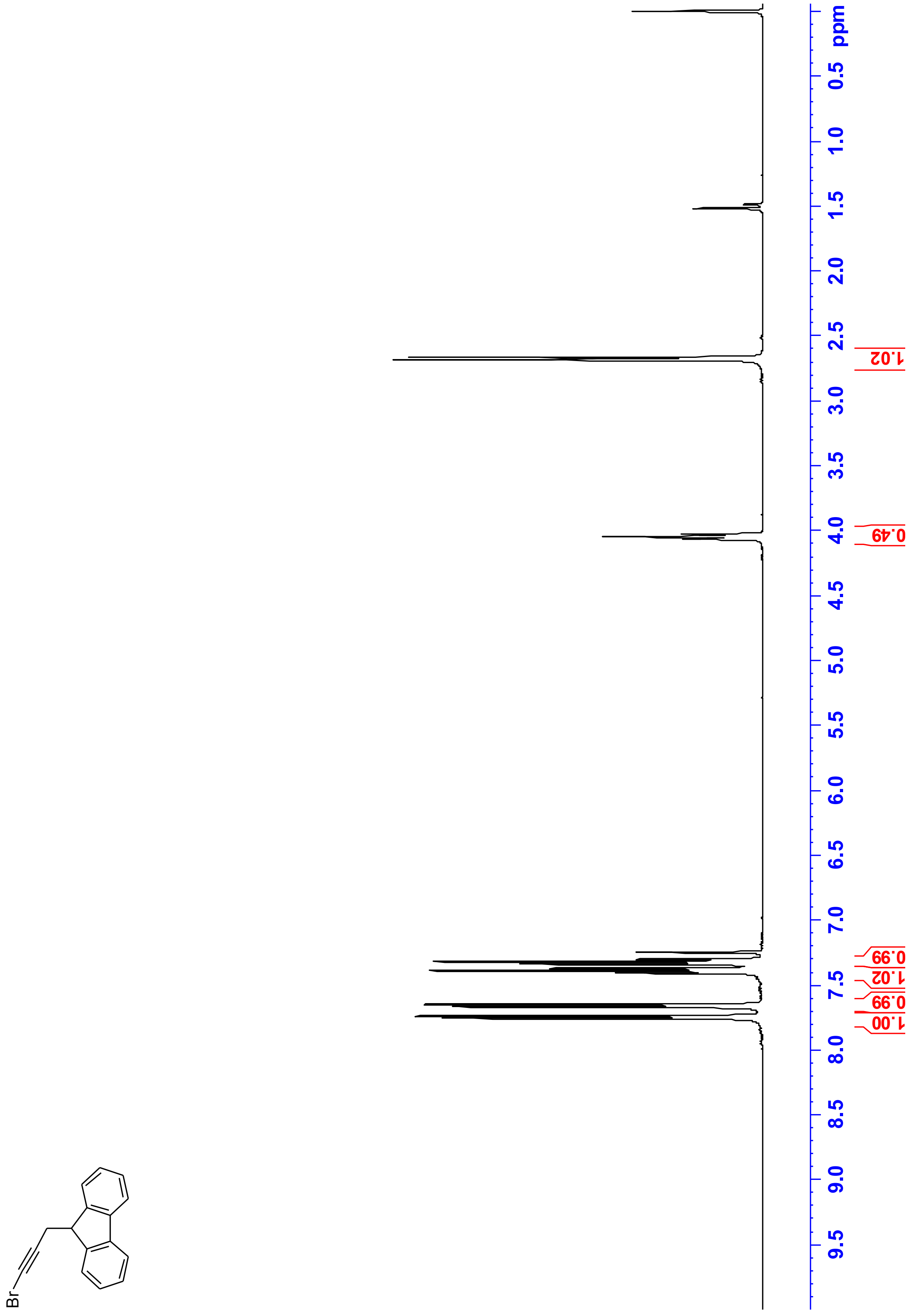
Br
ppm
0.5
1.0
1.5
2.0
2.5
3.0
3.5
4.0
4.5
5.0
5.5
6.0
6.5
7.0
7.5
8.0
8.5
9.0
9.5
1.02
0.49
0.99
1.02
0.99
1.00

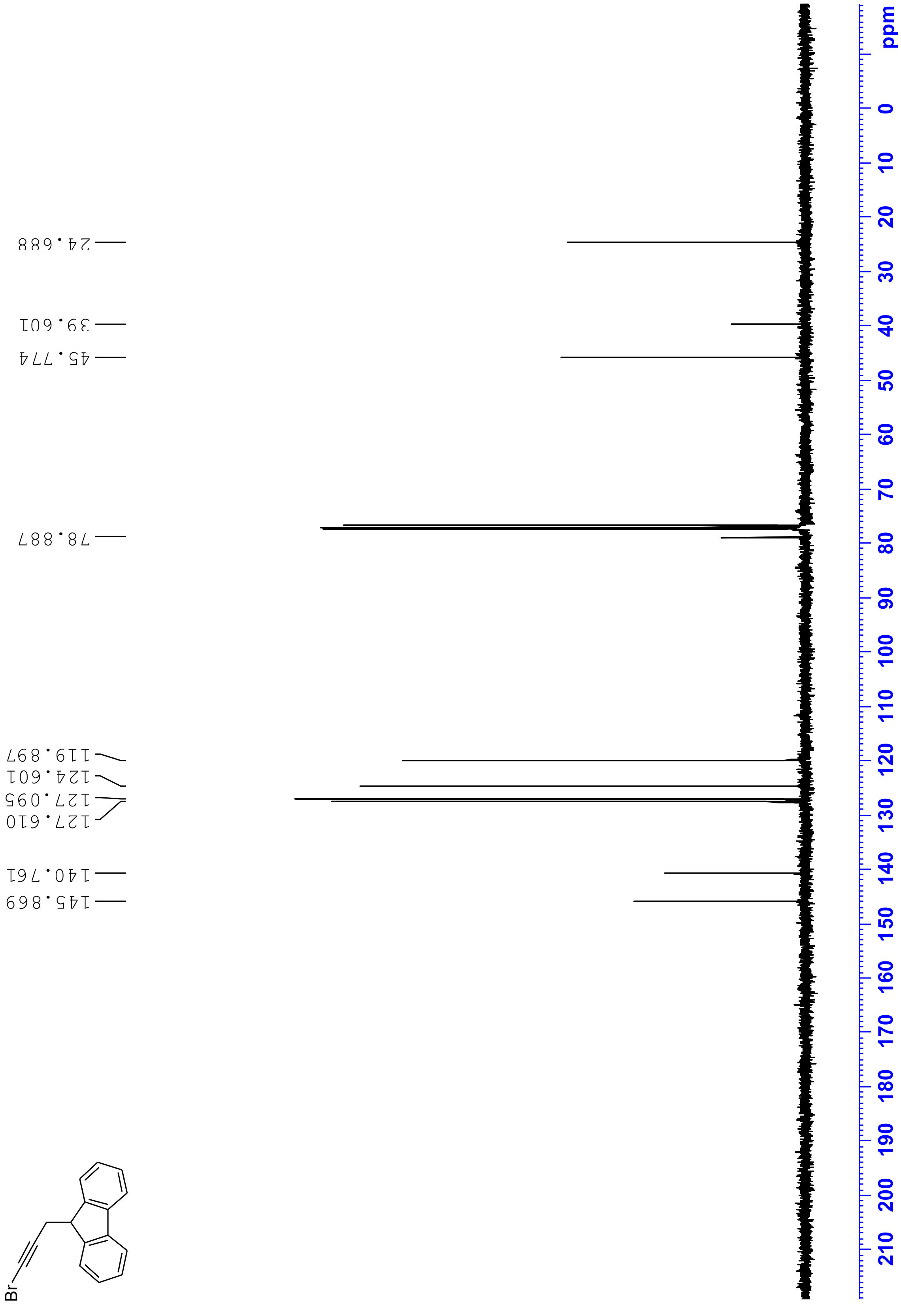
24.688
39.601
45.774
78.887
119.897
124.601
127.095
127.610
140.761
145.869
Br
ppm
0
10
20
30
40
50
60
70
80
90
100
110
120
130
140
150
160
170
180
190
200
210

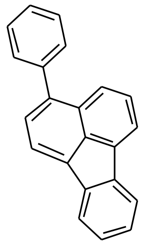

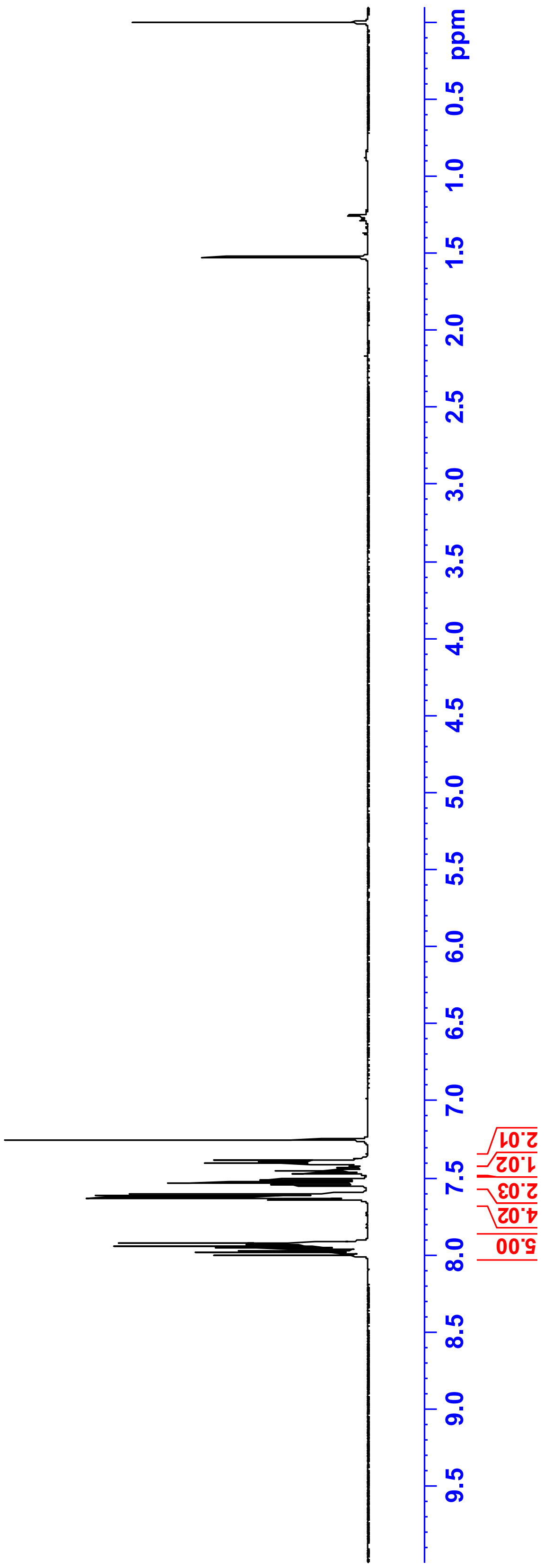
ppm
0.5
1.0
1.5
2.0
2.5
3.0
3.5
4.0
4.5
5.0
5.5
6.0
6.5
7.0
7.5
8.0
8.5
9.0
9.5
2.01
1.02
2.03
4.02
5.00

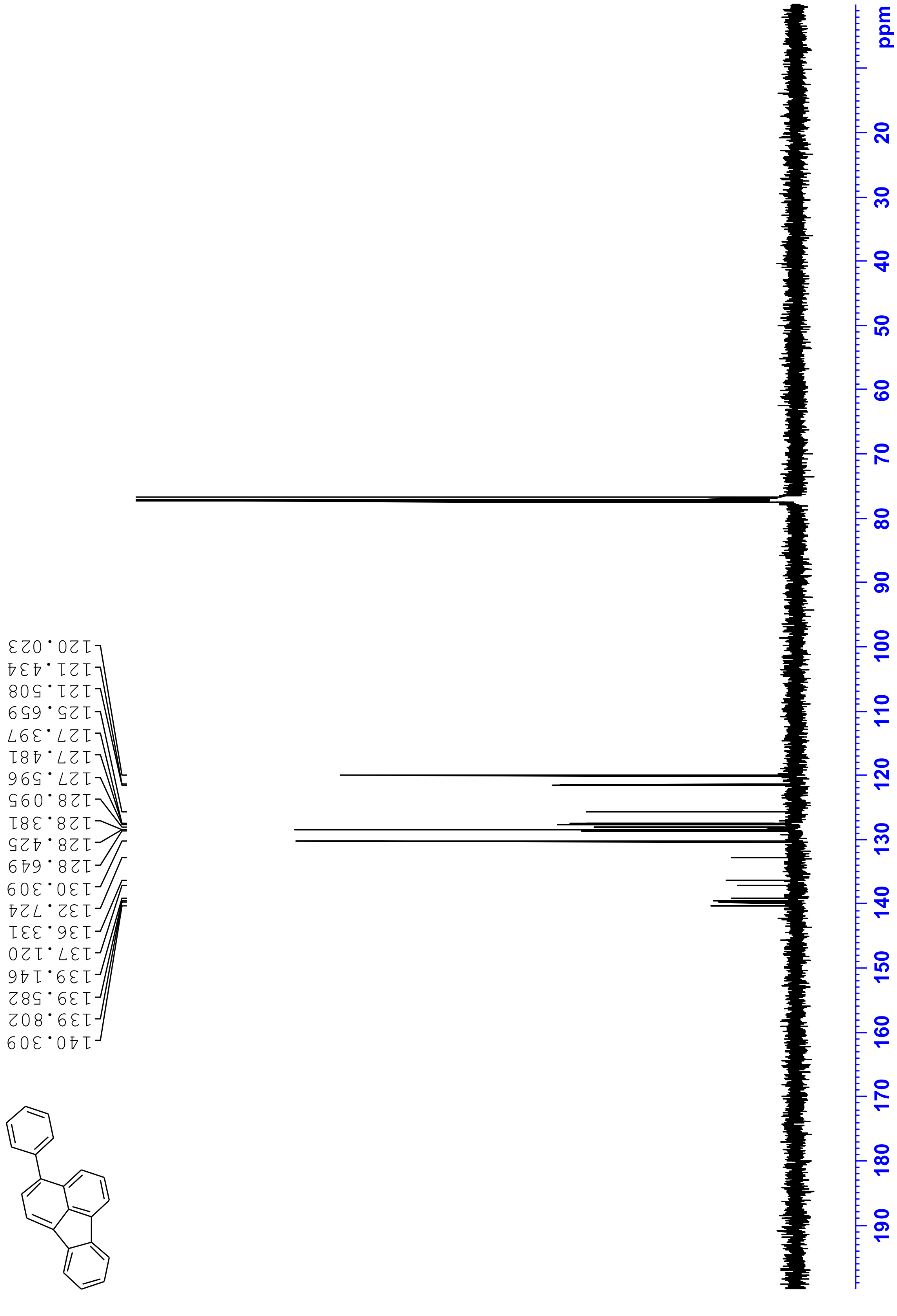
140.309
139.802
139.582
139.146
137.120
136.331
132.724
130.309
128.649
128.425
128.381
128.095
127.596
127.481
127.397
125.659
121.508
121.434
120.023
190
180
170
160
150
140
130
120
110
100
90
80
70
60
50
40
30
20
ppm

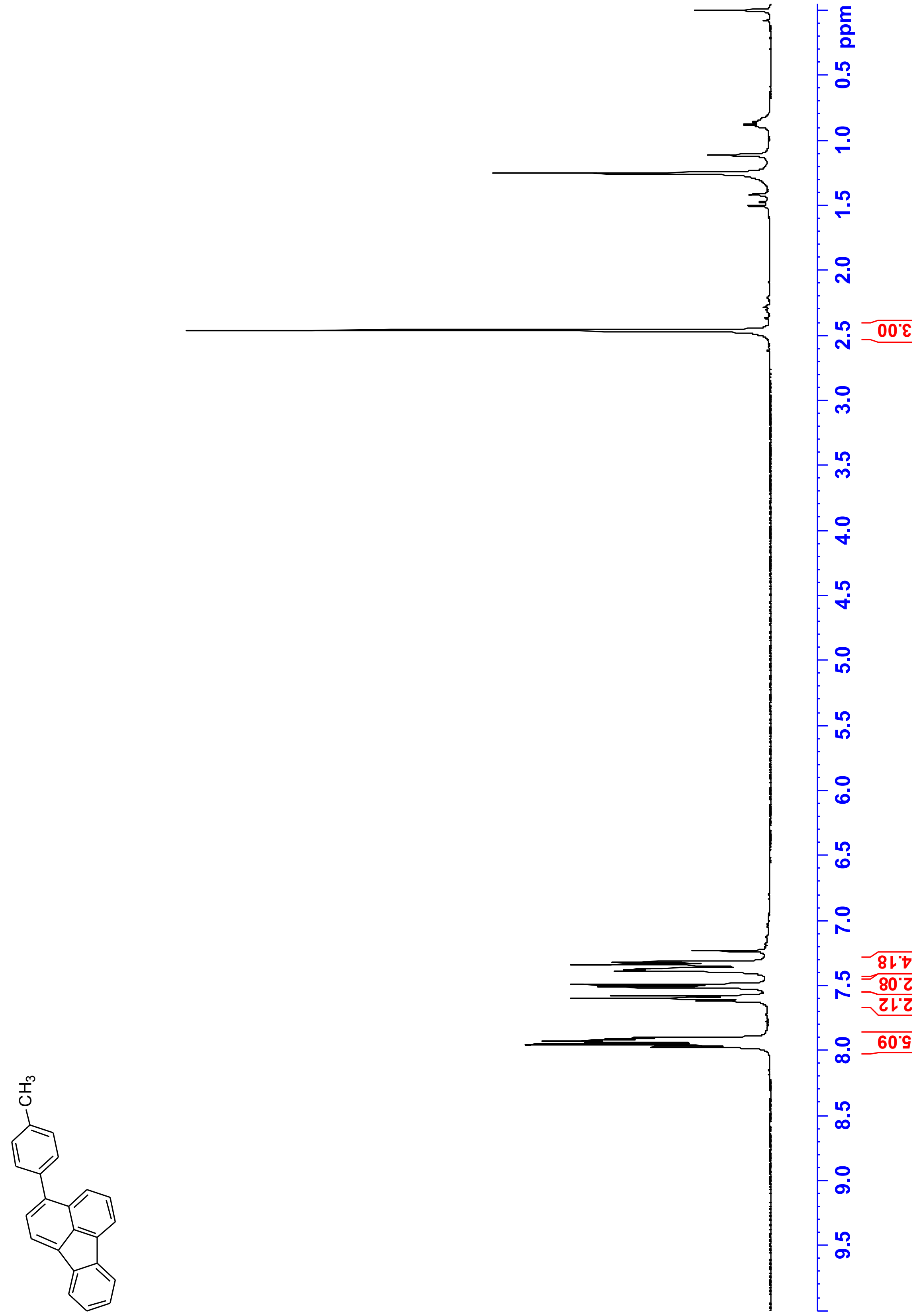

CH3
ppm
3.00
4.18
2.08
2.12
5.09

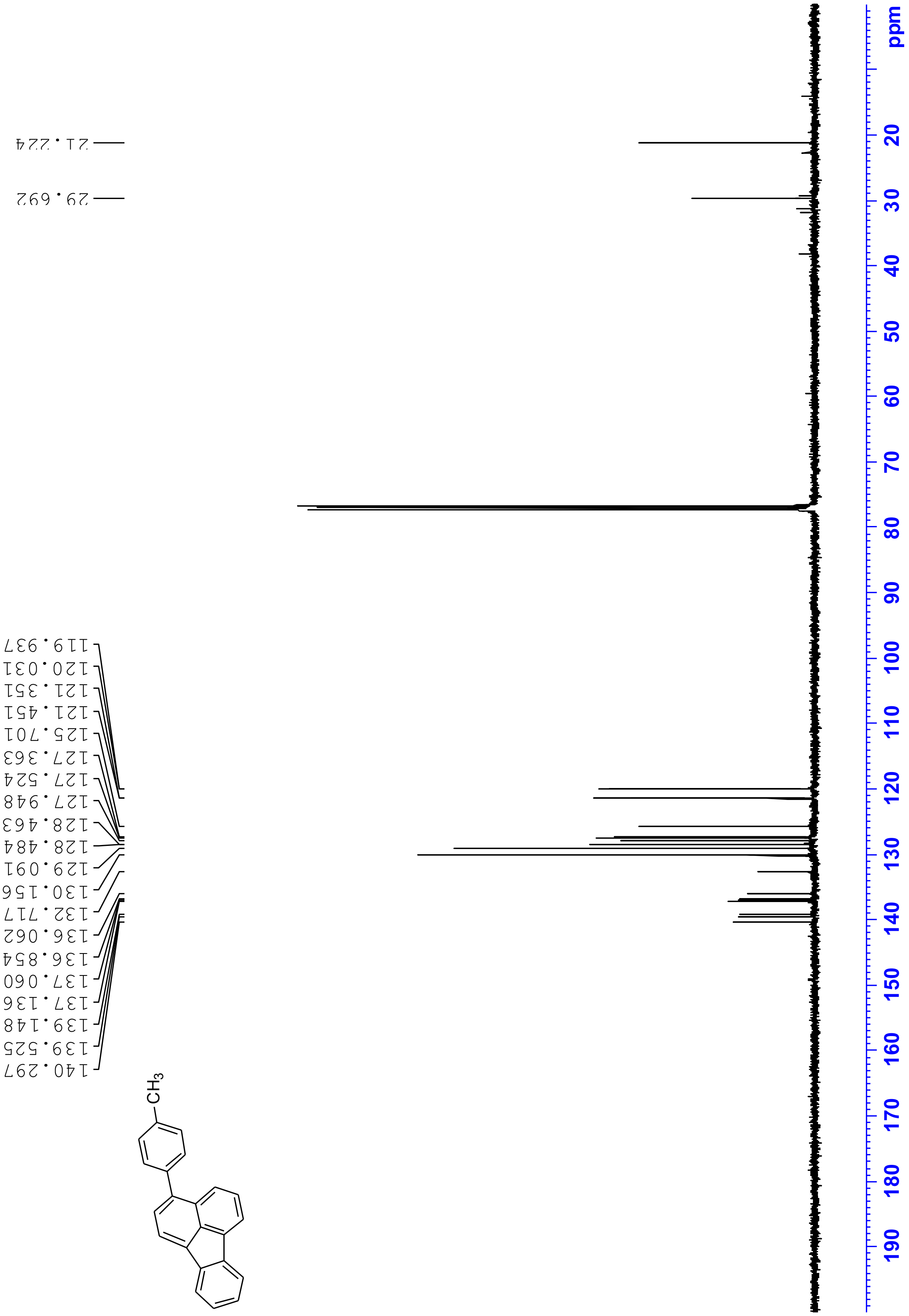
21.224
29.692
119.937
120.031
121.351
121.451
125.701
127.363
127.524
127.948
128.463
128.484
129.091
130.156
132.717
136.062
136.854
137.060
137.136
139.148
139.525
140.297
CH_3
ppm

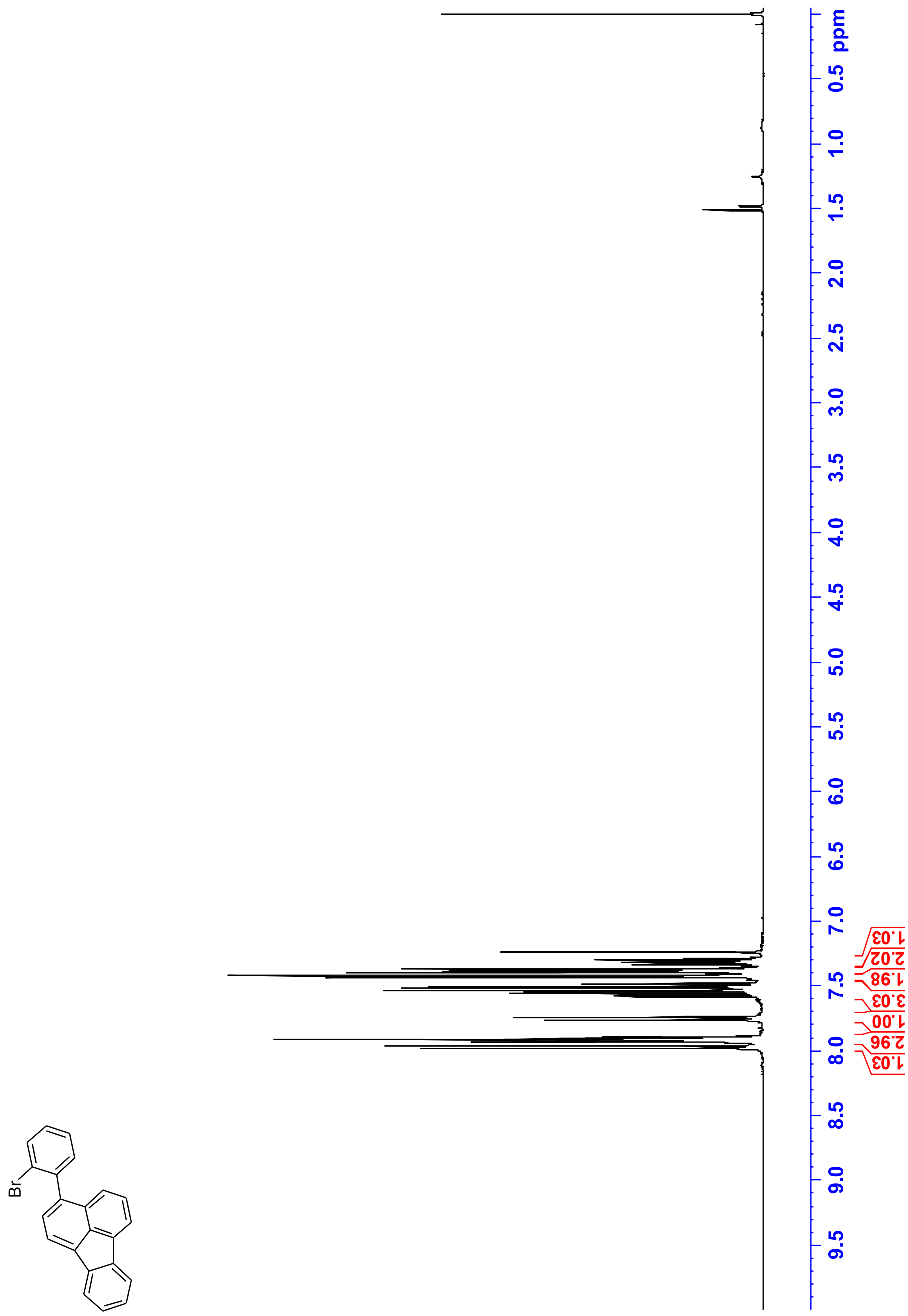
Br
ppm
0.5
1.0
1.5
2.0
2.5
3.0
3.5
4.0
4.5
5.0
5.5
6.0
6.5
7.0
7.5
8.0
8.5
9.0
9.5
1.03
2.02
1.98
3.03
1.00
2.96
1.03

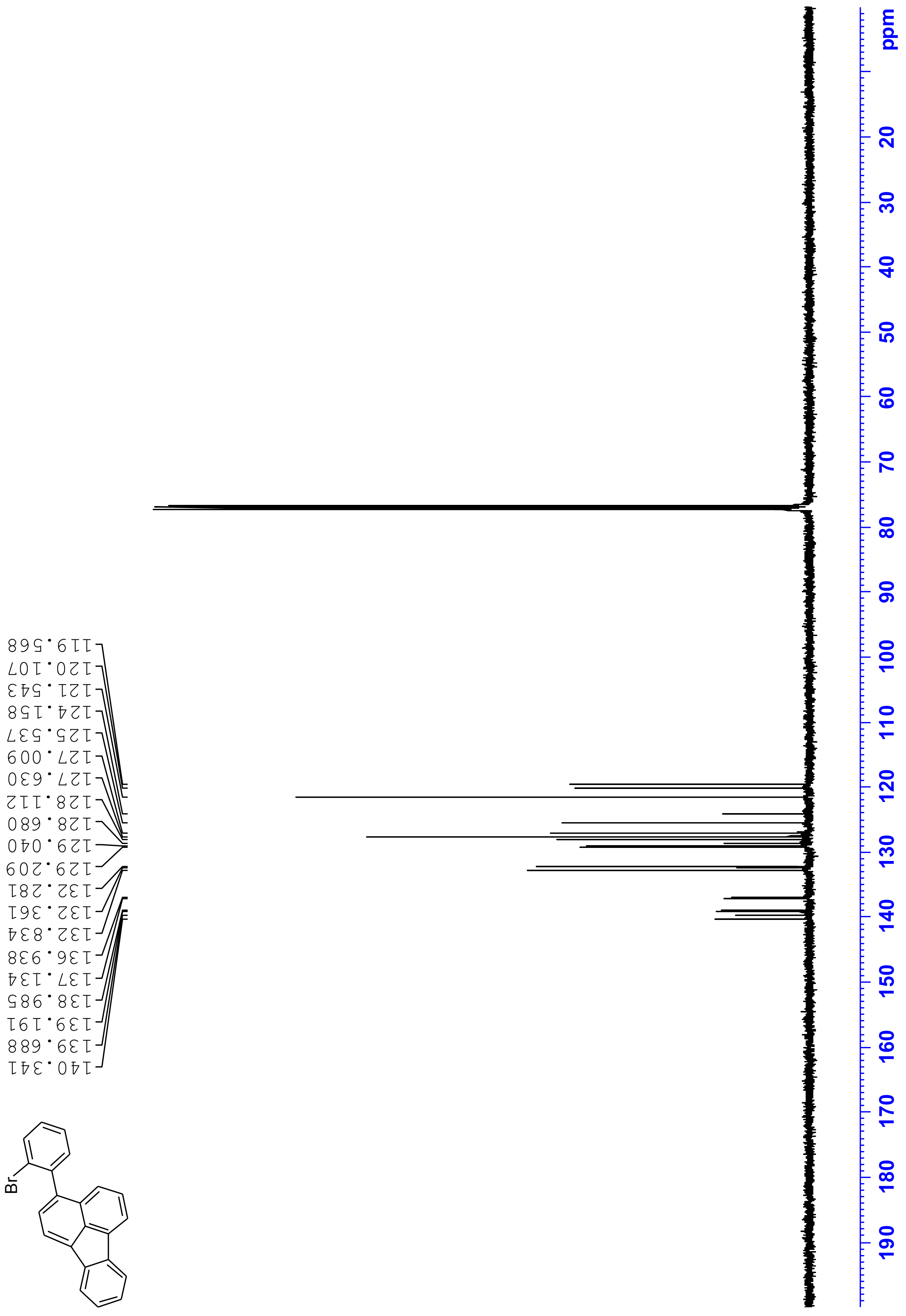
Br
140.341
139.688
139.191
138.985
137.134
136.938
132.834
132.361
132.281
129.209
129.040
128.680
128.112
127.630
127.009
125.537
124.158
121.543
120.107
119.568
190
180
170
160
150
140
130
120
110
100
90
80
70
60
50
40
30
20
ppm

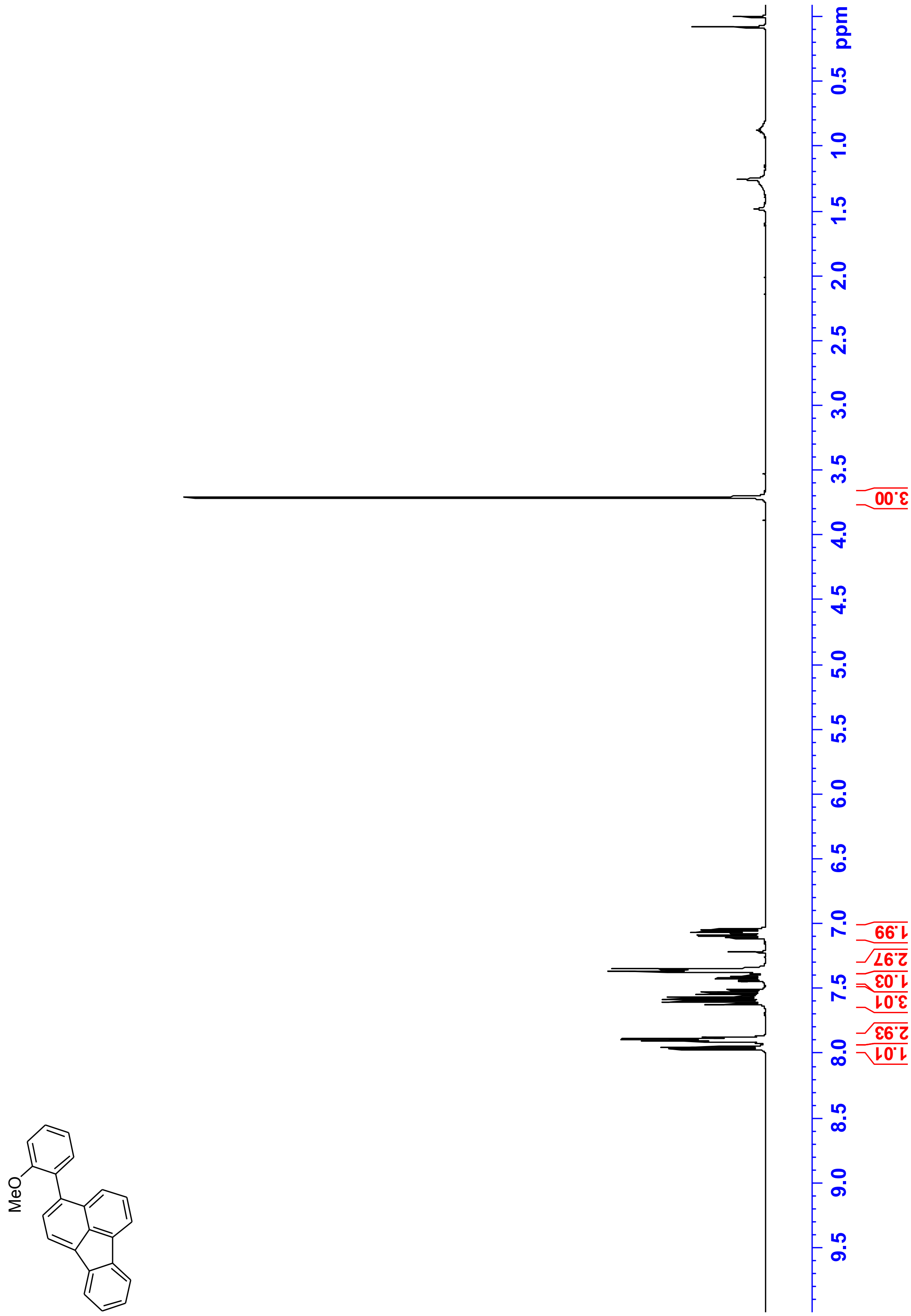
MeO
ppm
3.00
1.99
2.97
1.03
3.01
2.93
1.01

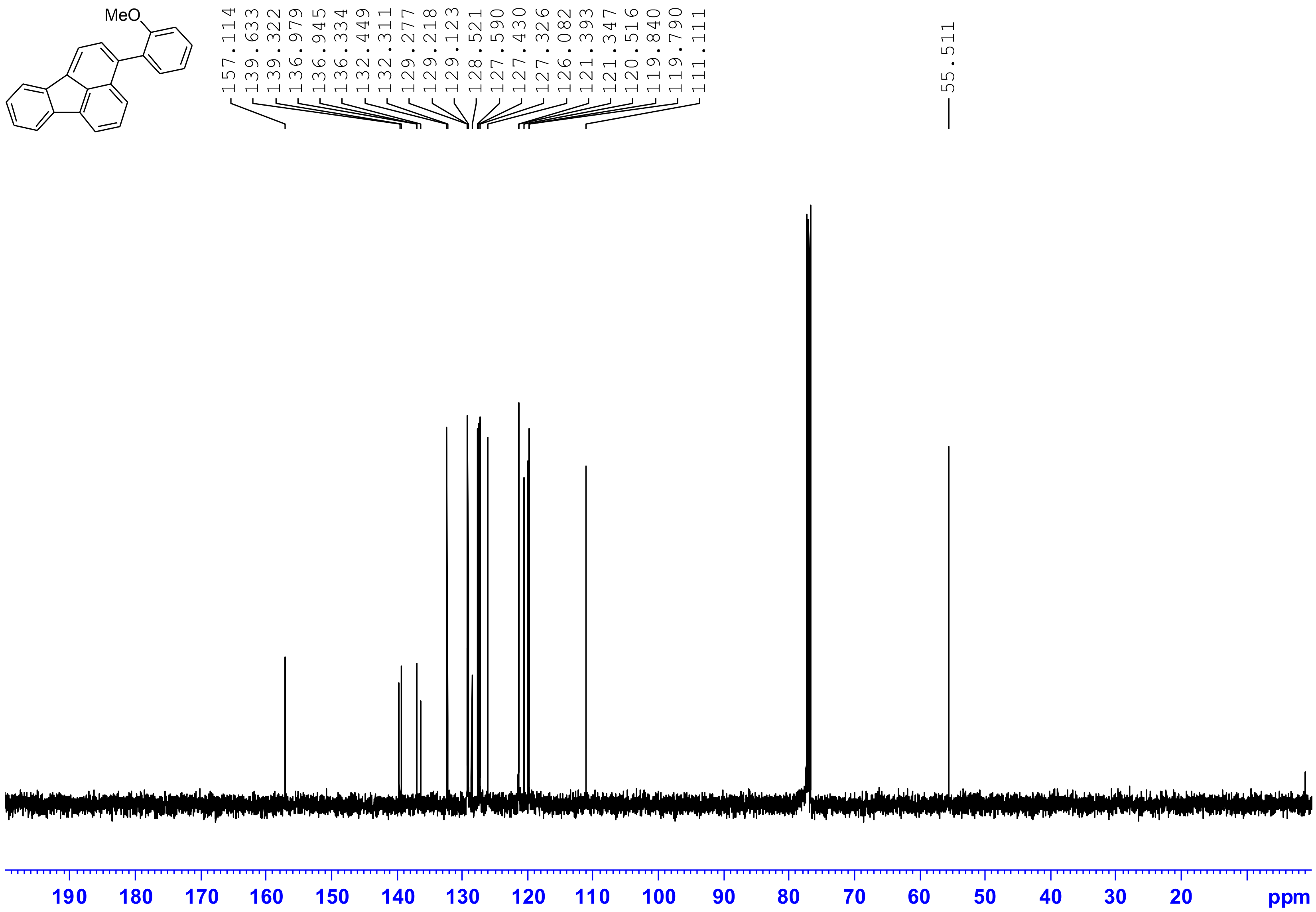
MeO
157.114
139.633
139.322
136.979
136.945
136.334
132.449
132.311
129.277
129.218
129.123
128.521
127.590
127.430
127.326
126.082
121.393
121.347
120.516
119.840
119.790
111.111
55.511
190
180
170
160
150
140
130
120
110
100
90
80
70
60
50
40
30
20
ppm

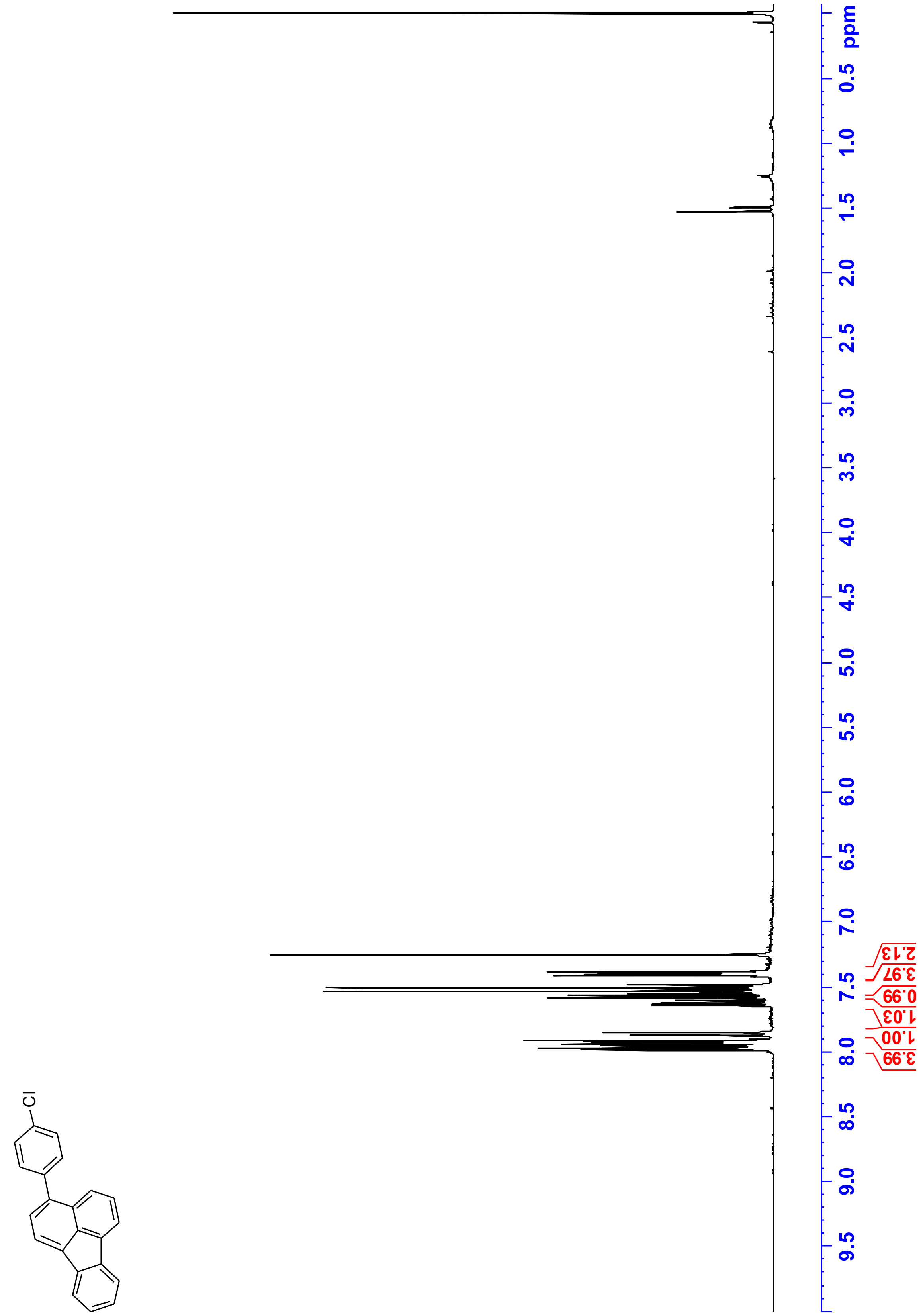
Cl
ppm
0.5
1.0
1.5
2.0
2.5
3.0
3.5
4.0
4.5
5.0
5.5
6.0
6.5
7.0
7.5
8.0
8.5
9.0
9.5
2.13
3.97
0.99
1.03
1.00
3.99

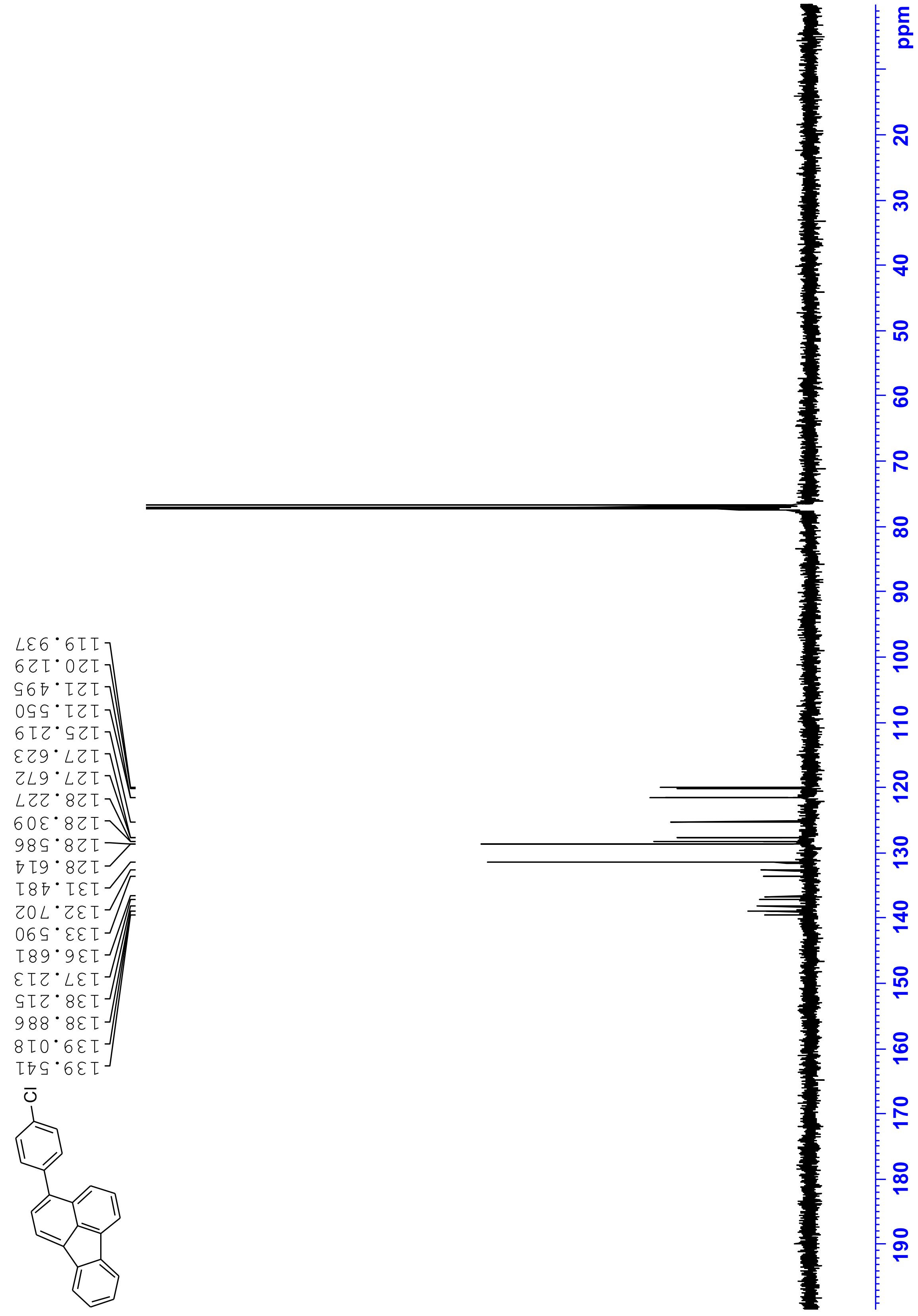
Cl
119.937
120.129
121.495
121.550
125.219
127.623
127.672
128.227
128.309
128.586
128.614
131.481
132.702
133.590
136.681
137.213
138.215
138.886
139.018
139.541
ppm

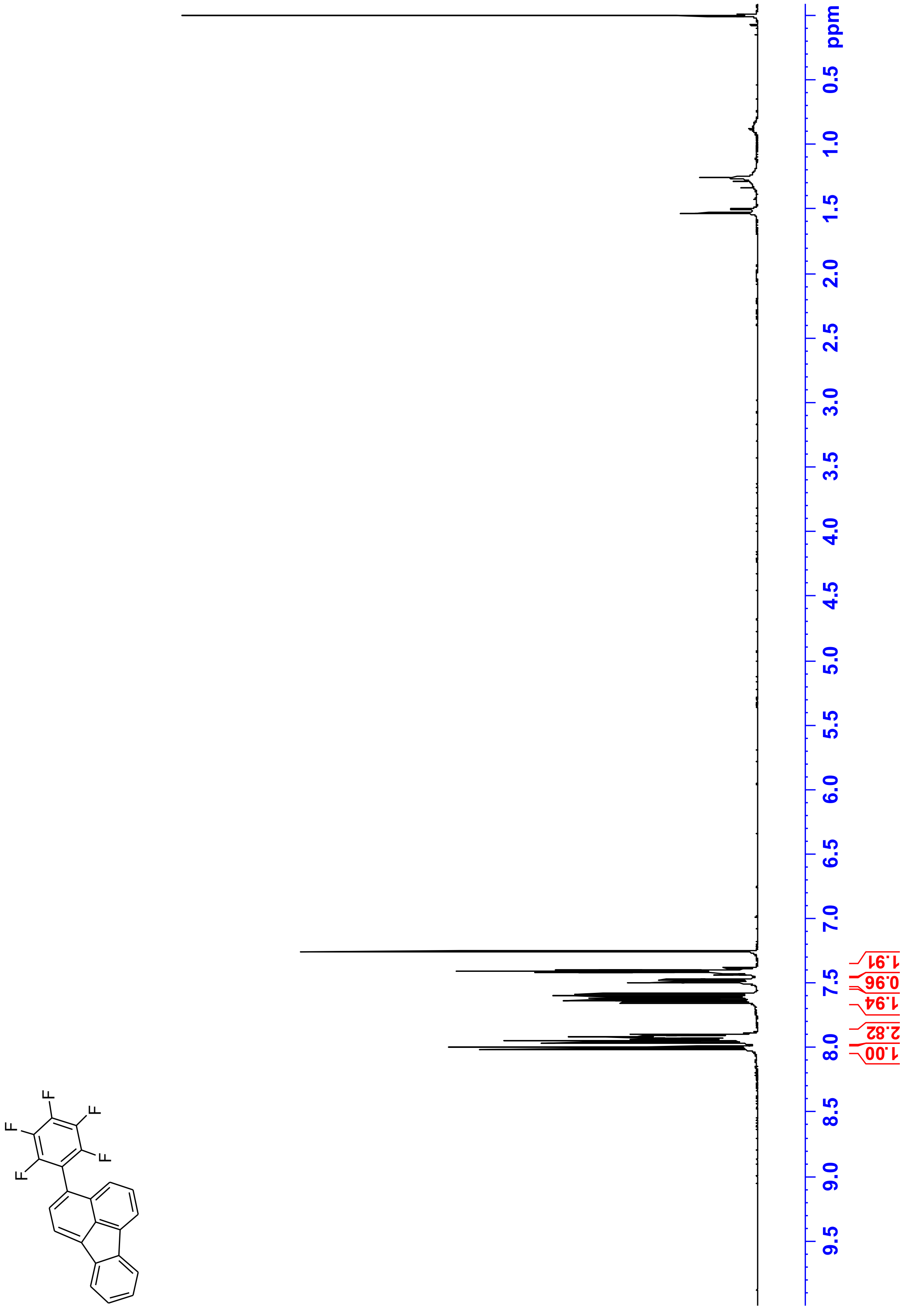
ppm
0.5
1.0
1.5
2.0
2.5
3.0
3.5
4.0
4.5
5.0
5.5
6.0
6.5
7.0
7.5
8.0
8.5
9.0
9.5
1.91
0.96
1.94
2.82
1.00
F
F
F
F
F

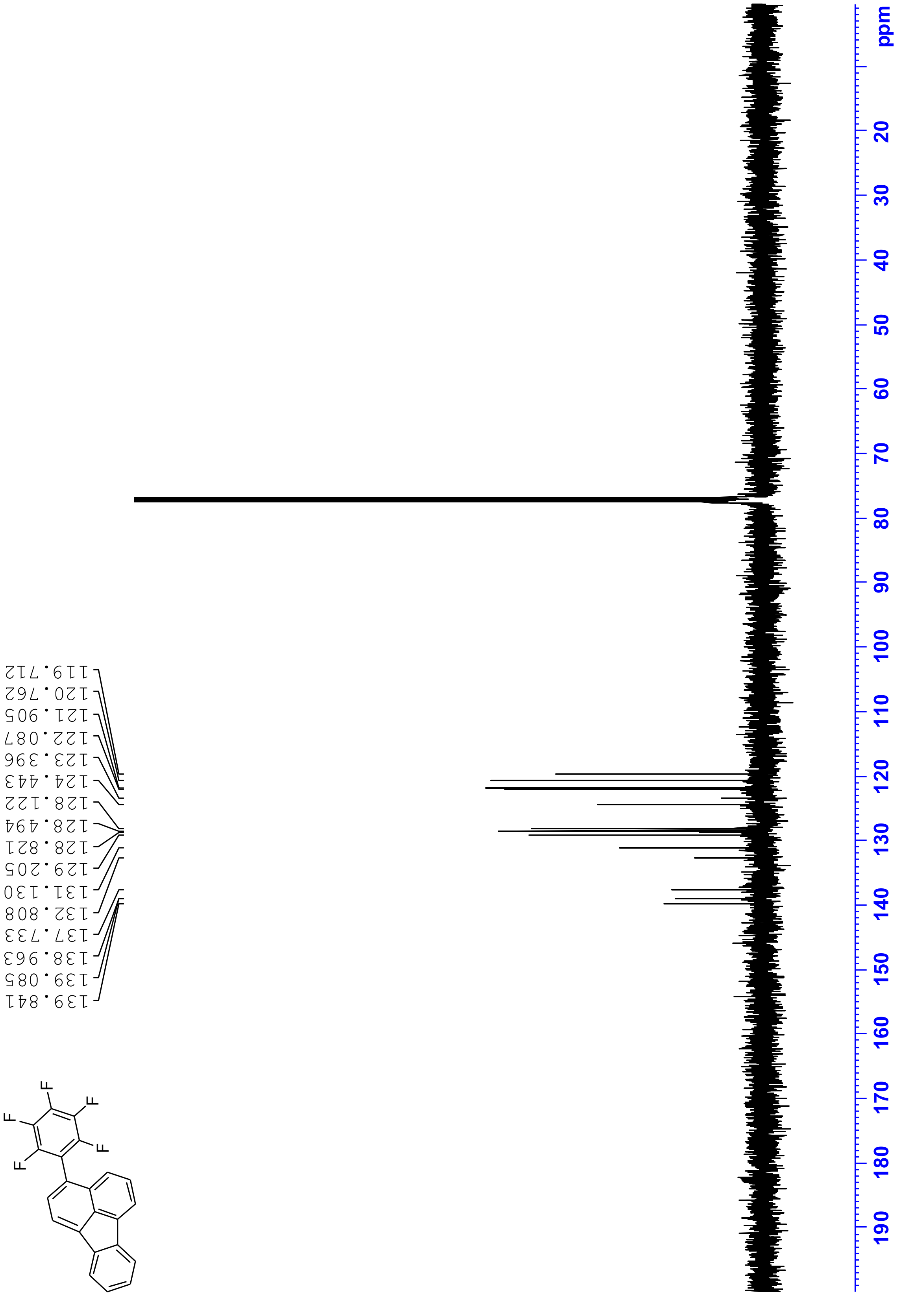
119.712
120.762
121.905
122.087
123.396
124.443
128.122
128.494
128.821
129.205
131.130
132.808
137.733
138.963
139.085
139.841
F
ppm

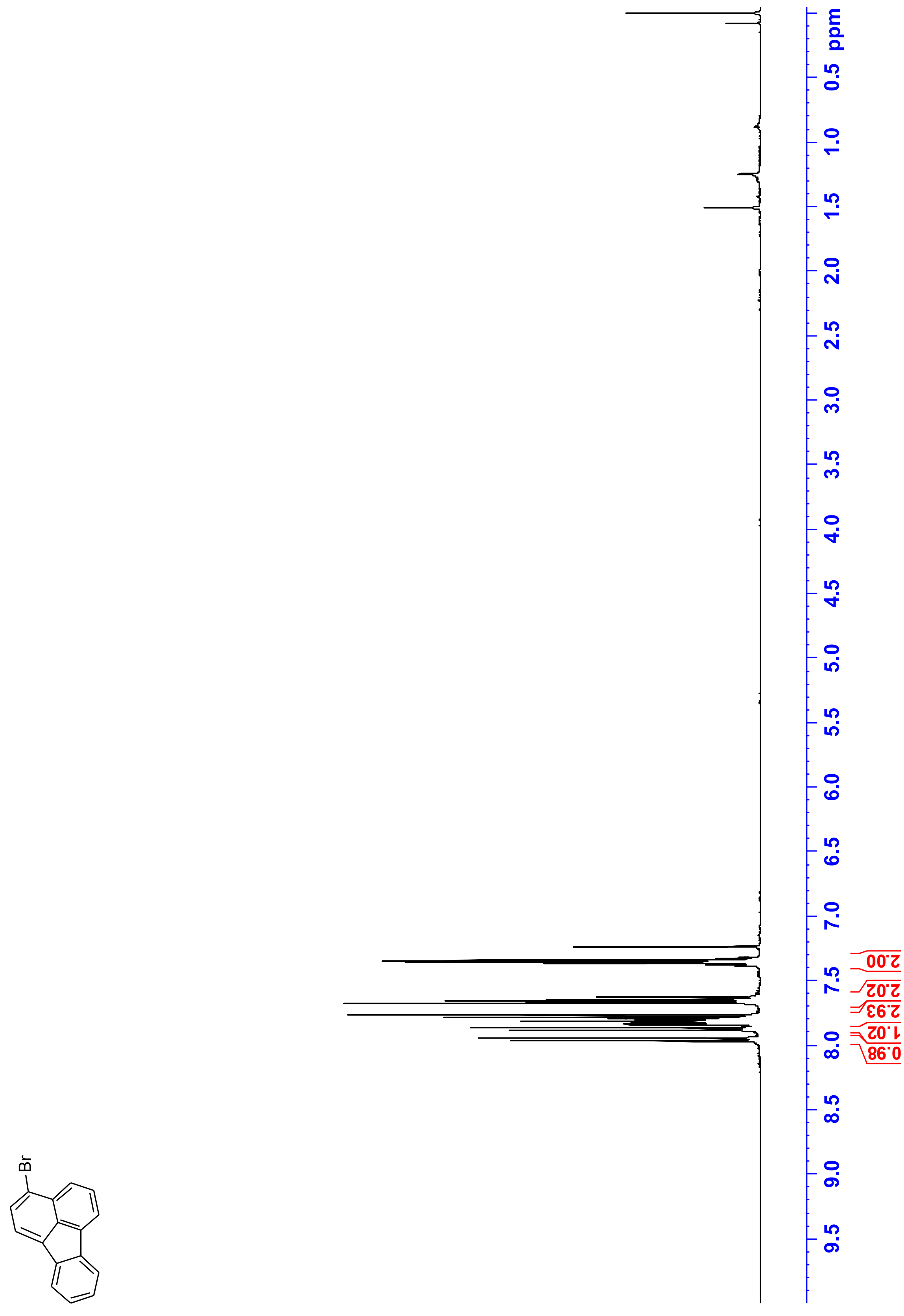
Br
ppm
0.5
1.0
1.5
2.0
2.5
3.0
3.5
4.0
4.5
5.0
5.5
6.0
6.5
7.0
7.5
8.0
8.5
9.0
9.5
2.00
2.02
2.93
1.02
0.98

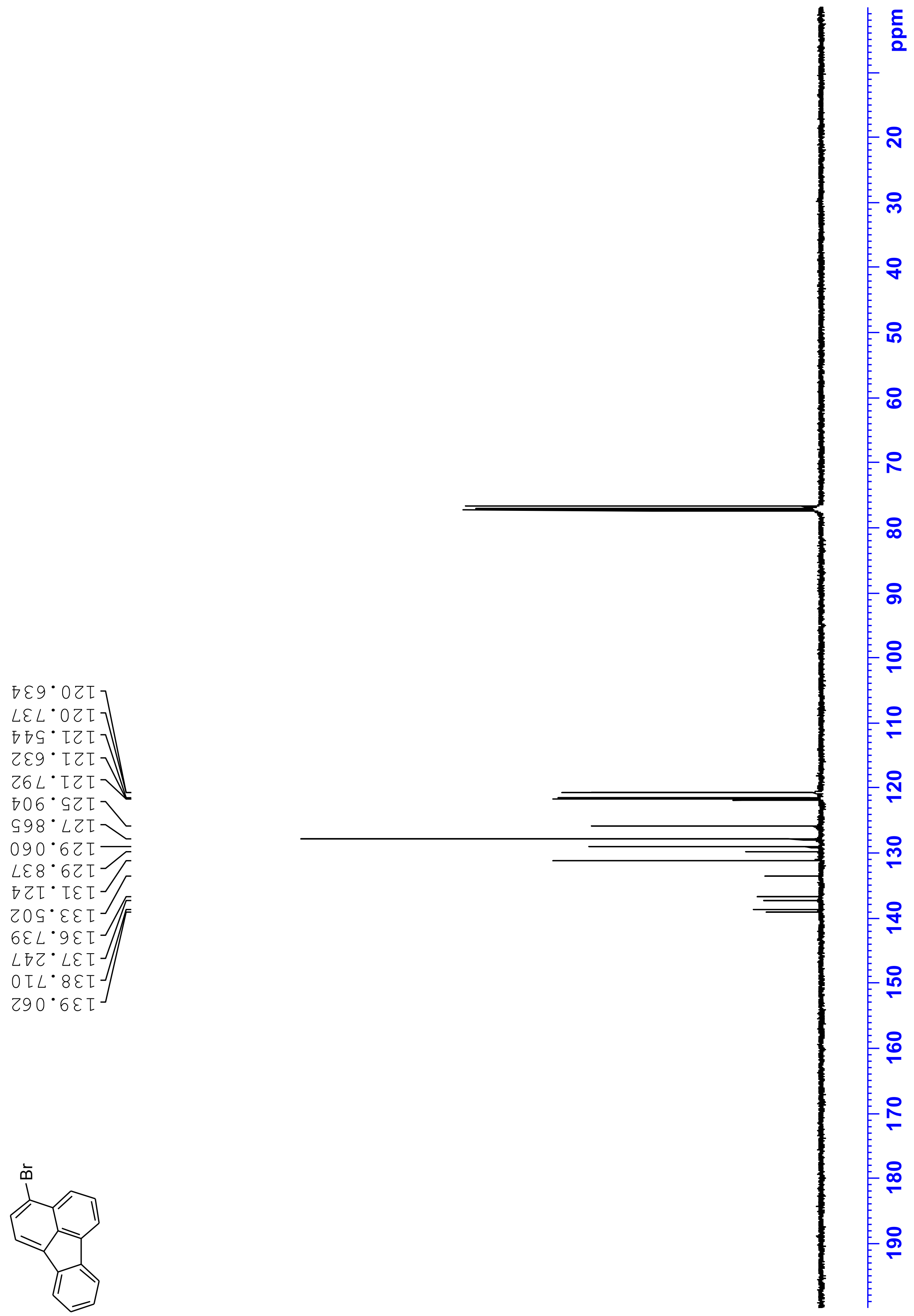
Br
120.634
120.737
121.544
121.632
121.792
125.904
127.865
129.060
129.837
131.124
133.502
136.739
137.247
138.710
139.062
ppm
20
30
40
50
60
70
80
90
100
110
120
130
140
150
160
170
180
190

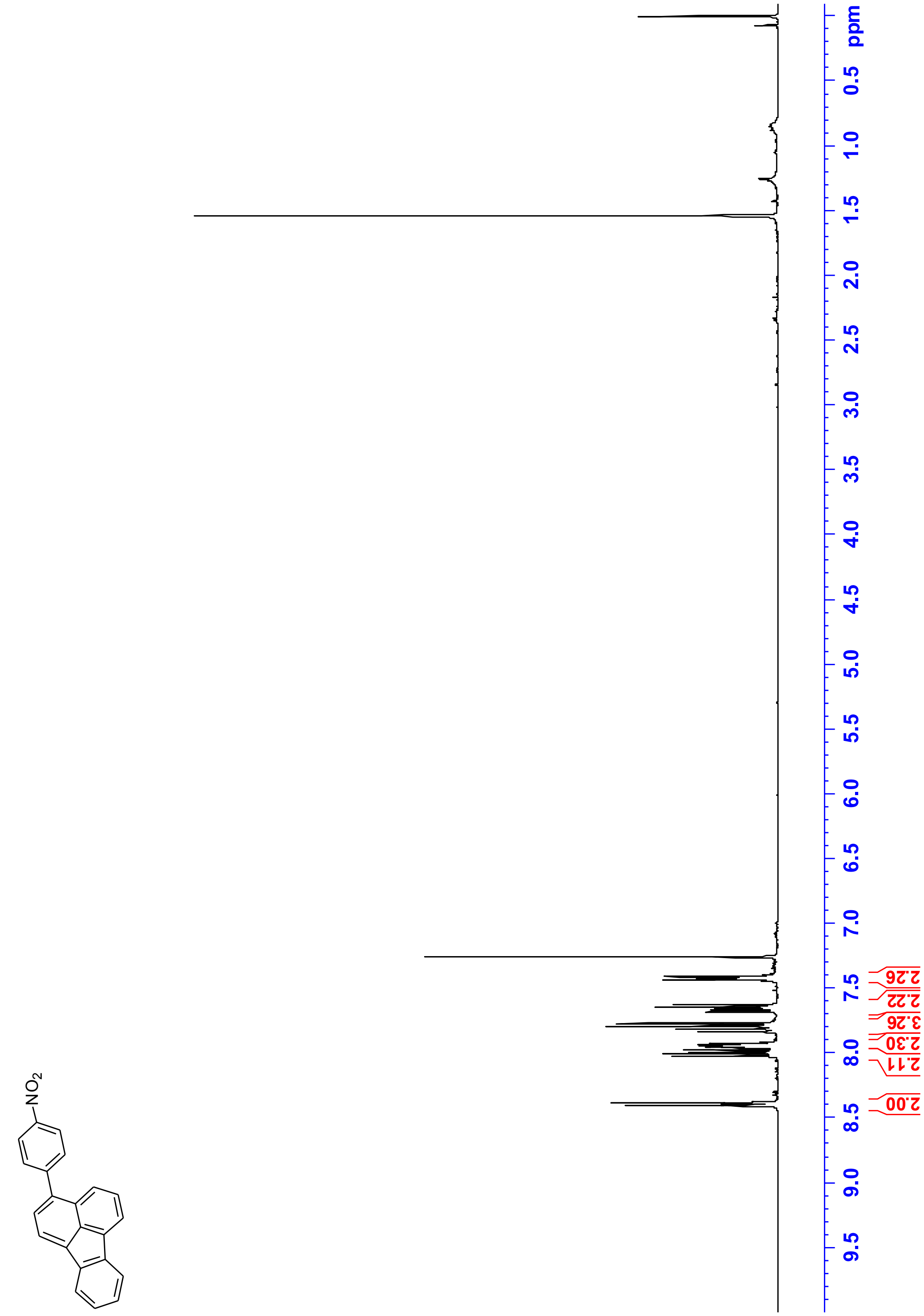

NO_2
ppm
0.5 1.0 1.5 2.0 2.5 3.0 3.5 4.0 4.5 5.0 5.5 6.0 6.5 7.0 7.5 8.0 8.5 9.0 9.5
2.26
2.22
3.26
2.30
2.11
2.00

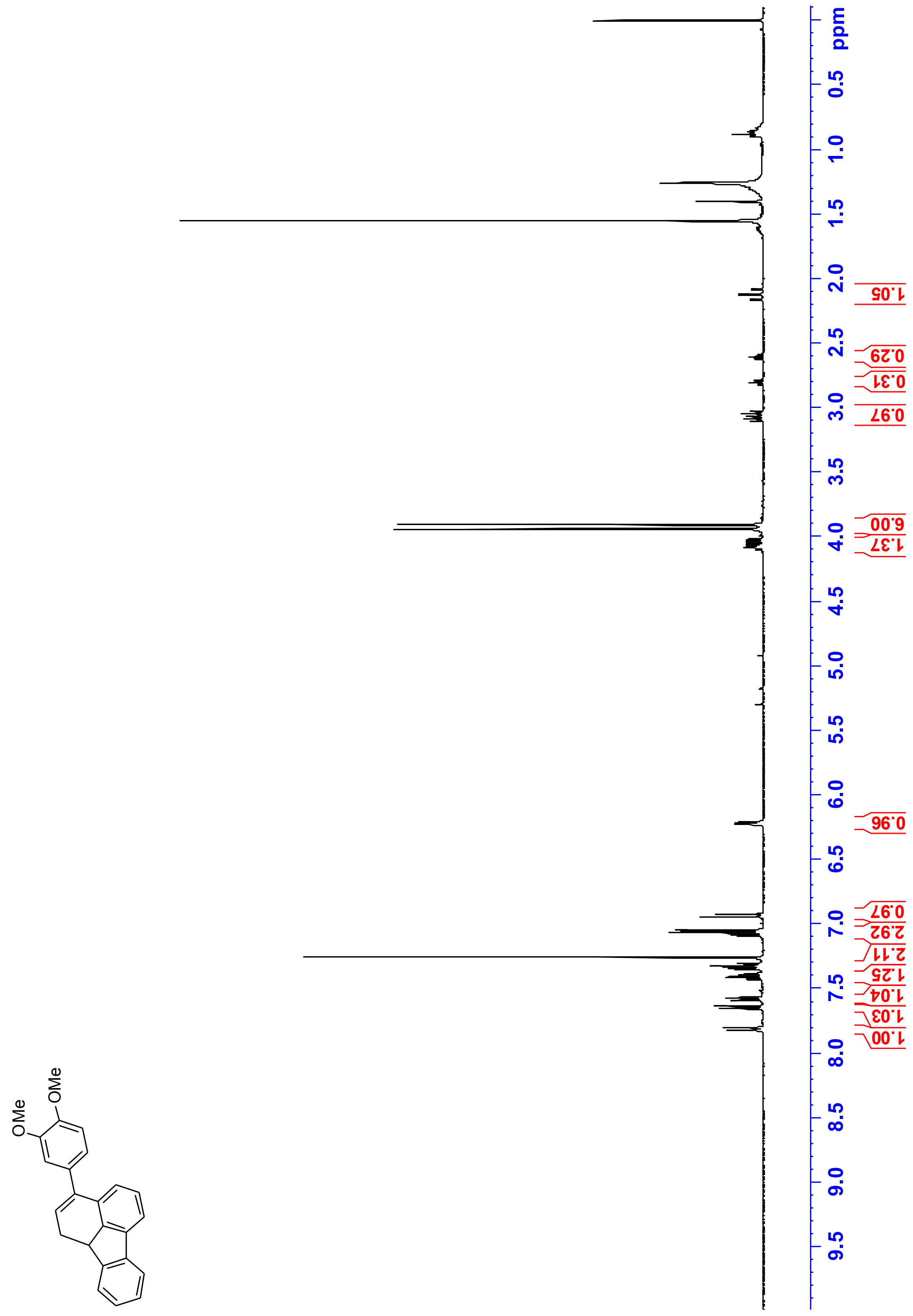

OMe
OMe
ppm
1.05
0.29
0.31
0.97
6.00
1.37
0.96
0.97
2.92
2.11
1.25
1.04
1.03
1.00

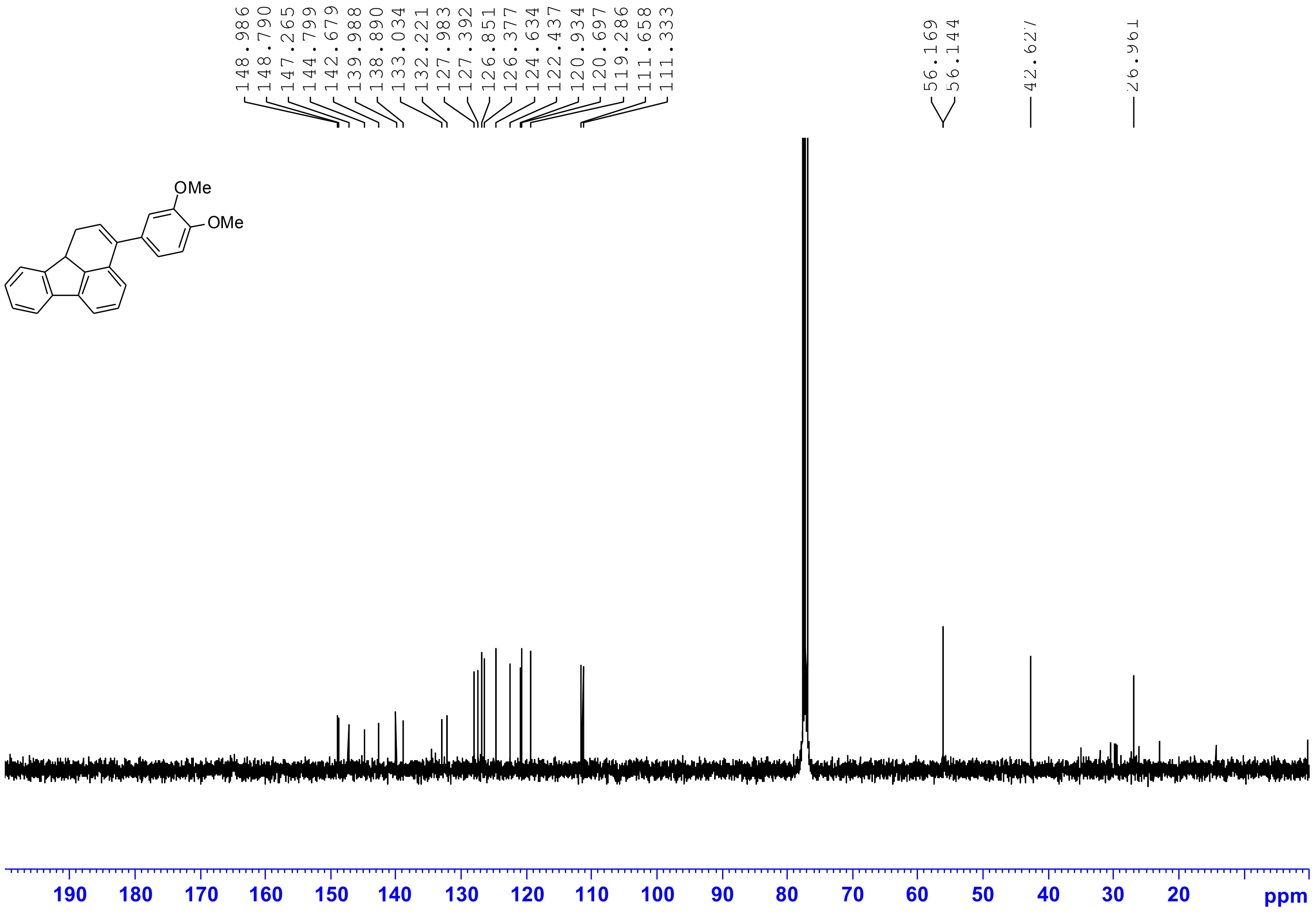
148.986
148.790
147.265
144.799
142.679
139.988
138.890
133.034
132.221
127.983
127.392
126.851
126.377
124.634
122.437
120.934
120.697
119.286
111.658
111.333
56.169
56.144
42.627
26.961
OMe
OMe
190
180
170
160
150
140
130
120
110
100
90
80
70
60
50
40
30
20
ppm

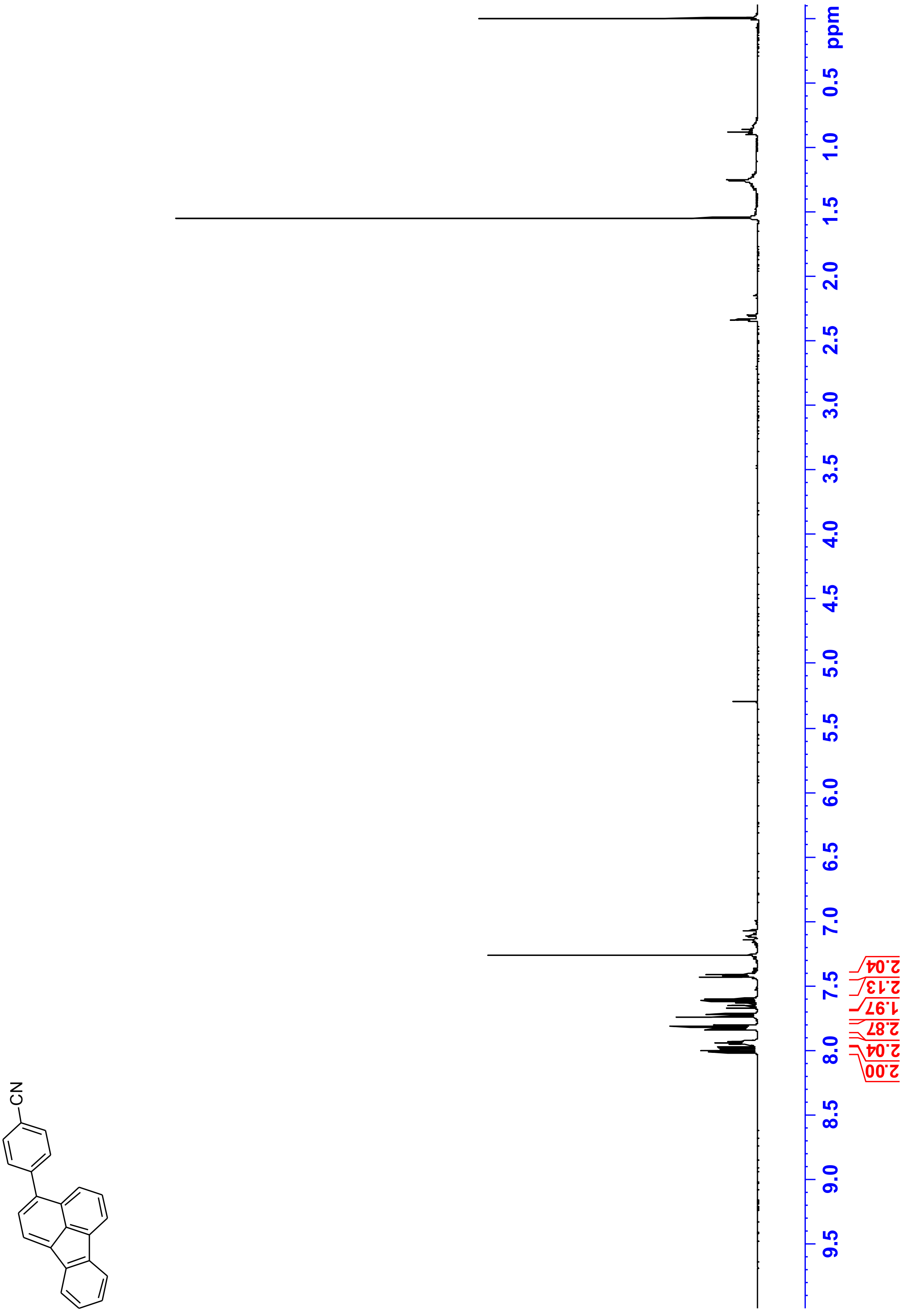
CN
ppm
0.5
1.0
1.5
2.0
2.5
3.0
3.5
4.0
4.5
5.0
5.5
6.0
6.5
7.0
7.5
8.0
8.5
9.0
9.5
2.04
2.13
1.97
2.87
2.04
2.00

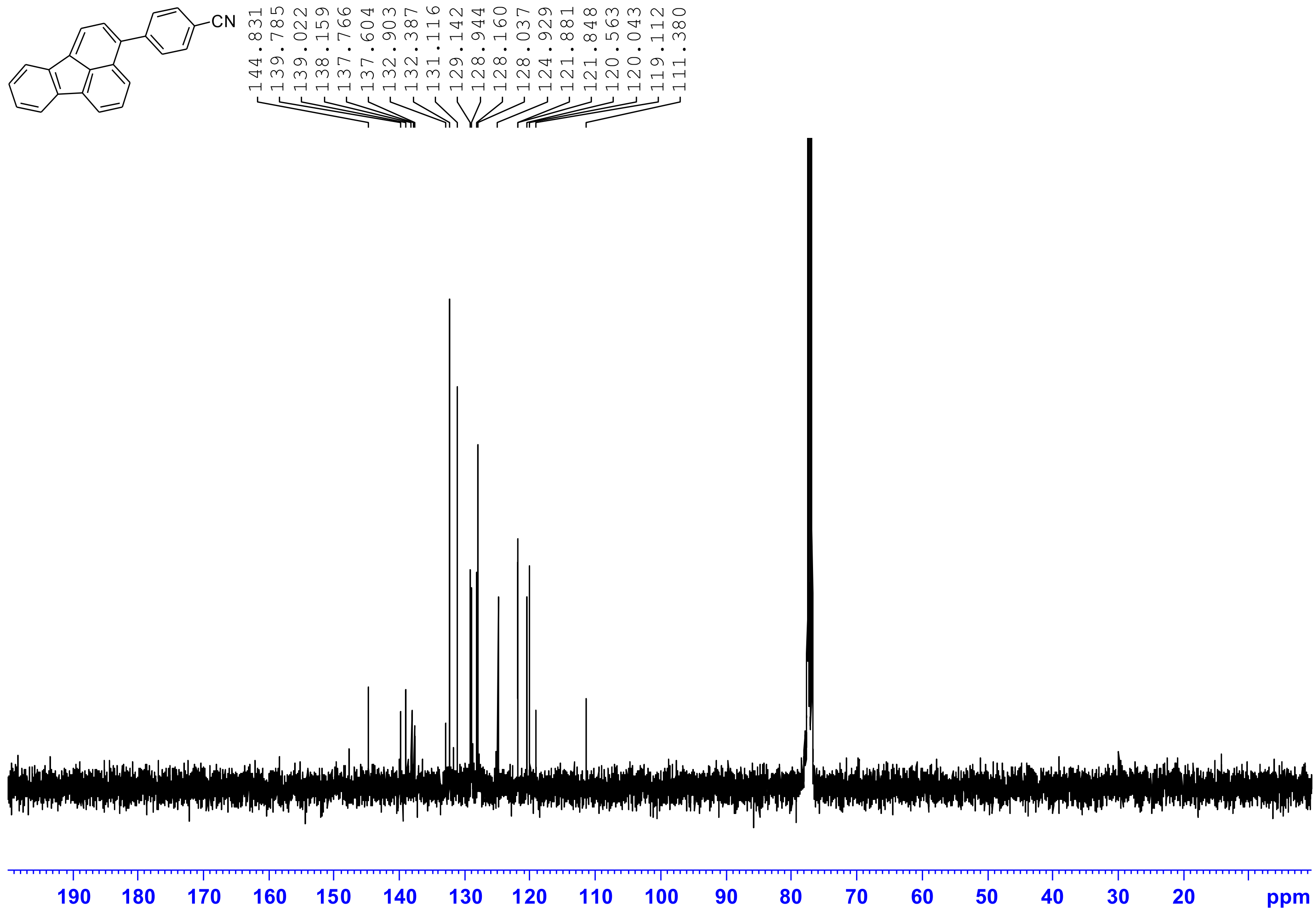
CN
144.831
139.785
139.022
138.159
137.766
137.604
132.903
132.387
131.116
129.142
128.944
128.160
128.037
124.929
121.881
121.848
120.563
120.043
119.112
111.380
190 180 170 160 150 140 130 120 110 100 90 80 70 60 50 40 30 20
ppm

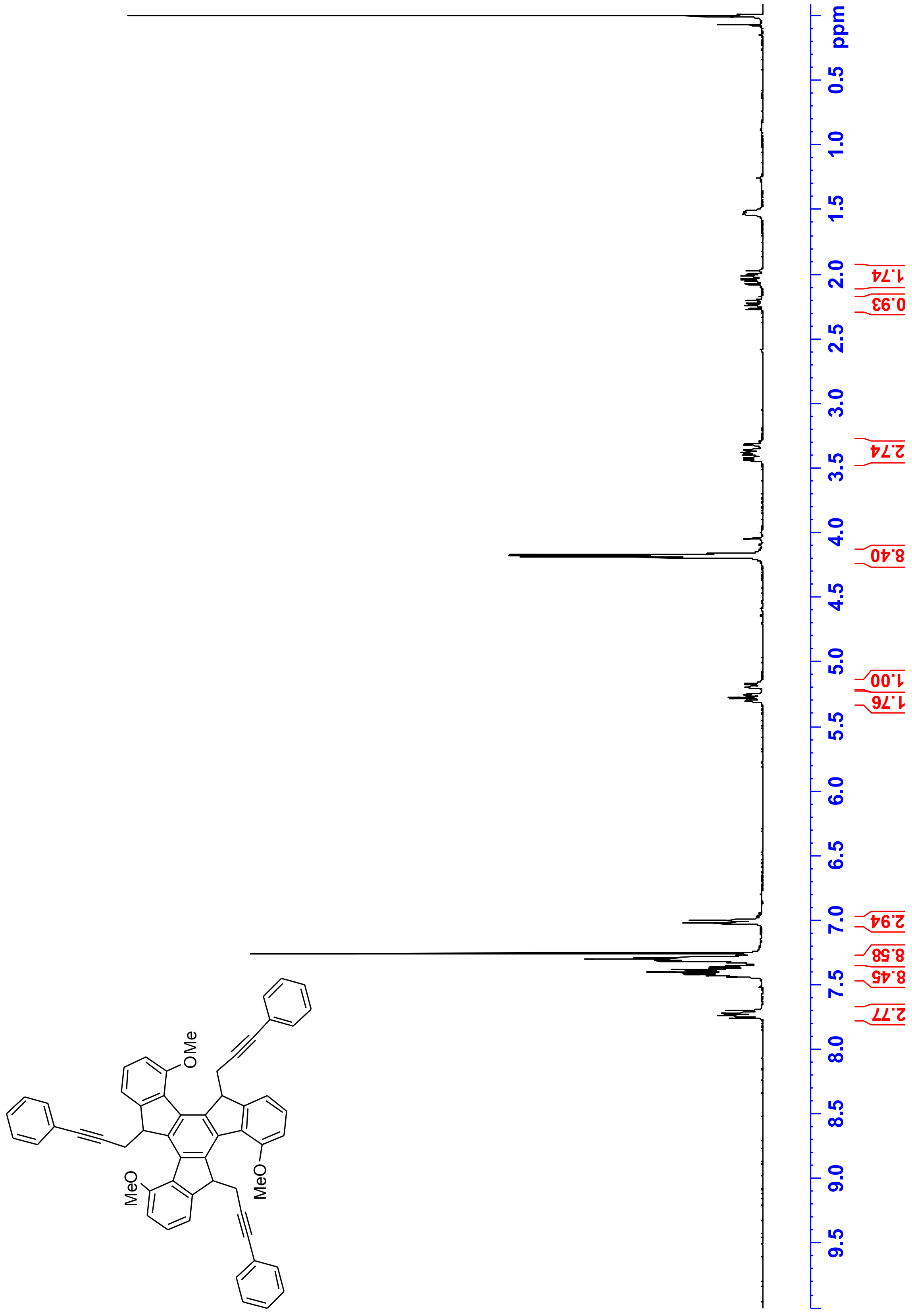
ppm
0.5
1.0
1.5
2.0
2.5
3.0
3.5
4.0
4.5
5.0
5.5
6.0
6.5
7.0
7.5
8.0
8.5
9.0
9.5
1.74
0.93
2.74
8.40
1.00
1.76
2.94
8.58
8.45
2.77
OMe
MeO
MeO

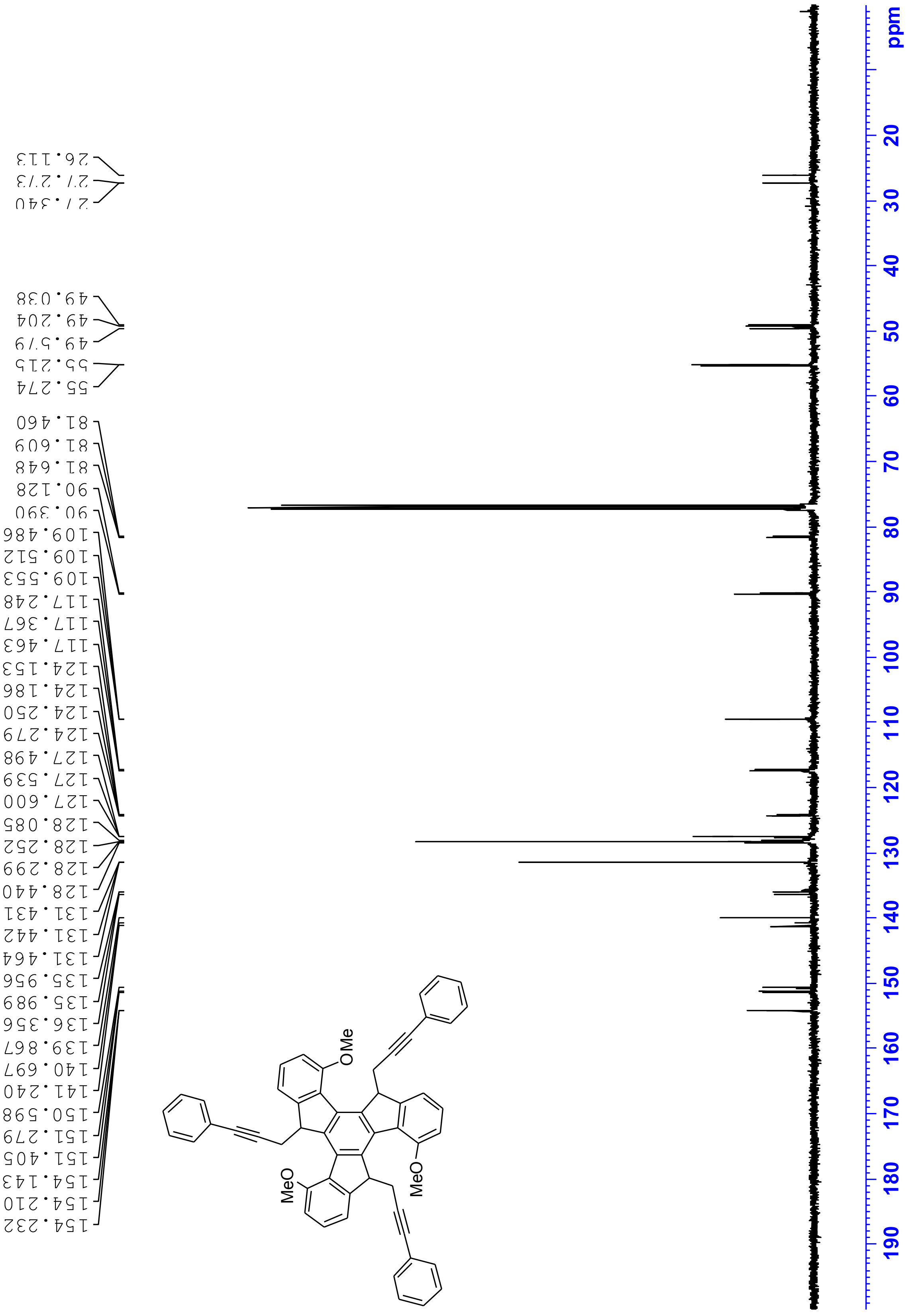
26.113
27.273
27.340
49.038
49.204
49.579
55.215
55.274
81.460
81.609
81.648
90.128
90.390
109.486
109.512
109.553
117.248
117.367
117.463
124.153
124.186
124.250
124.279
127.498
127.539
127.600
128.085
128.252
128.299
128.440
131.431
131.442
131.464
135.956
135.989
136.356
139.867
140.697
141.240
150.598
151.279
151.405
154.143
154.210
154.232
OMe
MeO
MeO
ppm
20
30
40
50
60
70
80
90
100
110
120
130
140
150
160
170
180
190

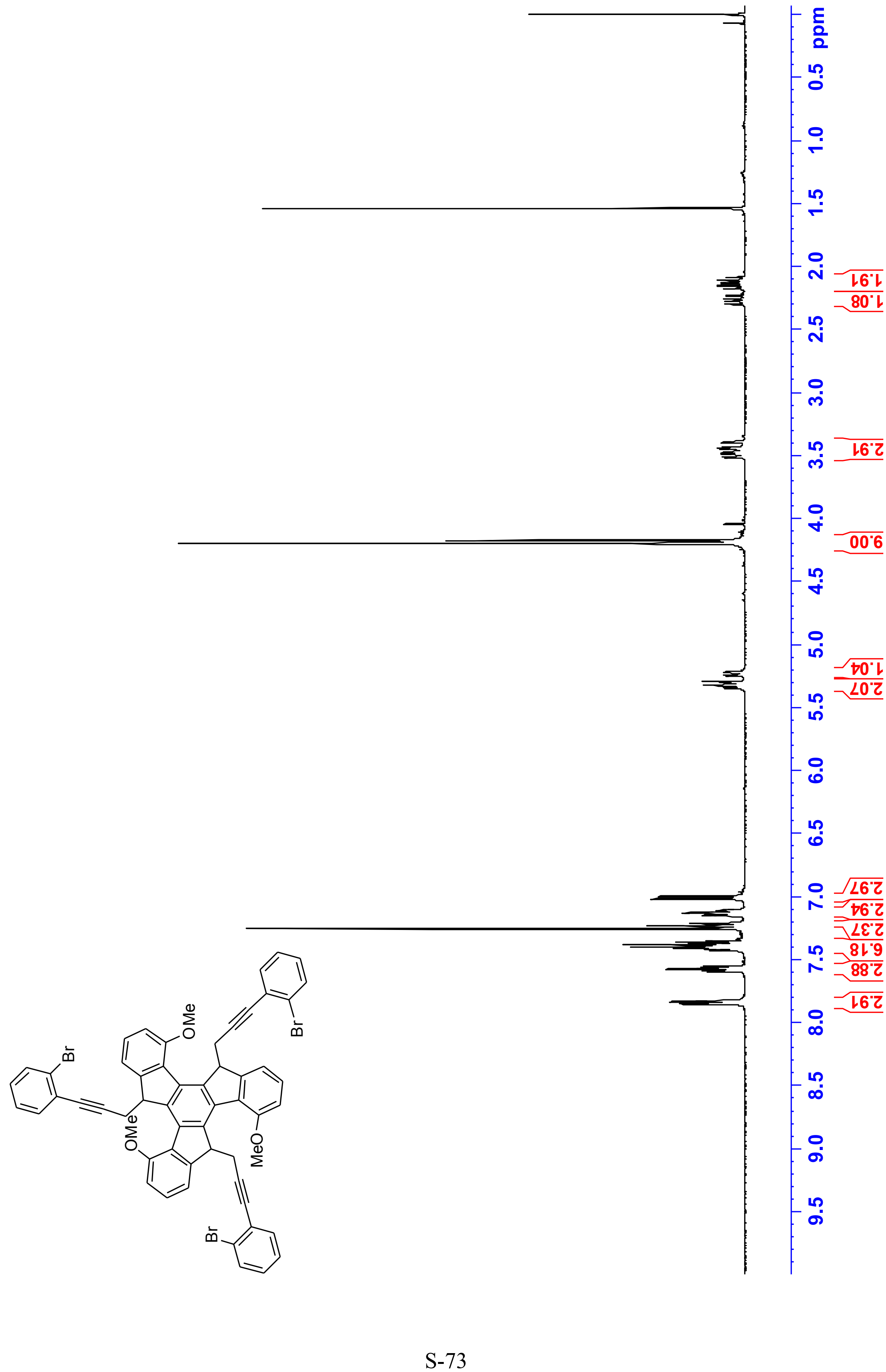
ppm
OMe
MeO
Br
1.91
1.08
2.91
9.00
1.04
2.07
2.97
2.94
2.37
6.18
2.88
2.91

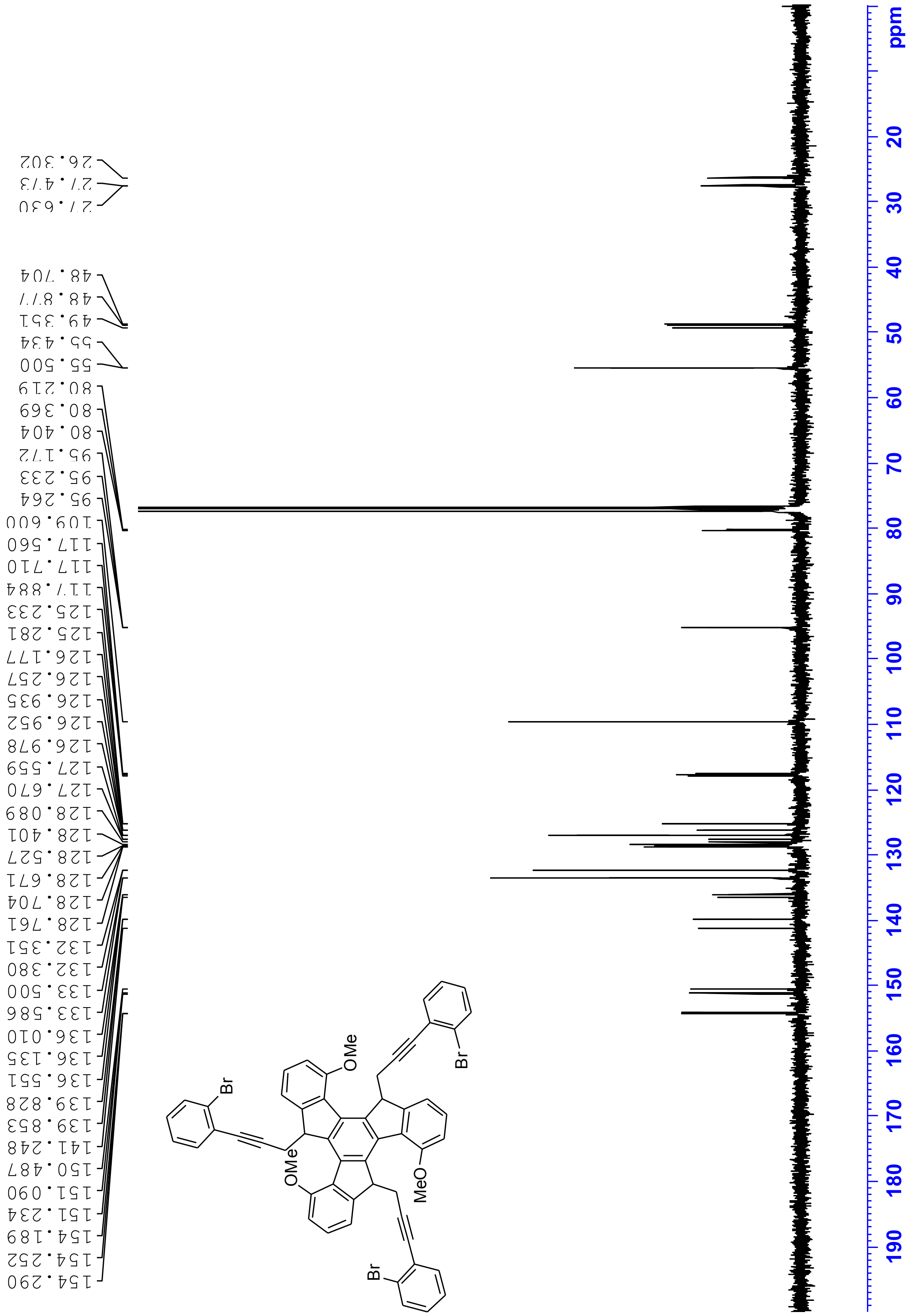

26.302
27.473
27.630
48.704
48.877
49.351
55.434
55.500
80.219
80.369
80.404
95.172
95.233
95.264
109.600
117.560
117.710
117.884
125.233
125.281
126.177
126.257
126.935
126.952
126.978
127.559
127.670
128.089
128.401
128.527
128.671
128.704
128.761
132.351
132.380
133.500
133.586
136.010
136.135
136.551
139.828
139.853
141.248
150.487
151.090
151.234
154.189
154.252
154.290
OMe
Br
MeO
ppm
20
30
40
50
60
70
80
90
100
110
120
130
140
150
160
170
180
190

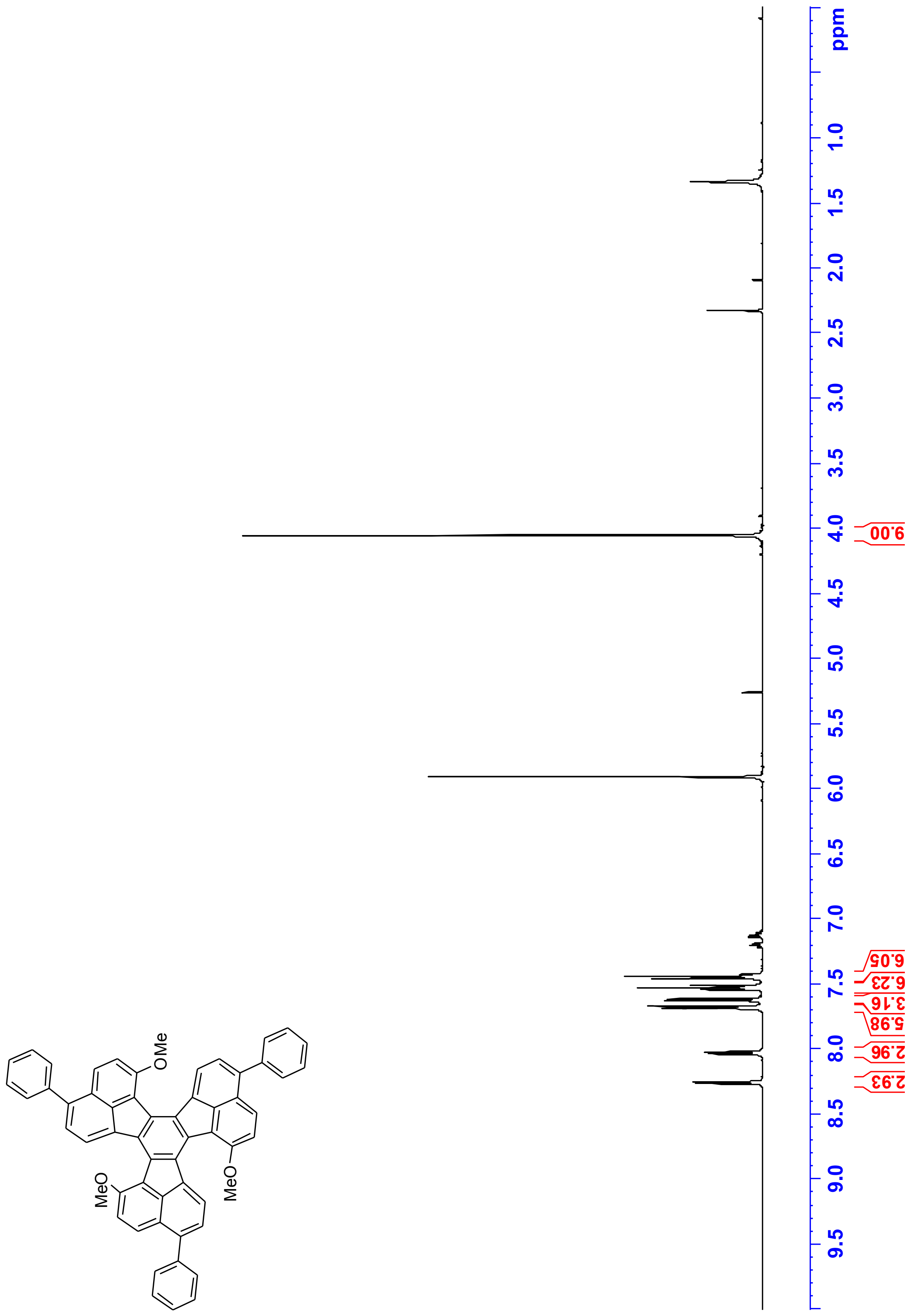
OMe
MeO
MeO
ppm
1.0
1.5
2.0
2.5
3.0
3.5
4.0
4.5
5.0
5.5
6.0
6.5
7.0
7.5
8.0
8.5
9.0
9.5
9.00
6.05
6.23
3.16
5.98
2.96
2.93

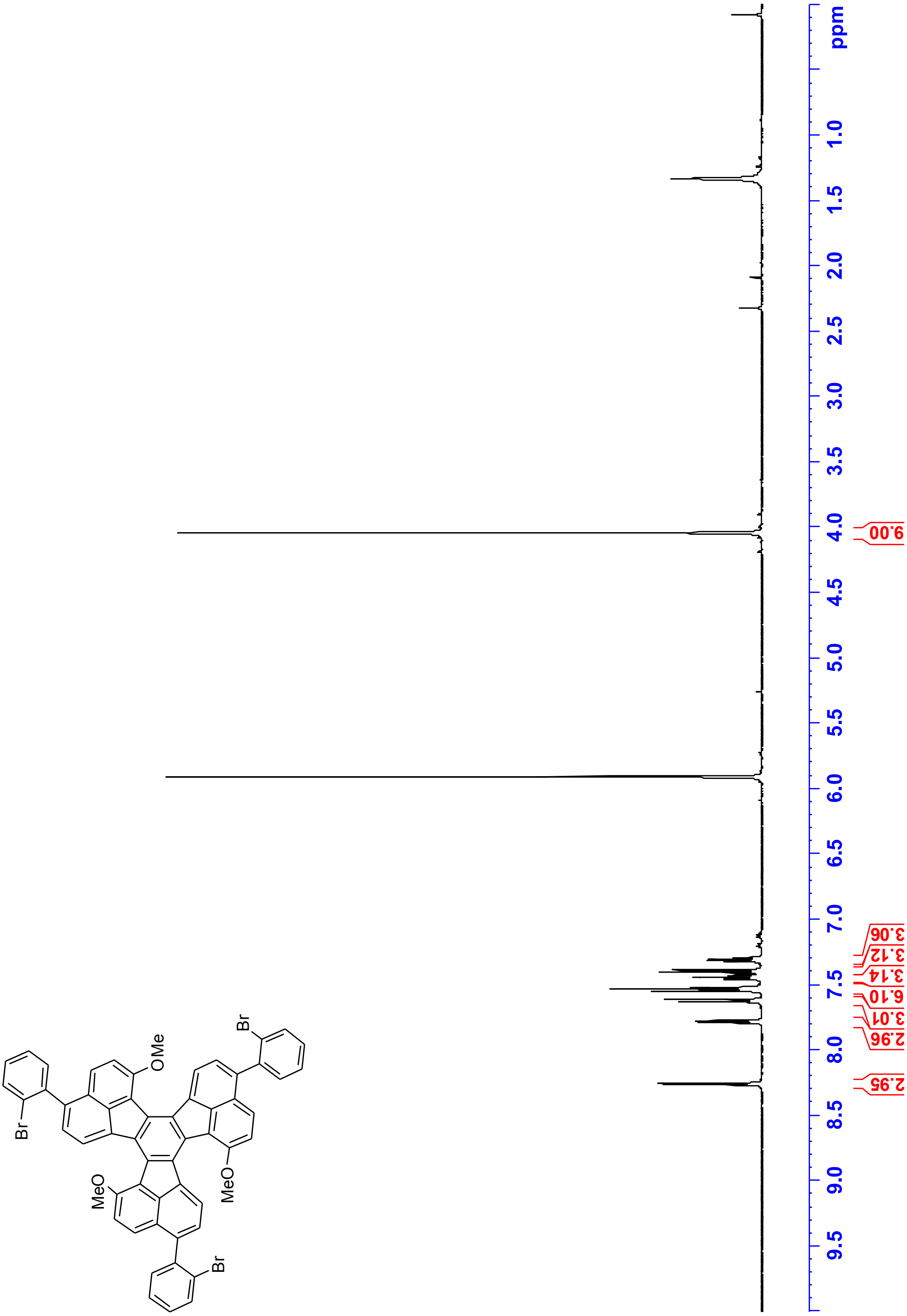

ppm
1.0
1.5
2.0
2.5
3.0
3.5
4.0
4.5
5.0
5.5
6.0
6.5
7.0
7.5
8.0
8.5
9.0
9.5
9.00
3.06
3.12
3.14
6.10
3.01
2.96
2.95
OMe
MeO
MeO
Br
Br
Br

References

1. Nieto-Oberhuber, C.; López, S.; Muñoz, M. P.; Cárdenas, D. J.; Buñuel, E.; Nevado, C.; Echavarren, A. M. *Angew. Chem., Int. Ed.* **2005**, *44*, 6146–6148.

2. Amijs, C. H. M.; López-Carrillo, V.; Raducan, M.; Pérez-Galán, P.; Ferrer, C.; Echavarren, A. M. *J. Org. Chem.* **2008**, *73*, 7721–7730.

3. Li, H. J.; Guillot, R.; Gandon, V. *J. Org. Chem.* **2010**, *75*, 8435–8449.

4. Berlman, I. B.; Wirth, H. O.; Steingraber, O. J. *J. Am. Chem. Soc.* **1968**, *90*, 566–569.

5. Ikeda, H.; Ido, M.; Funahashi, M. PCT Int. Appl. 2004, WO 2004018587. CAN 140:243295.
